# Agreement Between Heart Rate Variability - Derived vs. Ventilatory and Lactate Thresholds: A Systematic Review with Meta-Analyses

**DOI:** 10.1186/s40798-024-00768-8

**Published:** 2024-10-08

**Authors:** Valérian Tanner, Grégoire P. Millet, Nicolas Bourdillon

**Affiliations:** Quartier UNIL-Centre, Institute of Sport Sciences, University of Lausanne, Bâtiment Synathlon, Lausanne, 1015 Switzerland

**Keywords:** Heart rate variability, Ventilatory threshold, Lactate threshold, Sport, Intensity distribution

## Abstract

**Background:**

Determining thresholds by measuring blood lactate levels (lactate thresholds) or gas exchange (ventilatory thresholds) that delineate the different exercise intensity domains is crucial for training prescription. This systematic review with meta-analyses aims to assess the overall validity of the first and second heart rate variability - derived threshold (HRVT1 and HRVT2, respectively) by computing global effect sizes for agreement and correlation between HRVTs and reference – lactate and ventilatory (LT-VTs) – thresholds. Furthermore, this review aims to assess the impact of subjects’ characteristics, HRV methods, and study protocols on the agreement and correlation between LT-VTs and HRVTs.

**Methods:**

Systematic computerised searches for studies determining HRVTs during incremental exercise in humans were conducted. The agreements and correlations meta-analyses were conducted using a random-effect model. Causes of heterogeneity were explored by subgroup analysis and meta-regression with subjects’ characteristics, incremental exercise protocols, and HRV methods variables. The methodological quality was assessed using QUADAS-2 and STARD_HRV_ tools. The risk of bias was assessed by funnel plots, fail-safe N test, Egger’s test of the intercept, and the Begg and Mazumdar rank correlation test.

**Results:**

Fifty included studies (1160 subjects) assessed 314 agreements (95 for HRVT1, 219 for HRVT2) and 246 correlations (82 for HRVT1, 164 for HRVT2) between LT-VTs and HRVTs. The standardized mean differences were trivial between HRVT1 and LT1-VT1 (SMD = 0.08, 95% CI -0.04–0.19, *n* = 22) and between HRVT2 and LT2-VT2 (SMD = -0.06, 95% CI -0.15–0.03, *n* = 42). The correlations were very strong between HRVT1 and LT1-VT1 (*r* = 0.85, 95% CI 0.75–0.91, *n* = 22), and between HRVT2 and LT2-VT2 (*r* = 0.85, 95% CI 0.80–0.89, *n* = 41). Moreover, subjects’ characteristics, type of ergometer, or initial and incremental workload had no impact on HRVTs determination.

**Conclusion:**

HRVTs showed trivial differences and very strong correlations with LT-VTs and might thus serve as surrogates. These results emphasize the usefulness of HRVTs as promising, accessible, and cost-effective means for exercise and clinical prescription purposes.

**Supplementary Information:**

The online version contains supplementary material available at 10.1186/s40798-024-00768-8.

## Key Points

HRV-derived thresholds (HRVT1 and HRVT2) showed trivial standardised mean differences and very strong correlations with their respective reference thresholds (lactate and ventilatory).

Subjects’ characteristics, ergometer, or initial and incremental workload did not impact HRVTs determination.

HRVT2 is accurately determined by frequency-domain and non-linear HRV indices, and by using short increments during graded exercise tests.

**Supplementary Information:**

The online version contains supplementary material available at 10.1186/s40798-024-00768-8.

## Background

Wasserman’s 1960s studies became a milestone in exercise physiology [1–3], and since then, many research teams worldwide focused on identifying exercise thresholds using various methods. These exercise thresholds allow to establish boundaries between distinct exercise intensity domains, which is critical in exercise physiology [4–6] for evaluating training interventions, setting individual training workloads required to improve performance [7], or preventing injuries and overtraining [8–10]. These exercise thresholds also predict sports performance [4] and assess individuals’ physiological fitness, including during rehabilitation [11, 12]. They are classically identified during graded exercise tests by measuring blood lactate concentration (lactate thresholds, LTs) or gas exchange (ventilatory thresholds (VTs)) as workloads increase [13].

Blood lactate or gas exchange during graded exercise test reveal two different thresholds each (LT1, LT2, and VT1, VT2, respectively) [14] and defines the following three intensity domains [15–17]:

1. Moderate intensity domain: Aerobic energetic production, lactate production equals its removal, sustainable 6 h [17].
2. Heavy intensity domain: Lactate production exceeds physiological removal capacities. Homeostasis is disturbed [18], allowing the first threshold determination (LT1-VT1). It can be maintained for 90 min [17].
3. Severe intensity domain: Lactate and ventilation rise exponentially, allowing the second threshold determination (LT2-VT2). It can only be sustained for 15–30 min [17].

It is beyond the scope of the present review to detail the many controversies and determination methods of LTs and VTs (see [4, 6] for further details). Briefly, the gold standards for determining LTs and VTs are blood lactate and gas exchange monitoring during graded exercise tests. Briefly, *VT1/LT1* delimit moderate (zone 1) and heavy (zone 2) domains. They correspond to the first increase in V̇E vs. workload. Physiologically, greater anaerobic metabolism raises lactate, generates H^+^ buffered by HCO_3_^−^, and results in an excess CO_2_ increasing V̇E [19]. *VT2/LT2* delimit heavy (zone 2) and severe (zone 3) domains. They correspond to the second increase in V̇E vs. workload, a breakpoint in V̇E/V̇CO_2_ increase, and a decrease in P_ET_CO_2_. Physiologically, insufficient CO_2_ elimination lowers pH, increasing V̇E even more [19]. Although VT1/LT1 and VT2/LT2 are close and may be correlated [19–26], they are not always considered equivalent [8, 27–30].

However, gas exchange analysis needs sophisticated metabolic gas exchange analysers, whereas lactate monitoring necessitates invasive procedures with multiple blood sample collections [31, 32]. Additionally, these procedures require expensive equipment, specific software, and skilled operators, making them unsuitable for clinical assessment and inaccessible to a large part of the population. Finally, since various techniques used to define VTs and LTs may induce reproducibility biases, they should be interpreted and compared cautiously. Indeed, different graded exercise protocols and data analysis methods could lead to a wide range of results. Thus, more objective, non-invasive, cost-effective approaches for threshold determinations are needed.

Heart rate variability (HRV) has been proposed as an alternative non-invasive method to identify HRV thresholds (HRVTs). Indeed, a heart rate monitor may enable more specific field testing and increase applications due to its lower cost and higher availability than traditional reference thresholds (LT-VTs) [33–35]. HRV is the fluctuation in the time intervals between adjacent heartbeats [36]. HRV analyses use time-domain indices (e.g., standard deviation of NN intervals (SDNN), root mean square of successive differences (RMSSD), Poincare plot standard deviation (SD1)) which quantify interbeat interval variability, frequency-domain indices (e.g., low- (LF) and high-frequency (HF) spectral power) which estimate power distribution into frequency bands and non-linear indices (e.g., detrended fluctuation analysis alpha 1 (DFA-α1), recurrence quantification analysis (RQA)) which measure self-similarity and determinism of a sequence of cardiac interbeat intervals. The HF component’s band reflects frequency activity at rest in the 0.15–0.40 Hz range. However, to properly evaluate respiratory sinus arrhythmia (RSA) at high breathing rates, the HF component’s band is widened to 0.15–2 Hz during exercise [37]. The LF component remains in the 0.04–0.15 Hz band during exercise and is associated with a mix of sympathetic and parasympathetic modulations to the heart as well as baroreflex activity. Note that SD1 is often classified as a non-linear index. However, it is empirically and mathematically identical to RMSSD ($\:SD1=\frac{1}{\surd\:2}\bullet\:RMSSD)\:$[38].

Exercise intensity decreases total spectral energy [39–42]. LF dominates below VT1, and HF dominates above VT2 [43, 44]. Moreover, the frequency peak of the HF band (*f*HF) is well correlated to breathing frequency (BF). On the one hand, BF directly drives the RSA at low intensities, and on the other hand, BF is the most significant contributor to the V̇E curve, which tends to drive HF at high intensities [40, 43, 45, 46]. Furthermore, DFA-α1 has been recently proposed as one of the most relevant indices for HRVTs determination [47–49]. It represents the self-similarity and fractal-like composition of a series of cardiac interbeat intervals, provides information about organismic demands and network physiology during exercise [50], and is suitable for analysing nonstationary time series data like heartbeats [51]. Those HRV indices, among others, and their variations allow two HRVTs (HRVT1 and HRVT2) determination.

Based on the above-described modifications of several HRV indices during an incremental test, previous studies aimed to compare different HRV-derived thresholds to various LT-VTs during a broad range of graded exercise protocols in diverse populations. HRVTs were often proposed as a promising, cost-effective, and available alternative to classical thresholds. However, comprehensive approaches are still lacking. Indeed, previous encouraging (i.e., reporting proximity between HTVTs and LTs-VTs) results have often been obtained with small sample sizes, homogeneous populations, and specific protocols. Therefore, taking a step back and putting these results into perspective could benefit future research and significantly improve the overall applicability of HRVTs.

The recent systematic review by Kaufmann et al. [52] was a major step forward and added essential information to two previous reviews comparing HRVTs and LT-VTs [53, 54]. Nevertheless, no meta-analysis has ever computed a global effect size for correlation and agreement between reference (LT1-VT1/LT2-VT2) and heart rate variability thresholds (HRVT1/HRVT2). Furthermore, even though over 50 studies have been published on this specific topic, there has been no comprehensive effort to identify factors affecting the accuracy of HRV threshold determination in such studies. Therefore, this systematic review with meta-analyses aims to:

- Assess the overall validity of HRVTs by computing global effect sizes for agreement and correlation between heart rate variability thresholds (HRVT1/HRVT2) and reference – lactate and ventilatory – thresholds (LT1-VT1/LT2-VT2).
- Assess the impact of (1) subjects’ characteristics, (2) HRV methods, and (3) study protocols on the agreement and correlation between LT-VTs and HRVTs.
- Formulate practical recommendations for the application of HRVTs in clinical settings.

## Methods

This systematic review with meta-analyses follows the methodology proposed by the Cochrane Handbook for Systematic Reviews of Diagnostic Test Accuracy [55]. It is reported according to the Preferred Reporting Items for Systematic Reviews and Meta-Analyses (PRISMA) 2020 declaration and its extensions [56–59].

### Search Strategy

The search was conducted between March and August 2023. Systematic computerised searches were performed using eleven electronic databases (Cochrane Library, EBSCO, Embase.com, Google Scholar, Ovid, ProQuest, PubMed, Scopus, SPORTDiscus, Virtual Health Library, and Web of Science). The leading search strategy was ((“heart rate variabilit*” OR “heartrate variabilit*” OR HRV OR “detrended fluctuation analys*” OR DFA OR “time varying analys*” OR “fractal correlation propert*” OR “recurrence quantification analys*”) AND (“ventilatory threshold*” OR “lactate threshold*” OR “aerobic threshold*” OR “anaerobic threshold*” OR “intensity threshold*”)) OR (“heart rate variability threshold*” OR “heartrate variability threshold*” OR HRVT OR HRVTS OR HRVT1 OR HRVT2). No limits were used during electronic database searching. The search strategy was adapted as necessary for each database, and all database queries were peer-reviewed by a health information specialist. Exact search strategies, sub-databases queried, date of the query, and number of results for each electronic database are listed in Online Resource 1. Moreover, references included in three previous reviews [52–54] were manually assessed for eligibility.

### Eligibility Criteria

The pre-established eligibility criteria were the following ones: *study type*: full-length original articles in peer-reviewed journals and “grey” literature (thesis, dissertation, conference abstract); *population*: human subjects regardless of age, sex, weight, health, or training status; *intervention*: determination of HRVT1 and/or HRVT2 and LT-VTs simultaneously during an incremental exercise test, HRVTs, and LT-VTs determination methods must be clearly detailed, high-quality RR series from a validated HRV device must be used since the recording device affects HRV precision [60], detailed explanations of the graded exercise protocol used must be provided; *comparison*: statistical comparison of HRVT1 and/or HRVT2 vs. corresponding LT and/or VT; *outcome*: all studies comparing HRVT to LT or/and VT were included, regardless of the units used for thresholds values or the HRV variables used. Publications in English, French, Italian, and German were included, and no date restriction was applied. Studies were excluded if their full texts were unavailable, experimental protocol description was unclear, experimental data were incomplete, and the corresponding authors did not address this after being contacted. The studies were grouped for analysis according to the determined HRV threshold(s) (HRVT1 or HRVT2) and according to the statistical analysis done (agreement or correlation). Four distinct groups (i.e., agreement, and correlation between HRVT1 and LT1-VT1; agreement, and correlation between HRVT2 and LT2-VT2) were thus obtained, with some studies present in several groups if the corresponding results were reported.

### Review Process

All results of the search as mentioned above were imported into EndNote^®^ (20.5, Clarivate, Philadelphia, PA, USA) for deduplication and uploaded in DistillerSR^®^ (2.41.0, Evidence Partners, Ottawa, ON, Canada) for the review process and data extraction. First, one author (VT) screened titles and abstracts thoroughly for relevancy with a low inclusion threshold. Since only one author screened titles and abstracts, wrong exclusions were the primary concern. Each exclusion reason during the title and abstract screening was therefore documented. In addition, the DistillerSR’s “Check for Screening Errors” tool was used to identify potentially incorrectly excluded references. This works by training itself multiple times using the previously screened references in a 10-fold k-fold cross-validation method [61] and allows for double-checking exclusion. This tool’s false exclusion rate [62, 63] is comparable with human performance [64–66] and has thus been suggested as a second screener alternative [67–71]. The remaining studies’ full texts were independently screened by two authors (NB and VT) using the pre-established eligibility criteria. In cases of disagreement, consensus was reached by discussion. As recommended [56], each exclusion reason during full-text screening was documented in Online Resource 2.

### Data Extraction

The following data from the selected studies were extracted using specifically designed and standardised DistillerSR^®^ forms: *general information*: author, journal, year, country; *population*: age, sex, weight, height, BMI, V̇O_2_max, health status, subject selection process, eligibility, exclusion criteria and sample size; *intervention*: HRV recording device (e.g., ECG, Polar H10), HRV data analysis process, HRV recording device type (e.g., ECG, chest strap), HRV software (e.g. Kubios, Matlab), number of comparisons between HRVTs and LT-VTs, type of ergometer (e.g. cycling, treadmill), treadmill modality (e.g. running, Nordic-walking), start workload, start slope, increment workload, increment duration, increment slope; HRVT, LT and/or VT determination type (i.e., visual or computed); HRVT, LT and/or VT exact determination methods; *comparison*: statistical agreement (p-value) and correlation (Pearson’s r) between each corresponding HRVT determination method and LT-VT determination method; *outcome*: all reported outcomes (heart rate, power, speed, V̇O_2_max, and/or kg expressed as absolute and/or as percentage of maximum value) and their standard deviation at all thresholds (HRVT, LT and/or VT) were extracted.

### Methodological Quality Assessment

The methodological quality of the included studies was assessed using the QUADAS-2 and the STARD_HRV_ tools. The QUADAS-2 [72], which recommends evaluating risks of bias (RoB) and applicability of primary diagnostic accuracy studies, was used to assess the RoB in included studies. It addresses four specific domains: subjects’ selection, index test, reference standard, and flow and timing. Each domain was evaluated as “low”, “high”, or “unclear” regarding RoB and concerns for applicability. The HRV-specific version of the original Standard for Reporting Diagnostic Accuracy Studies (STARD_HRV_) was used to assess the methodological quality of HRV methodology [73, 74]. It includes 25 parameters with a maximum of 25 points. The modifications proposed by Kaufmann et al. [52] to items 1, 9, 19, and 21 were used to suit the present systematic review better.

### Effect Size Calculation and Data Analysis

Based on the extracted data, the following four distinct meta-analyses were performed to assess the agreement and correlation between HRV and reference thresholds: (1) agreement and (2) correlation between HRVT1 and LT1-VT1; (3) agreement and (4) correlation between HRVT2 and LT2-VT2.

For agreement meta-analyses (1 and 3), standardised mean difference (SMD) was used as the effect size index, with positive values indicating that HRVT was higher than LT-VT, negative values indicating that HRVT was lower than LT-VT, and values close to 0 suggesting high agreement between reference and HRV thresholds determination. The standardised difference in means was classified as trivial (< 0.2), low (0.2–0.5), moderate (0.5–0.8), and high (> 0.8) [75, 76]. For correlation meta-analyses (2 and 4), Pearson correlation coefficient (r) was used as the effect size index with values close to 1 indicating a strong correlation between reference and HRV threshold determination. The correlation assessed by Pearson’s r was classified as poor (< 0.2), fair (0.2–0.5), moderate (0.6–0.7), and very strong (> 0.8) [77].

Since included studies differ in population and assessed intervention, different true effect sizes may underlie different studies [78]. Consequently, our four meta-analyses used a random-effect model to generate an overall mean effect size and 95% confidence interval (CI). Indeed, this model considers two crucial and distinct sources of variance in the included studies: the error within each study’s effect size estimate and the variation in true effects across all studies. The inverse variance method determined study weights by minimising both variance sources [78, 79]. The studies within each meta-analysis are assumed to be a random sample from a universe of potential studies, and this analysis will be used to make an inference about that universe [55, 79–82], allowing us to carry out comprehensive meta-analyses despite the heterogeneity of the included studies. Considering that some studies reported several outcomes for a single comparison between HRVT and LT-VT and even several different comparisons between HRVT and LT-VT, the most conservative standard procedures were used to adjust for the correlation between effects nested within studies [78, 80, 83]. The DerSimonian and Laird method [84] was used to estimate the variance between studies.

When necessary, the units of the various outcomes were converted as follows: time (s), power (W), V̇O_2_max (mL · min^−1^ · kg^−1^). Effects size computations and analyses were made using Comprehensive Meta-Analysis Version 4 (Borenstein, M., Hedges, L., Higgins, J., & Rothstein, H., Biostat, Englewood, NJ 2022). Forest plots were made using Microsoft Excel (Microsoft Office 365). Data were presented as mean ± 95% CI. Statistical significance was determined a priori at α = 0.05.

### Heterogeneity Analysis

The Cochrane Q-test (heterogeneity significance), I^2^ statistic (proportion of variance between studies that can be attributed to true variation in effect sizes rather than sampling error), and prediction intervals (dispersion of effect sizes) assessed the statistical heterogeneity between studies in each meta-analysis. I^2^ values were classified as low (25%), moderate (50%), and high (75%) levels of heterogeneity [85]. In cases of significant heterogeneity (Q-test p value < 0.05), causes were explored by subgroup analysis (categorical moderator) and meta-regression (continuous moderator) regarding subjects’ characteristics, incremental exercise protocols, and HRV methods. Subgroup analyses were conducted using a combination of study-level variables (each study included in one subgroup only) and within-study contrasts (study included in more than one subgroup) [56], depending on the analysed moderator. Subgroups were compared using statistical test for interaction and pairwise comparison (z-test).

The age groups were defined to determine homogeneous groups with the subjects of the included studies (≤ 16, 17–35, 36–54, ≥ 55). Weight classes were established according to the World Health Organization (< 18.5 kg/m^2^, Underweight; 18.5–24.9 kg/m^2^, Healthy weight; 25–29.9 kg/m^2^, Overweight; 30–34.9 kg/m^2^, Obesity class I; 35–39.9 kg/m^2^, Obesity class II; ≥ 40 kg/m^2^, Obesity class III) [86]. Training status was classified according to the subjects’ V̇O_2_max (mL · min^−1^ · kg^−1^) based on the ACSM guidelines (< 25, Very poor; 25–34, Poor; 35–44, Fair; 45–54, Good; 55–64, Superior; ≥ 65, Athlete) [87]. When needed, the exercise intensity was converted into the Metabolic Equivalent of Task (MET) using the ACSM’s Metabolic Calculations Handbook recommendations [88]. Initial and incremental workloads were classified based on [89] as Light (< 3 MET), Moderate (3–6 MET), or Vigorous (> 6 MET).

### Risk of Bias Assessment

The risk of bias (RoB) for each of the four meta-analyses was assessed by visual inspection of funnel plots for asymmetry [90], fail-safe N test if the overall outcome was significant [91], Egger’s test of the intercept [92] and the Begg and Mazumdar rank correlation test [93]. The funnel plots were created by plotting the effect size (SMD and Fisher’s Z) against standard error. Furthermore, a leave-one-out sensitivity analysis was completed by sequentially excluding each study to identify potential outliers in included studies. A study was considered an outlier if the leave-one-out pooled effect size was not within the 95% CI of the original pooled effect size.

### Certainty Assessment

The Grading of Recommendations Assessment, Development, and Evaluation (GRADE) guidelines [94] assessed the certainty of evidence presented in this systematic review’s four meta-analyses. The five GRADE domains ((1) study limitations, (2) consistency of effect, (3) imprecision, (4) indirectness, and (5) publication bias) and the related checklist [95, 96] were used to rate the evidence as high, moderate, low, or very low.

**Table 1:** Main characteristics of included studies

Author	Year	Title	Sample size	Mean age [y]	Mean BMI [kg/m^2^]	Mean V̇O_2_max [mL · min^− 1^ · kg^− 1^]	LT-VT used
Anosov et al. [46]	2000	High-frequency oscillations of the heart rate during ramp load reflect the human anaerobic threshold	22	24	21	34	VT2
Babecki et al. [97]	2021	Détermination des seuils ventilatoires par la variabilité de la fréquence cardiaque: techniques, méthodes et automatisation	116	23	?	51	VT1, VT2
Blain et al. [98]	2005	Assessment of ventilatory thresholds during graded and maximal exercise test using time varying analysis of respiratory sinus arrhythmia	14	25	23	45	VT1, VT2
Brunetto et al. [99]	2005	Ventilatory threshold and heart rate variability in adolescents	41	15	21	41	VT2
Buchheit et al. [100]	2007	Heart-rate deflection point and the second heart-rate variability threshold during running exercise in trained boys	72	13	18	54	VT2
Cassirame et al. [101]	2015	Heart rate variability to assess ventilatory threshold in ski-mountaineering	9	32	21	67	VT2
Cottin et al. [37]	2006	Assessment of ventilatory thresholds from heart rate variability in well-trained subjects during cycling	11	20	21	68	VT1, VT2
Cottin et al. [102]	2007	Ventilatory thresholds assessment from heart rate variability during an incremental exhaustive running test	12	25	24	53	VT1, VT2
Cunha et al. [103]	2014	Influence of exercise modality on agreement between gas exchange and heart rate variability thresholds	16	21	25	42	VT2
Di Michele et al. [31]	2012	Estimation of the anaerobic threshold from heart rate variability in an incremental swimming test	14	17	22	?	LT2
Dourado et al. [32]	2010	A simple approach to assess VT during a field walk test	10	56	26	26	VT2
Fenzl et al. [104]	2013	High power spectral density of heart rate variability as a measure of exercise performance in water	12	36	23	49	VT2
Flöter et al. [105]	2012	Assessment of the individual anaerobic threshold from heart rate variability in interdependency to the activity of the sympathetic activation	20	27	23	48	LT2
García-Manso et al. [106]	2008	Wavelet transform analysis of heart rate variability for determining ventilatory thresholds in cyclists	8	17	23	79	VT1, VT2
Garcia-Tabar et al. [107]	2013	Heart rate variability thresholds predict lactate thresholds in professional world-class road cyclists	12	27	22	?	LT1, LT2
Grannell et De Vito [108]	2018	An investigation into the relationship between heart rate variability and the ventilatory threshold in healthy moderately trained males	10	27	25	49	VT2
Hamdan et al. [109]	2016	Determining cardiac vagal threshold from short term heart rate complexity	19	24	24	45	LT1, LT2
Hargens et al. [110]	2022	Reliability of the heart rate variability threshold during treadmill exercise	10	21	26	54	VT2
Karapetian [111]	2008	Heart rate variability as a non-invasive biomarker of sympatho-vagal interaction and determinant of physiologic thresholds	53	56	33	10	VT2 LT2
Karapetian et al. [112]	2008	Use of heart rate variability to estimate LT and VT	28	25	24	32	VT2 LT2
Leprêtre [113]	2013	Determination of ventilatory threshold using heart rate variability in patients with heart failure	18	62	27	18	VT1
López-Fuenzalida et al. [114]	2016	Estimation of the Aerobic-anaerobic Transition by Heart Rate Variability in Athletes and Non-athletes Subjects	24	22	25	?	VT2
Mateo-March et al. [115]	2022	Validity of detrended fluctuation analysis of heart rate variability to determine intensity thresholds in elite cyclists	38	23	21	71	LT1, LT2
Mina-Paz et al. [116]	2021	Ventilatory Threshold Concordance Between Ergoespirometry and Heart Rate Variability in Female Professional Cyclists	12	24	21	56	VT1, VT2
Mourot et al. [117]	2012	Heart rate variability to assess ventilatory thresholds: reliable in cardiac disease?	14	58	26	23	VT1, VT2
Mourot et al. [118]	2014	Second ventilatory threshold from heart-rate variability: valid when the upper body is involved?	16	25	22	59	VT2
Nascimento et al. [119]	2017	Determination of lactate thresholds in maximal running test by heart rate variability data set	19	30	24	?	LT1, LT2
Nascimento et al. [120]	2019	Applicability of Dmax Method on Heart Rate Variability to Estimate the Lactate Thresholds in Male Runners	19	30	24	?	LT1, LT2
Neves et al. [20]	2022	Is There Agreement and Precision between Heart Rate Variability, Ventilatory, and Lactate Thresholds in Healthy Adults?	34	22	24	48	VT1, VT2 LT1, LT2
Queiroz et al. [121]	2018	Heart rate variability estimates ventilatory threshold regardless body mass index in young people	10	22	23	37	VT2
Quinart et al. [122]	2014	Ventilatory thresholds determined from HRV: comparison of 2 methods in obese adolescents	20	14	33	28	VT1, VT2
Ramos-Campo et al. [123]	2017	Heart rate variability to assess ventilatory thresholds in professional basketball players	24	23	24	52	VT1, VT2
Rogers et al. [49]	2021	A New Detection Method Defining the Aerobic Threshold for Endurance Exercise and Training Prescription Based on Fractal Correlation Properties of Heart Rate Variability	17	29	25	56	VT1
Rogers et al. [124]	2021	Aerobic Threshold Identification in a Cardiac Disease Population Based on Correlation Properties of Heart Rate Variability	16	55	26	29	VT1
Rogers et al. [125]	2021	Detection of the Anaerobic Threshold in Endurance Sports: Validation of a New Method Using Correlation Properties of Heart Rate Variability	17	27	25	56	VT2
Rogers et al. [48]	2022	An Index of Non-Linear HRV as a Proxy of the Aerobic Threshold Based on Blood Lactate Concentration in Elite Triathletes	9	24	23	67	LT1
Rogers et al. [126]	2023	Improved Estimation of Exercise Intensity Thresholds by Combining Dual Non-Invasive Biomarker Concepts: Correlation Properties of Heart Rate Variability and Respiratory Frequency	21	40	24	41	VT1, VT2
Sales et al. [127]	2011	Non-invasive method to estimate anaerobic threshold in individuals with type 2 diabetes	19	55	34	22	VT2 LT2
Schaffarczyk et al. [128]	2023	Validation of a non-linear index of heart rate variability to determine aerobic and anaerobic thresholds during incremental cycling exercise in women	31	32	24	36	VT1, VT2
Shiraishi et al. [129]	2018	Real-Time Analysis of the Heart Rate Variability During Incremental Exercise for the Detection of the Ventilatory Threshold	65	45	23	32	VT2 LT2
Simões et al. [130]	2010	Heart-rate variability and blood-lactate threshold interaction during progressive resistance exercise in healthy older men	15	64	25	30	LT2
Simões et al. [131]	2013	Lactate and heart rate variability threshold during resistance exercise in the young and elderly	14	23	25	36	LT2
Simões et al. [132]	2014	Identification of anaerobic threshold by analysis of heart rate variability during discontinuous dynamic and resistance exercise protocols in healthy older men	20	70	26	24	LT2
Simões et al. [133]	2016	Use of Heart Rate Variability to Estimate Lactate Threshold in Coronary Artery Disease Patients during Resistance Exercise	10	64	25	?	LT1
Sperling et al. [134]	2016	Is heart rate variability a feasible method to determine anaerobic threshold in progressive resistance exercise in coronary artery disease?	21	63	27	24	LT2
Stergiopoulos et al. [135]	2021	Second Ventilatory Threshold Assessed by Heart Rate Variability in a Multiple Shuttle Run Test	17	22	24	55	VT2
Thiart et al. [136]	2023	Heart Rate Variability-Established Thresholds to Determine the Ventilatory and Lactate Thresholds of Endurance Athletes	21	24	22	71	VT1, VT2 LT2
Tschanz et al. [137]	2020	Determination of the ventilatory thresholds by the heart rate variability	35	24	22	52	VT1, VT2
Vasconcellos et al. [138]	2015	Can Heart Rate Variability be used to Estimate Gas Exchange Threshold in Obese Adolescents?	35	15	30	27	VT2
Zimatore et al. [139]	2020	Recurrence quantification analysis of heart rate variability during continuous incremental exercise test in obese subjects	20	42	39	?	VT2

## Results

**Fig. 1:**
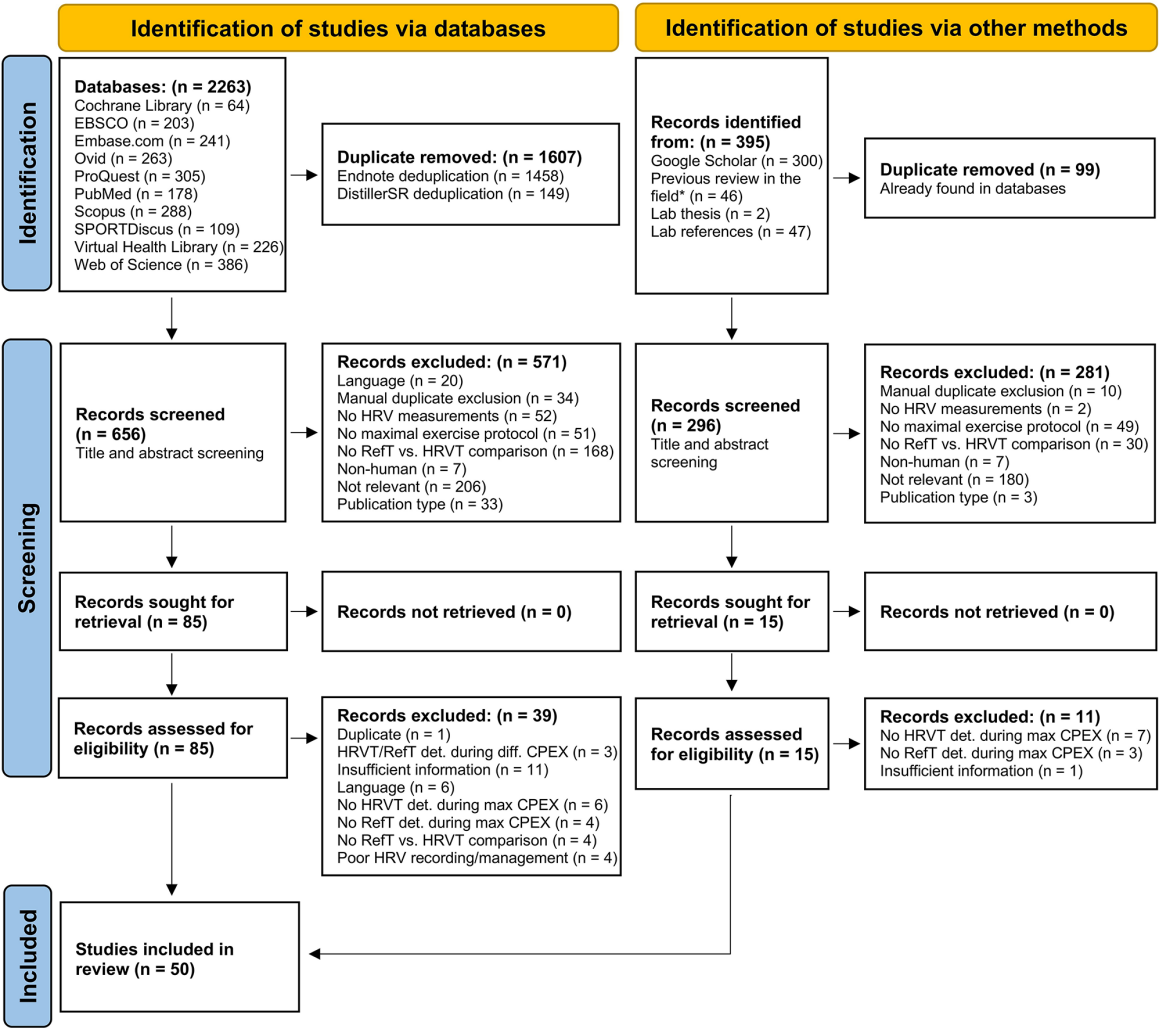
PRISMA flow diagram of the systematic review process showing identified, included, and excluded studies. n, number of studies. *Gomes and Molina [54], Zimatore et al. [53], Kaufmann et al. [52]

After removing duplicates, our search strategy identified 952 original records for screening. Of these, 852 were excluded during the title and abstract screening and 50 during full-text review. Finally, 50 studies [20, 31, 32, 37, 46, 48, 49, 97–139] fulfilled the inclusion criteria detailed above and were included in this systematic review with meta-analyses. The summary of the screening process is presented as a PRISMA flow diagram in Fig. 1. The agreements between HRVT1 – and LT1-VT1, and between HRVT2 – LT2-VT2 were assessed in 22 [20, 37, 48, 49, 98, 102, 106, 107, 109, 113, 115–117, 119, 120, 122, 123, 126, 128, 133, 136, 137] and 42 [20, 31, 32, 37, 46, 98–107, 109–111, 114–123, 125–132, 134–139] studies, respectively; the corresponding correlations were assessed in 22 [37, 48, 49, 97, 98, 102, 107, 109, 113, 115–117, 119, 120, 122–124, 126, 128, 133, 136, 137] and 41 [31, 32, 37, 46, 97–105, 107–109, 111, 112, 114–123, 125–132, 135–139] studies respectively. Across all 50 studies, 314 distinct agreement assessments (95 for HRVT1 and 219 for HRVT2) and 246 distinct correlation assessments (82 for HRVT1 and 164 for HRVT2) between LT-VTs and HRVTs were analysed. Overall, data from 1160 different subjects (on average 23 per study, range 8–116; age 32 (13–70) years, BMI 25 (18–39) kg/m^2^, V̇O_2_max 44 (10–79) mL⋅kg^–1^⋅min^–1^) were included. The characteristics of each study are presented in Table 1.

### Methodological Quality Assessment

**Fig. 2:**
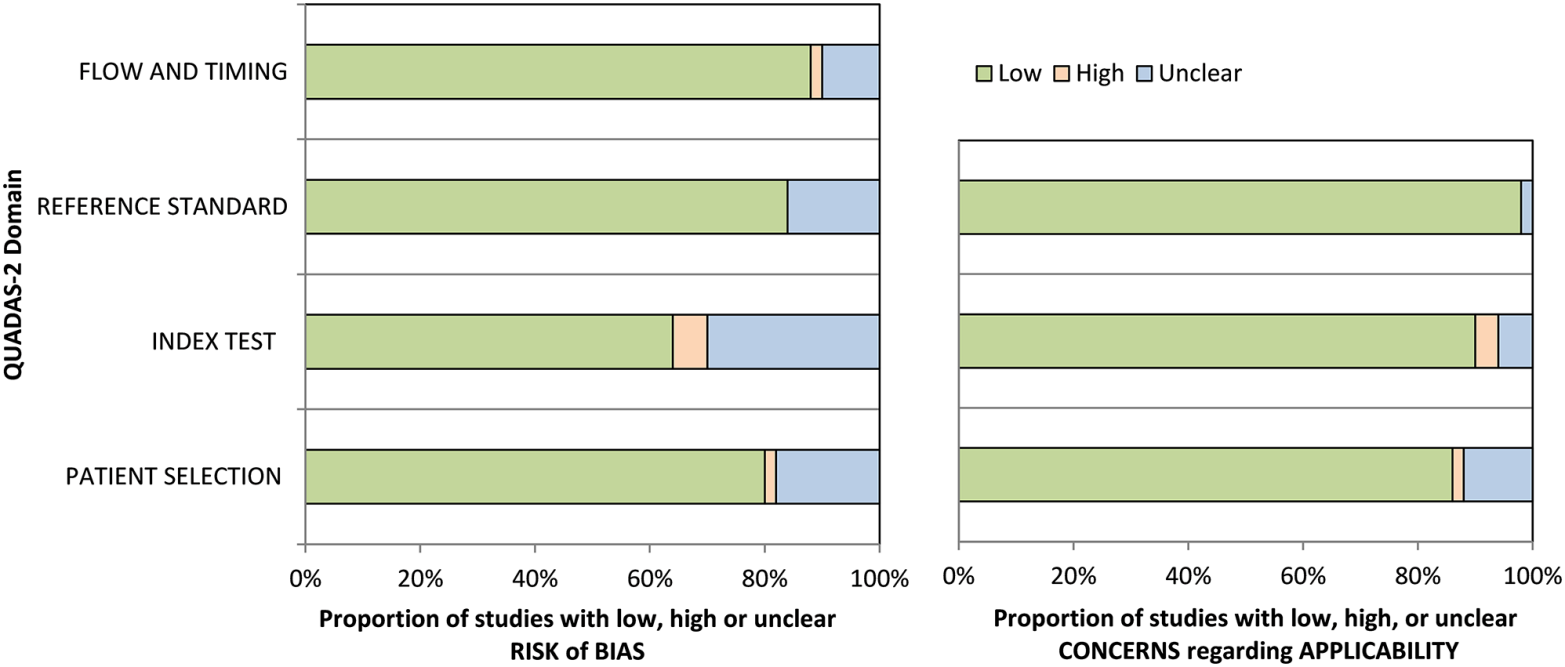
Risk of bias and applicability of included studies assessed by QUADAS-2

The risk of bias was assessed as “low” in the four QUADAS-2 domains for 21 of the 50 included studies, and four studies were assessed as “high” in at least one RoB domain. The remaining 24 studies were assessed as having an “unclear” RoB in one or more domains. The concern regarding applicability was assessed as “low” in the three QUADAS-2 domains for 40 of the 50 included studies. Two studies were assessed as “high” in at least one domain for applicability concerns. The remaining eight studies were assessed as having “unclear” concerns regarding applicability in one or more domains. QUADAS-2 overall assessment is shown in Fig. 2. Detailed RoB assessment by QUADAS-2 for each included study is presented in Online Resource 3.

Methodological quality assessment using the adapted STARD_HRV_ [52] for the 50 included studies reached an average score of 78 ± 8% (range 62 – 94%). Three studies reached ≥ 90%, 22 reached between 80% and 89%, 15 reached between 70% and 79%, and 10 reached < 70%. Nearly all studies were identified as a validation study (item 1, 100%), had a structured abstract (item 2, 98%), described scientific and practical background (item 3, 100%), used a within-subject design (item 5, 100%), described the setup for LT-VT and HRVT extensively (item 9, 100%), described how comparison calculations were performed (item 14, 98%), provided baseline demographics of participants (item 20, 100%) and full study protocol (item 24, 100%). Only a few studies provided information about sample size determination (16%), mentioned a stabilisation period prior to HRV sampling (40%), and specified whether breathing was controlled or not during HRV recording (30%). All other items were fulfilled by 53–93% of included studies. Details of the STARD_HRV_ assessment for each study are presented in Online Resource 4.

### First Heart Rate Variability vs. Lactate and Ventilatory Thresholds

**Fig. 3:**
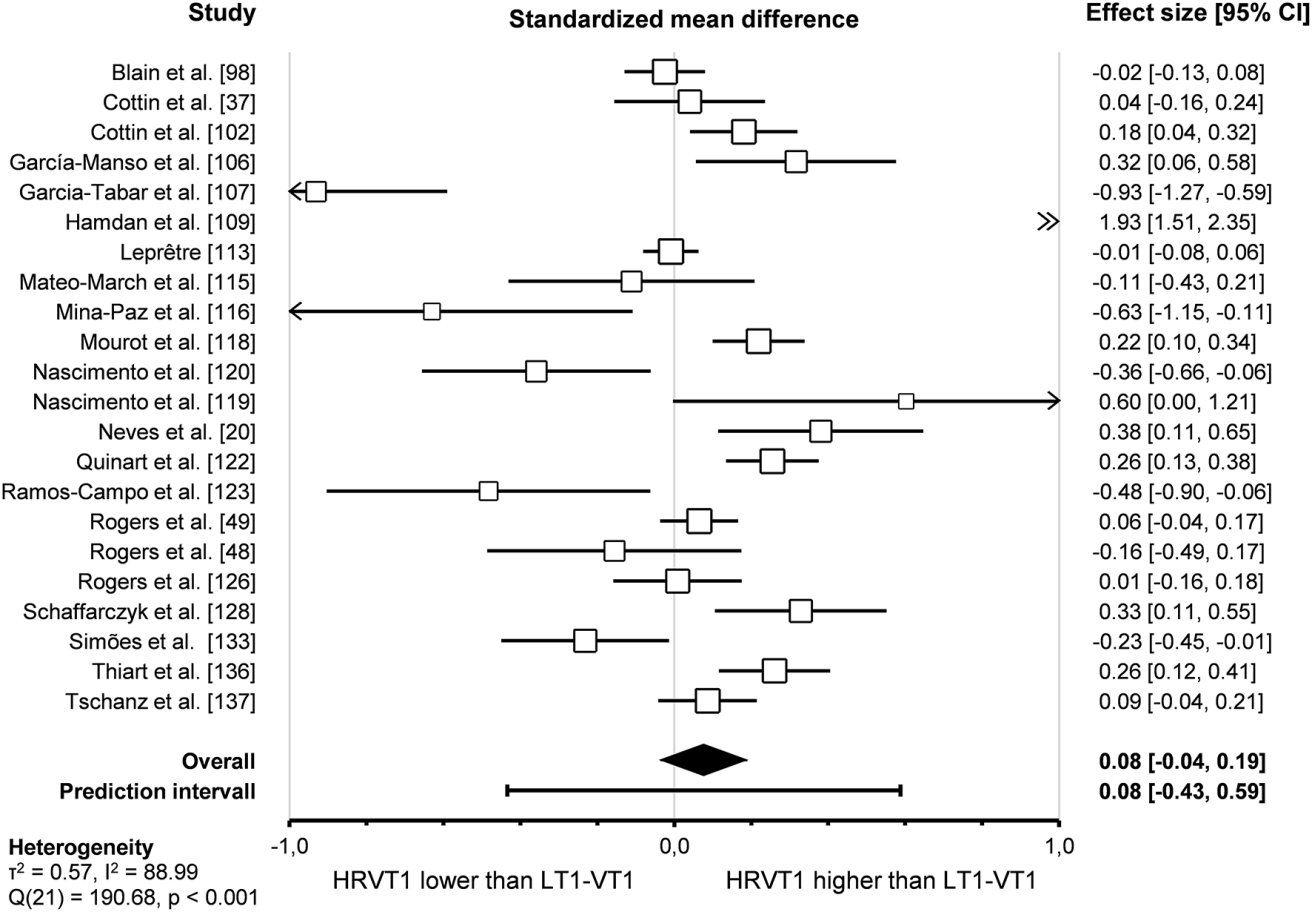
Forest plot of standardised mean difference between HRVT1 and LT1-VT1 (random-effect model)

Pooled analysis of the 22 included studies assessing agreement between HRVT1 and LT1-VT1 revealed a trivial standardised mean difference (SMD = 0.08, 95% CI -0.04–0.19, *p* = 0.18). The prediction interval ranged from − 0.43 to 0.59, indicating that the true effect size falls within this interval in 95% of all comparable studies. The overall effect was heterogeneous (*p* < 0.001), indicating that the true effect size was not the same in those 22 studies. Furthermore, the I^2^ statistic indicates that 89% of the variance in observed effects reflects variance in true effects rather than sampling error. The corresponding forest plot is shown in Fig. 3.

**Fig. 4:**
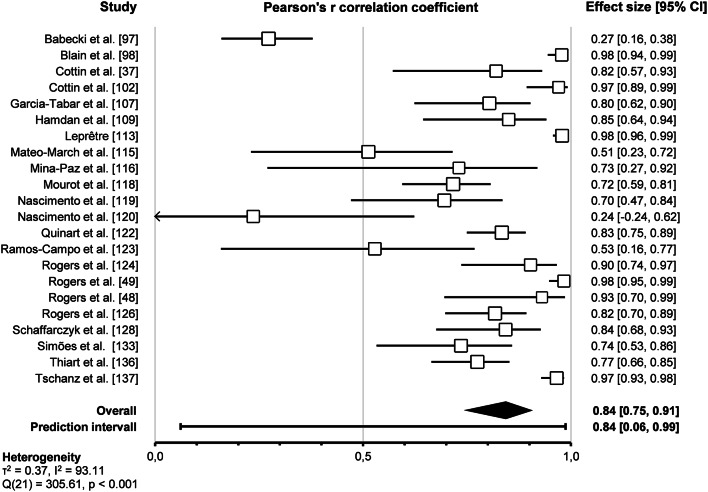
Forest plot of Pearson’s r correlation coefficient between HRVT1 and LT1-VT1 (random-effect model)

Pooled analysis of the 22 included studies assessing the correlation between HRVT1 and LT1-VT1 revealed a very strong correlation (Pearson’s *r* = 0.84, 95% CI 0.75–0.91, *p* < 0.001). The prediction interval ranged from 0.06 to 0.99, indicating that the true effect size falls within this interval in 95% of all comparable studies. The overall effect was heterogeneous (*p* < 0.001), indicating that the true effect size was not the same in those 22 studies. Furthermore, the I^2^ statistic indicates that 93% of the variance in observed effects reflects variance in true effects rather than sampling error. The corresponding forest plot is shown in Fig. 4.

The observed heterogeneity in our HRVT1 primary analyses is high [140] indicating that the results of the included studies diverge from each other. Overall, this makes it more challenging to draw definitive conclusions about combined effect sizes and poses challenges for the interpretation [140–142]. Consequently, we used a random-effects model, which takes into account the heterogeneity between the included studies [78]. Heterogeneity can stem from differences in study participants, interventions, outcomes or study designs [55]. In this context, the determination of the causes of heterogeneity requires subgroup analyses and meta-regression, as presented below, and can provide valuable insights and thereby enhance the overall understanding of HRVT1 determination.

### Moderator Analyses for First Heart Rate Variability Threshold

Since agreement and correlation meta-analyses between HRVT1 and LT1-VT1 showed significantly heterogeneous effects with 89% and 93% of the observed variance due to variance in true effects, subgroup analyses were performed. Pre-specified moderator variables were analysed separately to determine their influence on the agreement (SMD) and the correlation (Pearson’s r) between HRVT1 and LT1-VT1. A forest plot representation corresponding to each HRVT1 subgroup analysis, the subgroup’s heterogeneity assessment, and pairwise comparison p-value between subgroups (if the statistical test for interaction was significant) can be found in Online Resource 5.

#### Subjects’ Characteristics

**Fig. 5:**
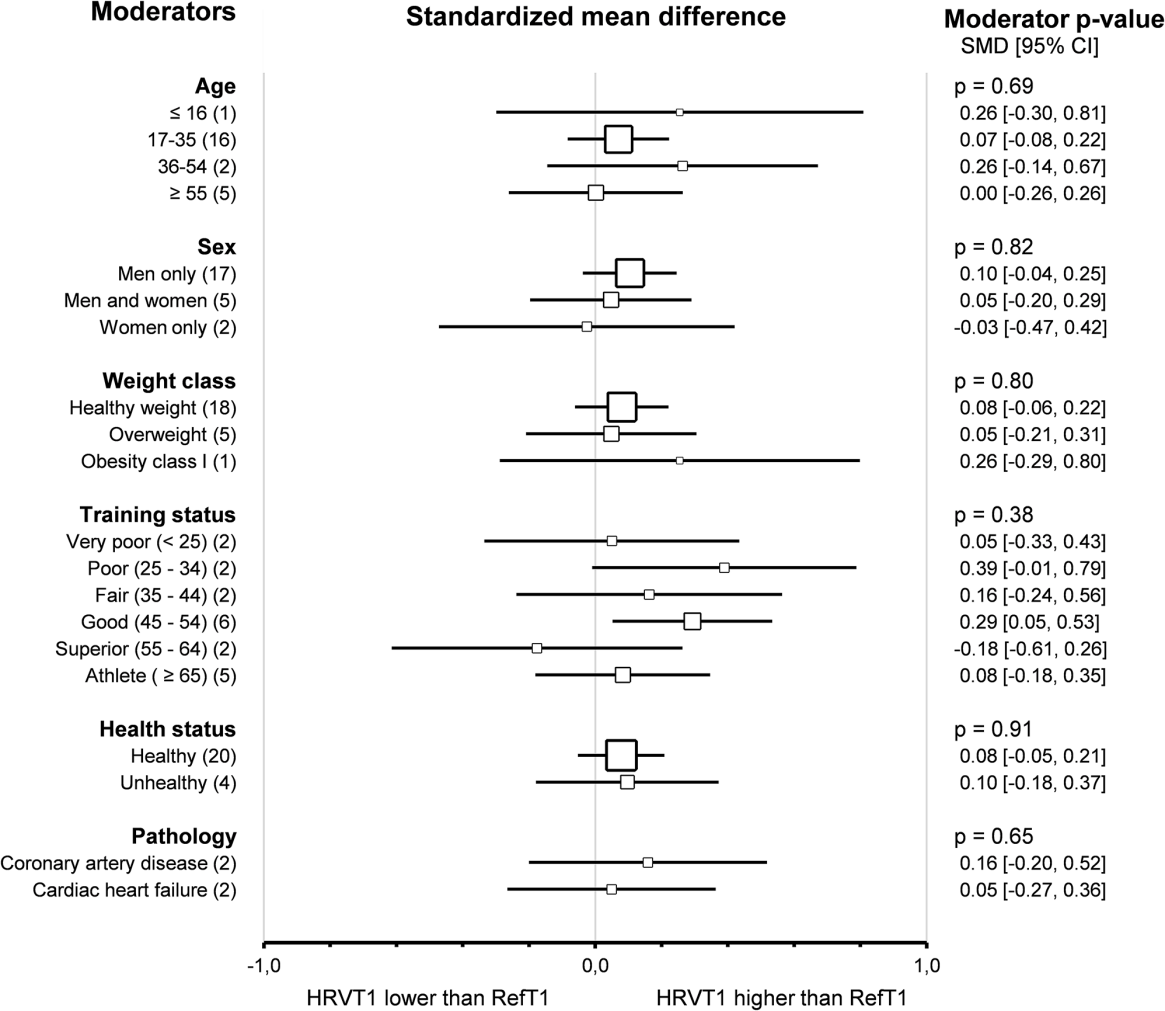
Forest Plots of agreement between HRVT1 and LT1-VT1 with subjects’ characteristics as moderators. Square sizes are proportional to the number of studies in subgroup. CAD, coronary artery disease; CHF, chronic heart failure; number of studies. Training status was classified according to V̇O_2_max (mL · min^-1^ · kg^-1^) as Very poor (< 25), Poor (25–34), Fair (35–44), Good (45–54), Superior (55–64), or Athlete (≥ 65). Weight class was classified according to BMI (kg/m^2^) as Healthy weight (18.5–24), Overweight (25–29), or Obesity class I (30–34)

**Fig. 6:**
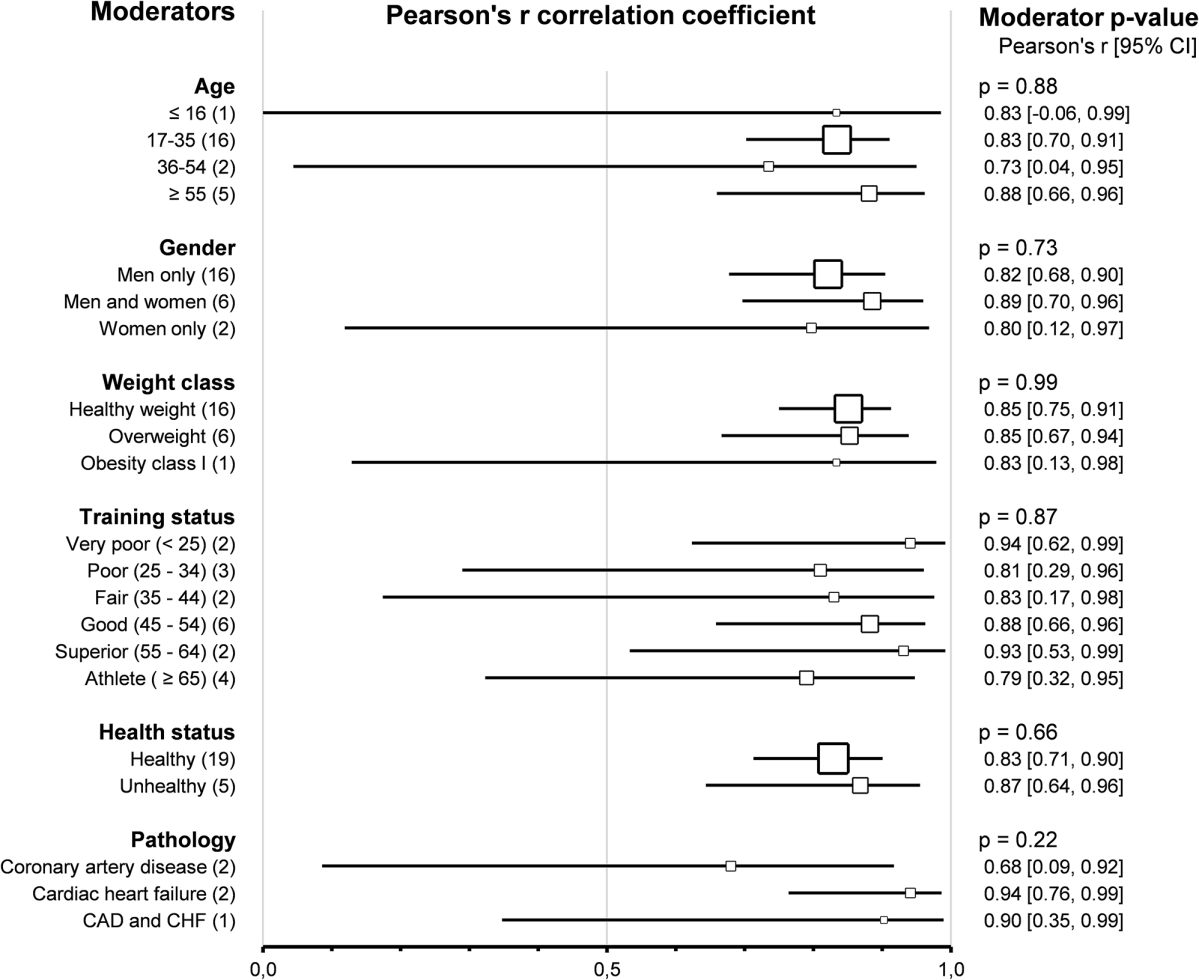
Forest Plots of correlation between HRVT1 and LT1-VT1 with subjects’ characteristics as moderators. Square sizes are proportional to the number of studies in subgroup. CAD, coronary artery disease; CHF, chronic heart failure; n, number of studies. Training status was classified according to V̇O_2_max (mL · min^-1^ · kg^-1^) as Very poor (< 25), Poor (25–34), Fair (35–44), Good (45–54), Superior (55–64), or Athlete (≥ 65). Weight class was classified according to BMI (kg/m^2^) as Healthy weight (18.5–24), Overweight (25–29), or Obesity class I (30–34)

Subgroup comparison analyses for subjects’ characteristics revealed that the agreement and correlation between HRVT1 and LT1-VT1 were not impacted by *age group* (*p* = 0.68 and *p* = 0.88 respectively), *sex* (*p* = 0.82 and *p* = 0.73 respectively), *weight class* (*p* = 0.80 and *p* = 0.99 respectively) and *training status* assessed by V̇O_2_max (*p* = 0.38 and *p* = 0.87 respectively). All these subgroup analyses were confirmed using meta-regressions on the corresponding continuous variable (age, % of men included weight, and V̇O_2_max), which showed no correlation between the subjects’ characteristics and the corresponding effect size (SMD and Person’s r). The subjects’ *health status* did not impact the agreement and correlation between HRVT1 and LT1-VT1 (*p* = 0.91 and *p* = 0.66, respectively). Furthermore, the *pathology* (coronary artery disease [117, 133] vs. cardiac heart failure [13, 117]) affecting the patients included in this meta-analysis also showed no impact on the SMD and Pearson’s r between HRVT1 and LT1-VT1 (*p* = 0.65 and *p* = 0.22, respectively). Overall, none of the subjects’ characteristics impacted either the agreement or the correlation between HRVT1 and LT1-VT1. Details of subjects’ characteristics subgroup analyses are shown as forest plots in Figs. 5 and 6.

#### First Threshold Determination Methods

**Fig. 7:**
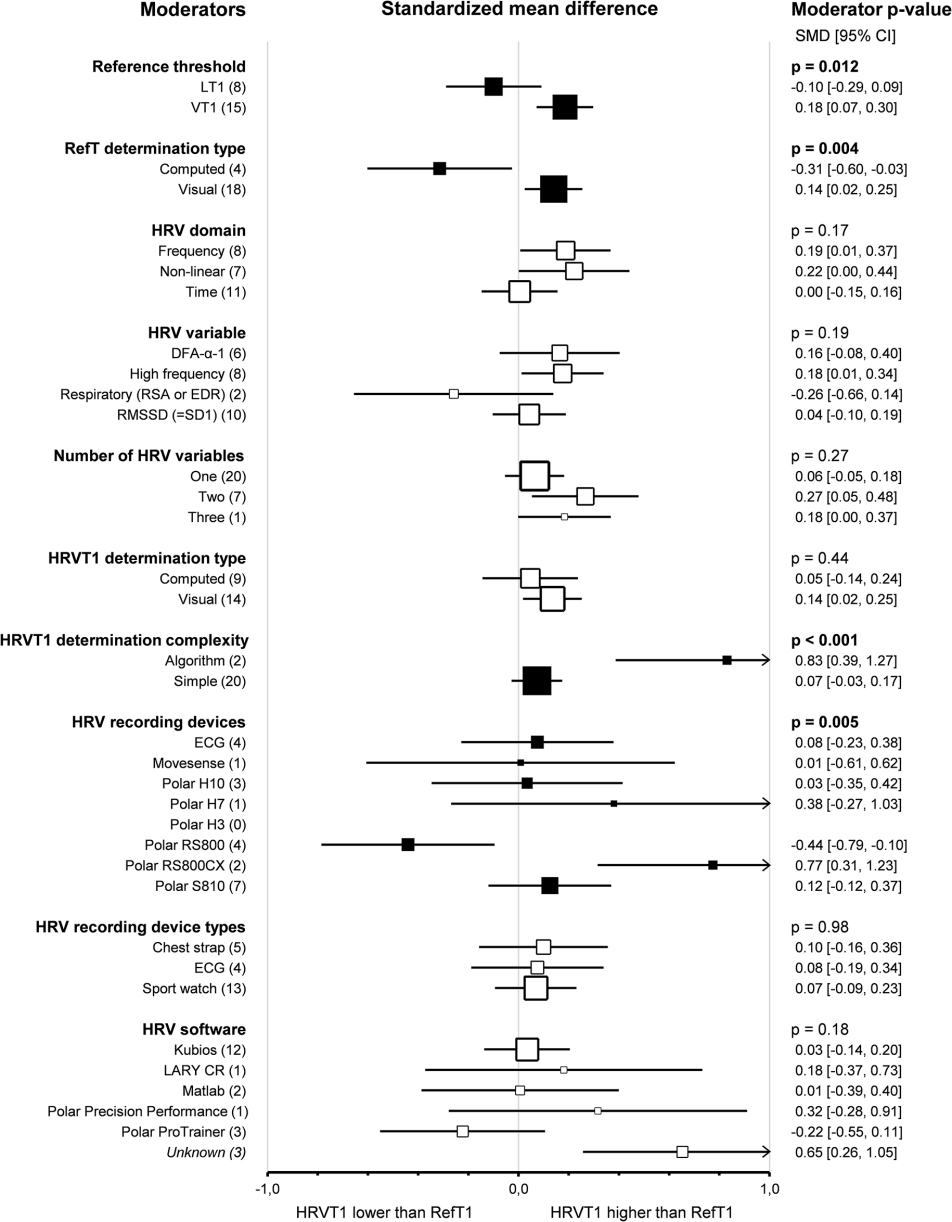
Forest Plots of agreement between HRVT1 and LT1-VT1 with thresholds determination characteristics as moderators. Solid black squares indicate moderators with a significant impact on effect size. Square sizes are proportional to the number of studies in subgroup. DFA-ɑ1, detrended fluctuation analysis alpha 1; ECG, electrocardiogram; EDR, ECG derived respiration; HRVT1, heart rate variability threshold 1; n, number of studies; LT1-VT1, reference threshold 1; RMSSD, root mean square of successive differences; RSA, respiratory sinus arrhythmia; SD1, Poincaré plot standard deviation

**Fig. 8:**
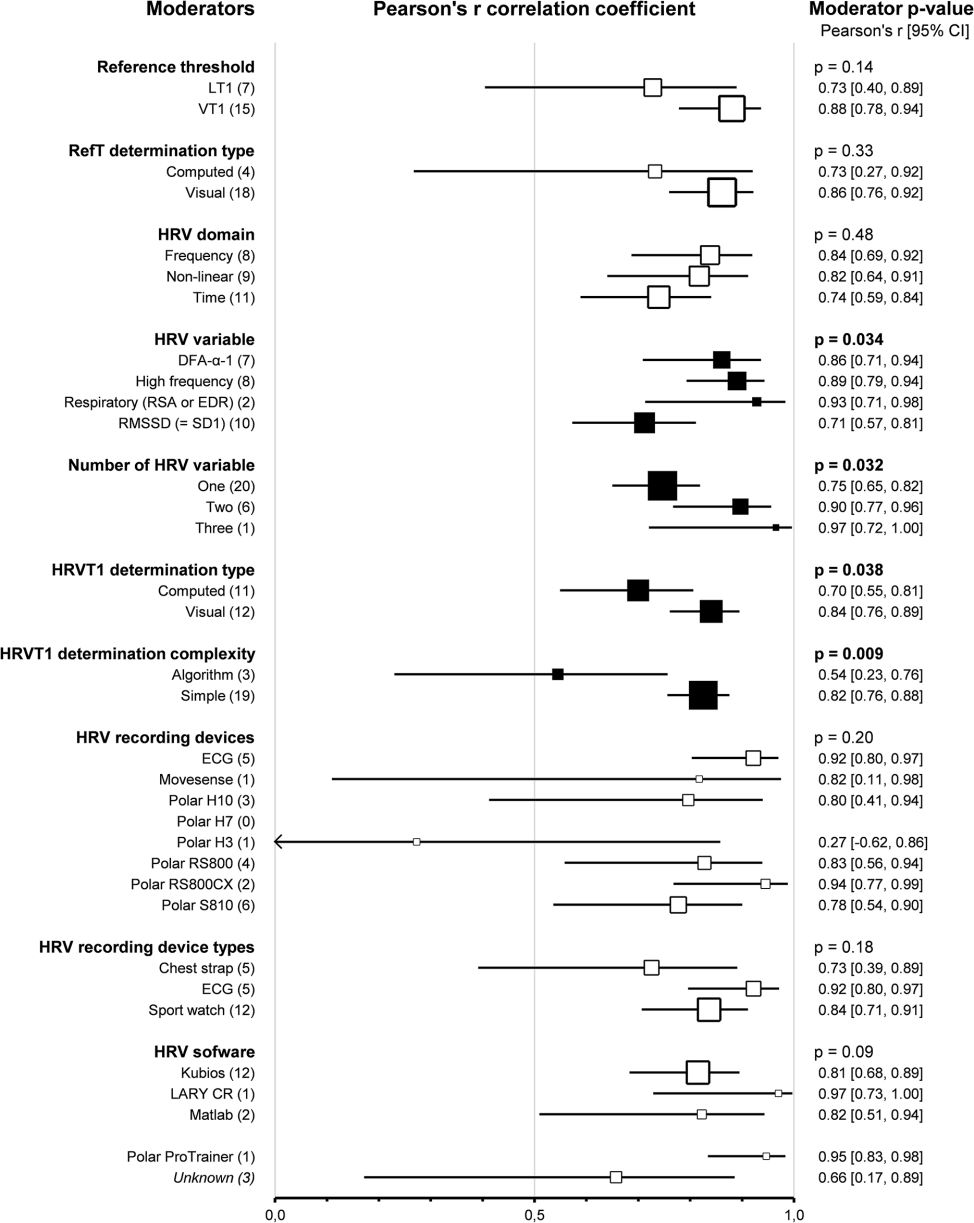
Forest Plots of correlation between HRVT1 and LT1-VT1 with thresholds determination characteristics as moderators. Solid black squares indicate moderators with a significant impact on effect size. Square sizes are proportional to the number of studies in subgroup. DFA-ɑ1, detrended fluctuation analysis alpha 1; ECG, electrocardiogram; EDR, ECG derived respiration; HRVT1, heart rate variability threshold 1; n, number of studies; LT1-VT1, reference threshold 1; RMSSD, root mean square of successive differences; RSA, respiratory sinus arrhythmia; SD1, Poincaré plot standard deviation

Subgroup comparison analyses for HRV and LT-VT methods revealed that *reference thresholds* impacted the agreement between HRVT1 and LT1-VT1 (*p* = 0.01). Indeed, HRVT1 was higher when compared to VT (0.18, 0.07–0.30, *n* = 15) than when compared to LT (-0.10, -0.29–0.09, *n* = 8, *p* = 0.01). Furthermore, when VTs were used as a reference for HRVT1 determination, there was a difference in agreement between VT1 and HRVT1 (*p* = 0.001). The reference threshold did not impact the correlation between HRVT1 and LT1-VT1 (*p* = 0.14). *Reference threshold determination type* also impacted the agreement between HRVT1 and LT1-VT1. Indeed, HRVT1 was higher when the LT-VT was determined visually (0.14, 0.02–0.25, *n* = 18) than when computed (-0.31, -0.60 – -0.03, *n* = 4, *p* = 0.004). The reference threshold determination type did not impact the correlation between HRVT1 and LT1-VT1 (*p* = 0.33). *HRV domains* used to determine HRVT1 did not influence the agreement between HRVT1 and LT1-VT1 (*p* = 0.17). However, when HRVT1 was determined by Frequency (0.19, 0.01–0.37, *n* = 8) or by Non-linear domain (0.22, 0.00–0.44, *n* = 7), there was a difference in SMD between HRVT1 and LT1-VT1 (*p* = 0.041 and *p* = 0.048 respectively). Time domain variables (0.00, -0.15, 0.16, *n* = 11) showed the best agreement between HRVT1 and LT1-VT1. *HRV variables* used to determine HRVT1 did not impact the agreement between HRVT1 and LT1-VT1 (*p* = 0.19). The RMSSD was the most precise HRV variable used for HRVT1 determination (0.04, -0.10–0.19, *n* = 10), followed by DFA-α1 (0.16, -0.08–0.40, *n* = 6), and Respiratory-derived HRV thresholds (using respiratory sinus arrhythmia or ECG derived respiration) (-0.26, -0.66–0.14, *n* = 2). HF-derived HRVT1 were higher than LT1-VT1 (0.18, 0.01–0.34, *n* = 8, *p* = 0.03). The HRV variable also impacted the correlation between HRVT1 and LT1-VT1 (*p* < 0.001). Pearson’s r was higher with HF (0.89, 0.79–0.98, *n* = 8) than with RMSSD-derived thresholds (0.71, 0.57–0.81, *n* = 10, *p* = 0.01). DFA-α1 derived HRVT1 (0.86, 0.71–0.94, *n* = 7) and Respiratory-derived HRVT (0.93, 0.71–0.98, *n* = 2) both showed very strong correlation with LT1-VT1. HRV variables used only for one HRVT1 determination were not included in this subgroup analysis for reasons of clarity and robustness. The *number of HRV variables* used to determine each HRVT1 had no impact on the agreement between HRVT1 and LT1-VT1 (*p* = 0.27). The HRVT1s determined with a combination of Two (0.27, 0.05–0.48, *n* = 7 [37, 102, 106, 117, 122, 126, 136]) or Three (0.18, 0.01–0.37, *n* = 1 [137]) HRV variables were not more precise than with One HRV variable (0.06, -0.05–0.18, *n* = 20). Furthermore, when Two HRV variables were combined, the HRVT1 was higher than LT1-VT1 (*p* = 0.01). The number of HRV variables used to determine HRVT1 impacted the correlation between HRVT1 and LT1-VT1 (*p* = 0.03). Indeed, when Two HRV variables were combined (0.90, 0.77–0.96, *n* = 6 [37, 102, 117, 122, 126, 136]), Pearson’s r was higher than with One (0.75, 0.65–0.82, *n* = 20, *p* = 0.046). The study using Three HRV variables [137] showed a 0.97 (0.72–0.99) correlation between HRVT1 and LT1-VT1. The *HRVT1 determination type* (whether computed or visually determined) did not impact the agreement between HRVT1 and LT1-VT1. However, the determination type impacted the correlation between HRVT1 and LT1-VT1 (*p* = 0.04). Indeed, the visual determination of HRVT1 (0.84, 0.76–0.89, *n* = 12) showed a stronger correlation with LT1-VT1 than the computed determination (0.70, 0.55–0.81, *n* = 11, *p* = 0.04). The *HRVT1 determination complexity* had an impact on both agreement (*p* < 0.001) and correlation (*p* = 0.01) between HRVT1 and LT1-VT1. Indeed, with Simple HRVT1 determination, agreement was better (0.07, -0.03–0.17, *n* = 20) and correlation stronger (0.82, 0.76–0.88, *n* = 19) than with algorithmic HRVT determination (SMD: 0.83, 0.39–1.27, *n* = 2 [109, 137]; Pearson’s r: 0.54, 0.23–0.76, *n* = 3 [97, 109, 137]). *HRV recording devices* impacted the agreement between HRVT1 and LT1-VT1 (*p* = 0.01). HRVT1 determined using a Polar RS800 (-0.44, -0.79 – -0.10, *n* = 4) were lower than those obtained with ECG (0.08, -0.23–0.38, *n* = 4, *p* = 0.03), PolarH7 (0.38, -0.27–0.1.03, *n* = 1, *p* = 0.03), PolarRS800CX (0.77, 0.31–1.23, *n* = 2, *p* = 0.01) and PolarS810 (0.12, -0.12–0.37, *n* = 7, *p* < 0.001). HRVT1 determined using a Polar RS800CX were higher than those determined using a Polar S810 (*p* = 0.01). The HRVT recording device did not impact the correlation between HRVT1 and LT1-VT1 (*p* = 0.20). *HRV recording device type* (whether chest strap, ECG or sport watch was used) had no impact on the agreement and correlation between HRVT1 and LT1-VT1 (*p* = 0.98 and *p* = 0.18, respectively). Furthermore, none of the recording device types highlighted a difference in agreement between HRVT1 and LT1-VT1: Chest strap (0.10, -0.16–0.36, *n* = 5), ECG (0.08, -0.19–0.34, *n* = 4), sport watch (0.07, -0.09–0.23, *n* = 13). *HRV software* impacted the agreement between HRVT1 and LT1-VT1 (*p* = 0.03). Indeed, the HRVT1 was statistically higher than LT1-VT1 when the software was not mentioned in the study (0.65, 0.26–1.05, *n* = 3). When the software was not specified, the HRVT1 was also higher than when Kubios (0.03, -0.14–0.20, *n* = 12, *p* = 0.01), Matlab (0.01, -0.39–0.40, *n* = 2, *p* = 0.02) or Polar ProTrainer (-0.22, -0.55–0.11, *n* = 3, *p* < 0.001) were used. The HRV software did not impact the correlation between HRVT1 and LT1-VT1 (*p* = 0.09). Details of threshold determinations subgroup analyses are shown as forest plots in Figs. 7 and 8, in which solid black squares indicate moderators significantly impacting effect size.

#### Study Protocol

**Fig. 9:**
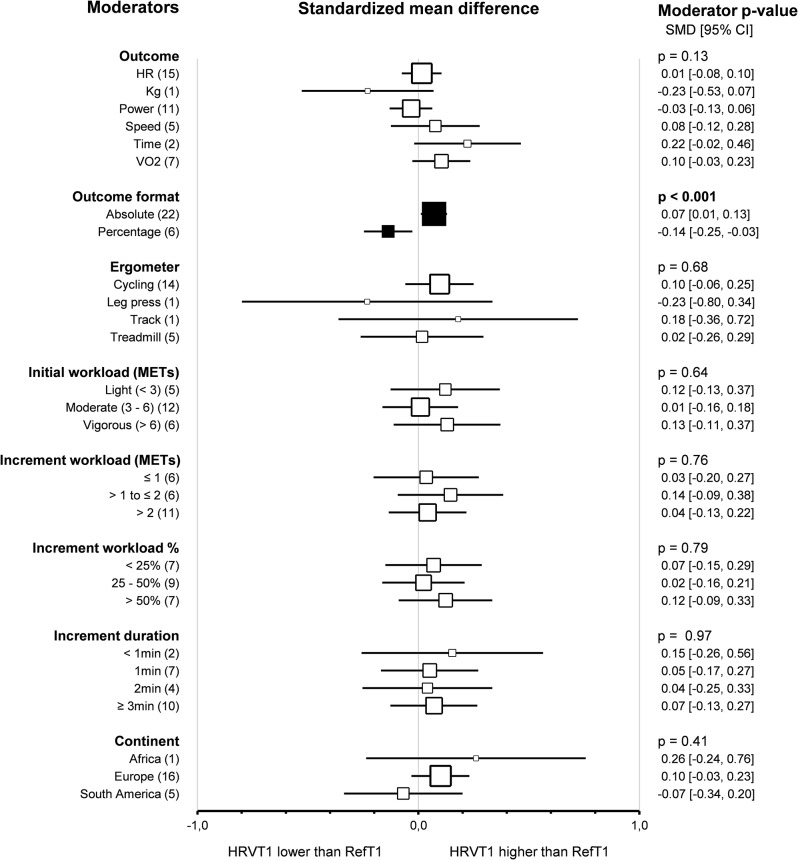
Forest Plots of agreement between HRVT1 and LT1-VT1 with study protocol characteristics as moderators. Solid black squares indicate moderators with significant impact on effect size. Square sizes are proportional to the number of studies in subgroup. n, number of studies. V̇O_2_max, oxygen consumption. Initial workload was classified according to the corresponding METs as Light (< 3), Moderate (3–6), or Vigorous (> 6)

**Fig. 10:**
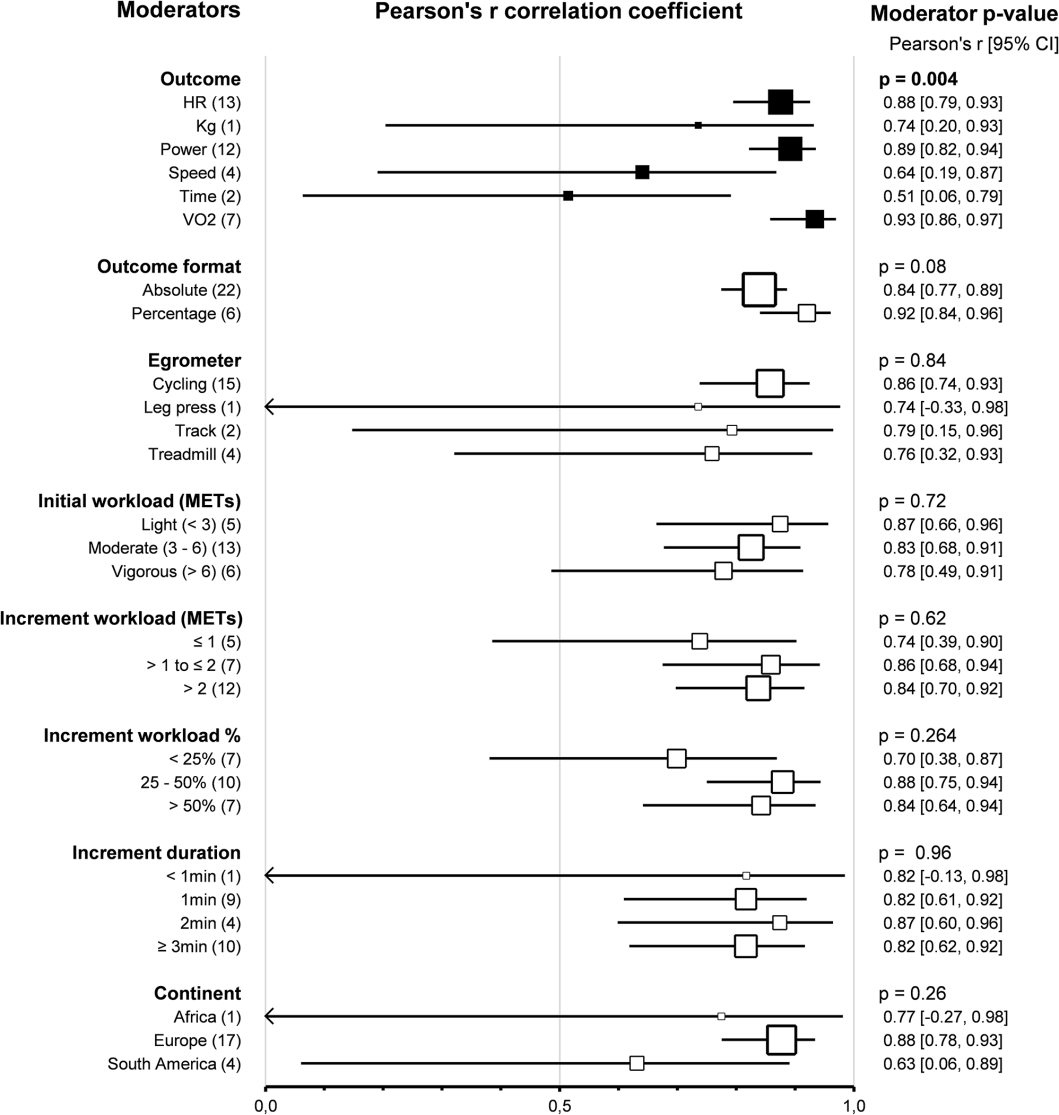
Forest Plots of correlation between HRVT1 and LT1-VT1 with study protocol characteristics as moderators. Solid black squares indicate moderators with significant impact on effect size. Square sizes are proportional to the number of studies in subgroup. n, number of studies. V̇O_2_max, oxygen consumption. Initial workload was classified according to the corresponding METs as Light (< 3), Moderate (3–6), or Vigorous (> 6)

Subgroup comparison analyses for study protocols revealed that the *outcomes* did not impact the agreement between HRVT1 and LT1-VT1 (*p* = 0.13). Furthermore, none of the outcomes used highlighted a difference in agreement between HRVT1 and LT1-VT1: Heart Rate (0.01, -0.08–0.10, *n* = 15), Kg (-0.23, -0.53–0.07, *n* = 1), Power (-0.03, -0.13–0.06, *n* = 11), Speed (0.08, -0.12–0.28, *n* = 5), Time (0.22, -0.02–0.46, *n* = 2), V̇O_2_ (0.10, -0.03–0.23, *n* = 7). However, outcomes impacted the correlation between HRVT1 and LT1-VT1 (*p* = 0.004). Indeed, the correlation was lower for Time (0.51, 0.06–0.79, *n* = 2) than Heart Rate (*r* = 0.88, 0.79–0.93, *n* = 13) (*p* = 0.007), Power (0.89, 0.82–0.94, *n* = 12) and V̇O_2_ (0.93, 0.0.86–0.0.97, *n* = 7). The correlation was also lower for Speed (0.64, 0.19–0.87, *n* = 4) than Power and V̇O_2_. The Pearson’s r for Kg was equal to 0.74 (0.20–0.93, *n* = 1). *Outcome formats* impacted the agreement between HRVT1 and LT1-VT1 (*p* < 0.001). Indeed, when the outcomes mentioned above were expressed as absolute values (0.07, 0.01–0.13, *n* = 22), the HRVT1 was higher than when expressed as a percentage of a maximal value (-014, -0.25 – -0.03, *n* = 6). However, the outcome format had no impact on the correlation between HRVT1 and LT1-VT1 expressed as absolute (0.84, 0.77–0.89, *n* = 22) or percentage (0.92, 0.84–0.96, *n* = 22) values (*p* = 0.08). *Ergometers* used for the incremental exercise test did not impact the agreement and correlation between HRVT1 and LT1-VT1 (*p* = 0.68 and *p* = 0.84, respectively). Furthermore, subgroups analysis showed that *initial workload* in METs (*p* = 0.64, *p* = 0.72), *increment workload* in METs (*p* = 0.75, *p* = 0.62) or in percentage of initial workload (*p* = 0.79, *p* = 0.26) and *increment duration* (*p* = 0.97, *p* = 0.96) had no impact on the agreement and correlation between HRVT1 and LT1-VT1. All these subgroup analyses were confirmed using meta-regressions on the corresponding continuous variables, which showed no correlation between the characteristics of the incremental test protocols and the corresponding effect size (SMD and Person’s r). The *continent* where the study was conducted had no impact on the agreement and correlation between HRVT1 and LT1-VT1 (*p* = 0.41 and *p* = 0.26, respectively). Meta-regression analysis revealed that the *publication date* did not affect the agreement and correlation between HRVT1 and LT1-VT1 (*p* = 0.97 and *p* = 0.13, respectively). Furthermore, meta-regression showed that the SMD and Pearson’s r were unrelated to either the *study sample size* (*p* = 0.22 and *p* = 0.93, respectively) or the *number of comparisons* between HRVT1 and LT1-VT1 done in each study (*p* = 0.39 and *p* = 0.61, respectively). Details of study protocol subgroup analyses as forest plots in Figs. 9 and 10, in which solid black squares indicate moderators significantly impacting effect size.

### Second Heart Rate Variability vs. Lactate and Ventilatory Thresholds

**Fig. 11:**
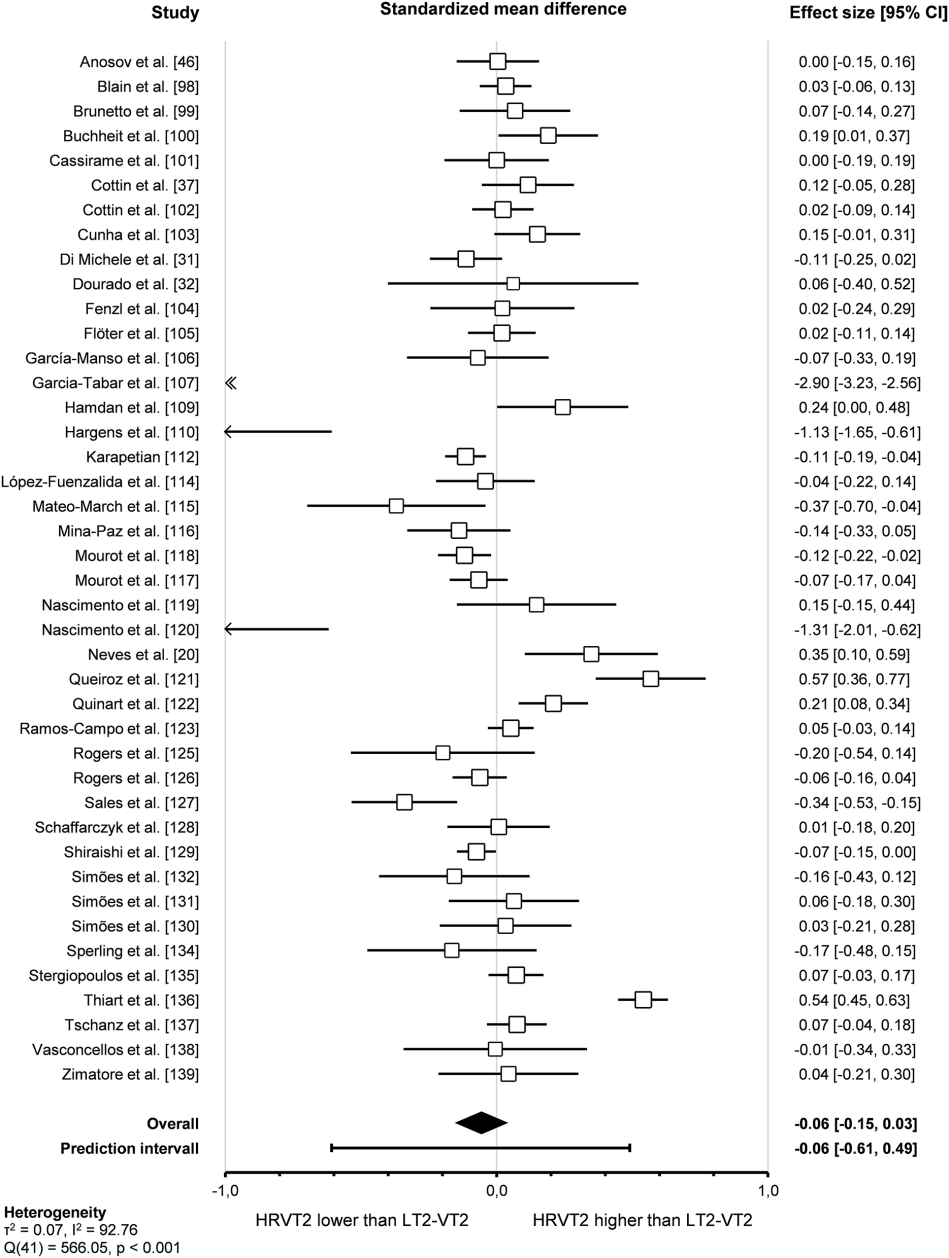
Forest plot of standardised mean difference between HRVT2 and LT2-VT2 (random-effect model)

Pooled analysis of the 42 included studies assessing agreement between HRVT2 and LT2-VT2 revealed a trivial standardised mean difference (SMD = -0.06, 95% CI -0.15–0.03, *p* = 0.19). The prediction interval ranged from − 0.61 to 0.49, indicating that the true effect size falls within this interval in 95% of all comparable studies. The overall effect was heterogeneous (*p* < 0.001), suggesting that the true effect size was not the same in those 42 studies. Furthermore, the I^2^ statistic indicates that 93% of the variance in observed effects reflects variance in true effects rather than sampling error. The corresponding forest plot is shown in Fig. 11.

**Fig. 12:**
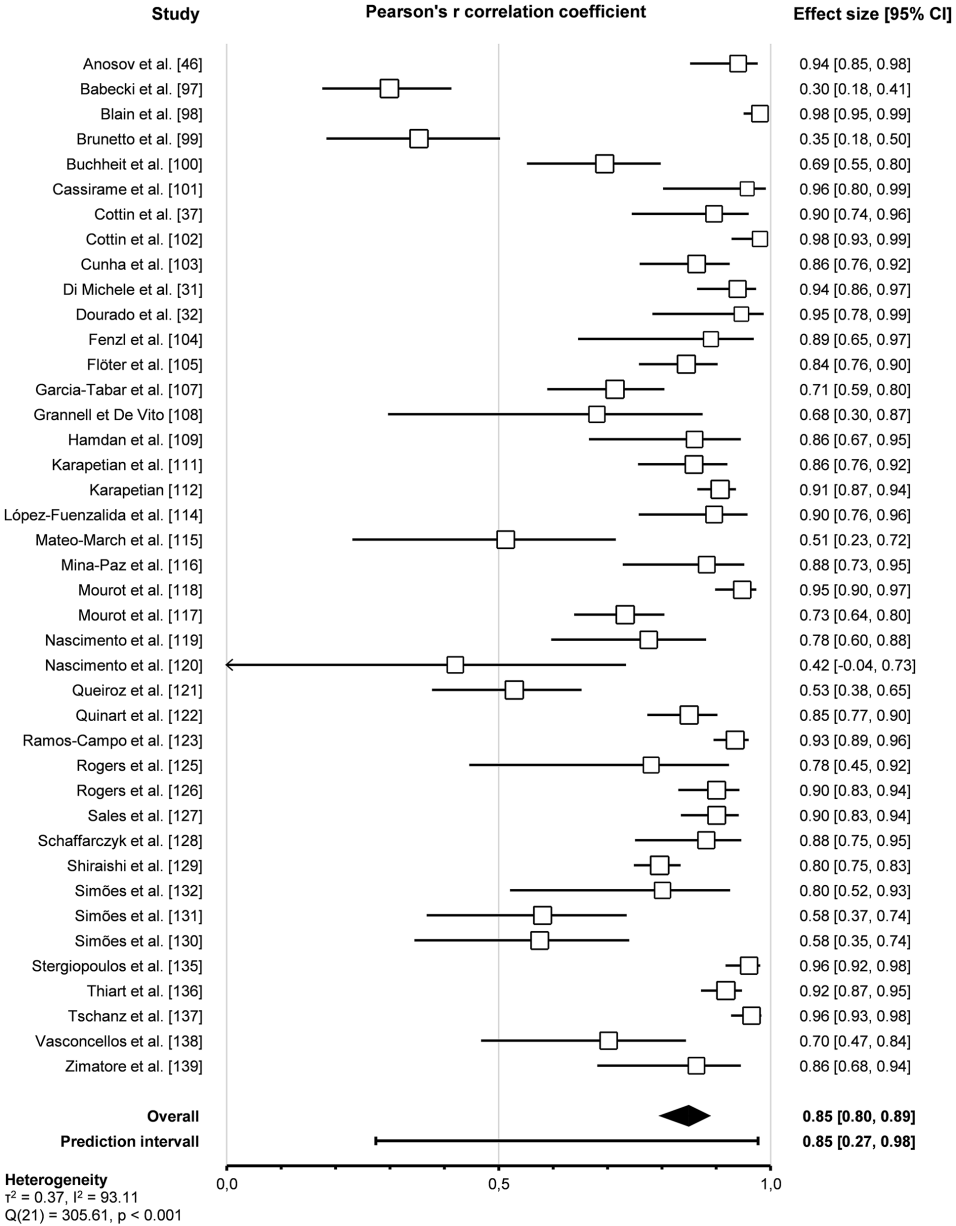
Forest plot of Pearson’s r correlation coefficient between HRVT2 and LT2-VT2 (random-effect model)

Pooled analysis of the 41 included studies assessing the correlation between HRVT2 and LT2-VT2 revealed a very strong correlation (Pearson’s *r* = 0.85, 95% CI 0.80–0.89, *p* < 0.001). The prediction interval ranged from 0.27 to 0.97, indicating that the true effect size falls within this interval in 95% of all comparable studies. The overall effect was heterogeneous (*p* < 0.001), suggesting that the true effect size was not the same in those 41 studies. Furthermore, the I^2^ statistic indicates that 92% of the variance in observed effects reflects variance in true effects rather than sampling error. The corresponding forest plot is shown in Fig. 12.

The observed heterogeneity in the HRVT2 primary analyses is high [140] indicating that the results of the included studies diverge from each other. As for HRVT1, conducting subgroup analyses and meta-regression, as presented below, is therefore relevant.

### Moderator Analyses for Second Heart Rate Variability Threshold

Since agreement and correlation meta-analyses between HRVT2 and LT2-VT2 showed significantly heterogeneous effects with 93% and 92% of the observed variance due to variance in true effects, subgroup analyses were performed. Pre-specified moderator variables were analysed separately to determine their influence on the standardised mean difference and the correlation between HRVT2 and LT2-VT2. A forest plot representation corresponding to each HRVT2 subgroup analysis, the subgroup’s heterogeneity assessment, and pairwise comparison p-value between subgroups (if statistical test for interaction was significant) can be found in Online Resource 6.

#### Subjects’ Characteristics

**Fig. 13:**
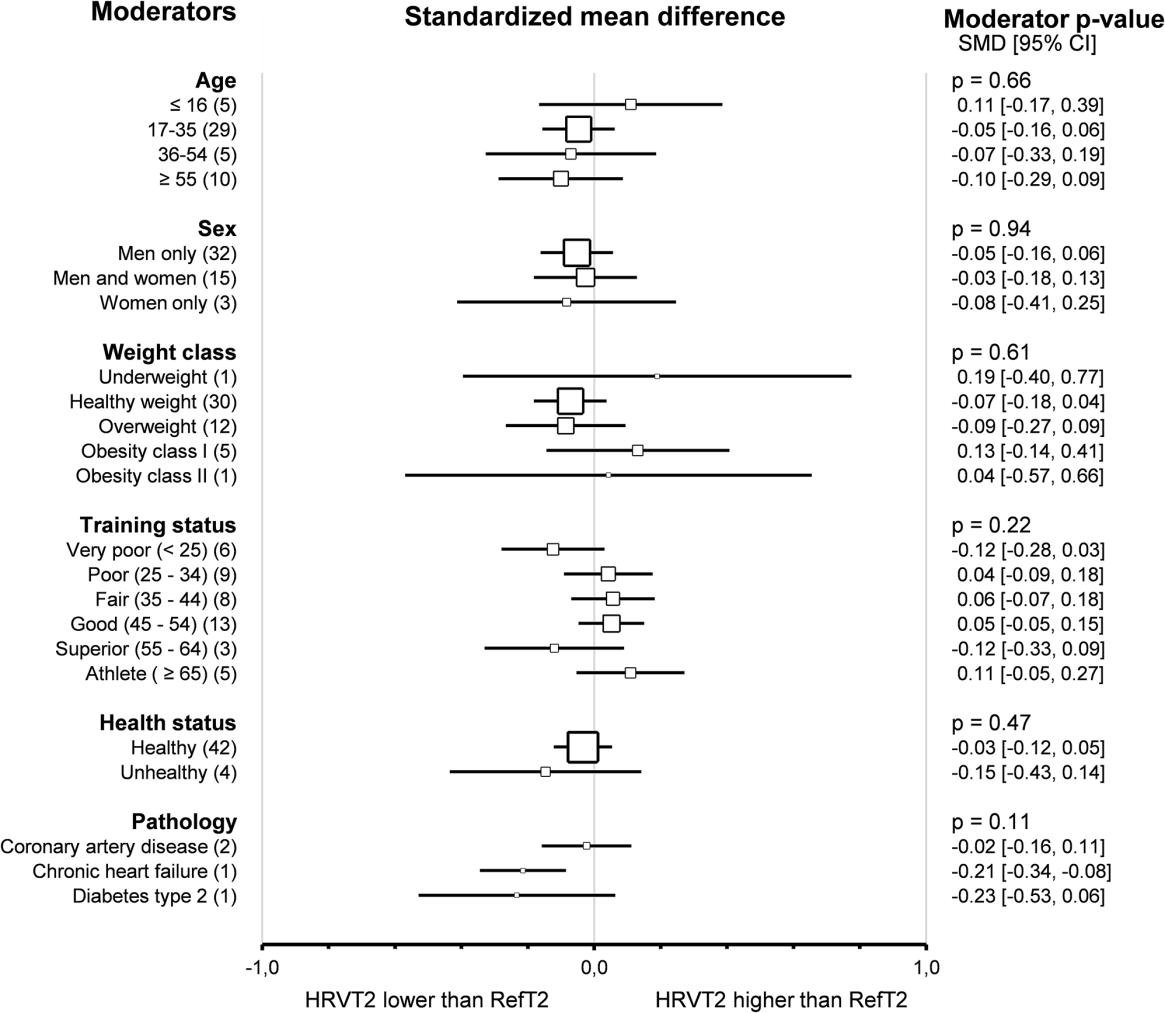
Forest Plots of agreement between HRVT2 and LT2-VT2 with subjects’ characteristics as moderators. Square sizes are proportional to the number of studies in subgroup. CAD, coronary artery disease; CHF, chronic heart failure; n, number of studies. Training status was classified according to V̇O_2_max (mL · min-1 · kg-1) as Very poor (< 25), Poor (25–34), Fair (35–44), Good (45–54), Superior (55–64), or Athlete (≥ 65). Weight class was classified according to BMI (kg/m^2^) as Underweight (< 18.5), Healthy weight (18.5–24), Overweight (25–29), Obesity class I (30–34), or Obesity class II (35–39)

**Fig. 14:**
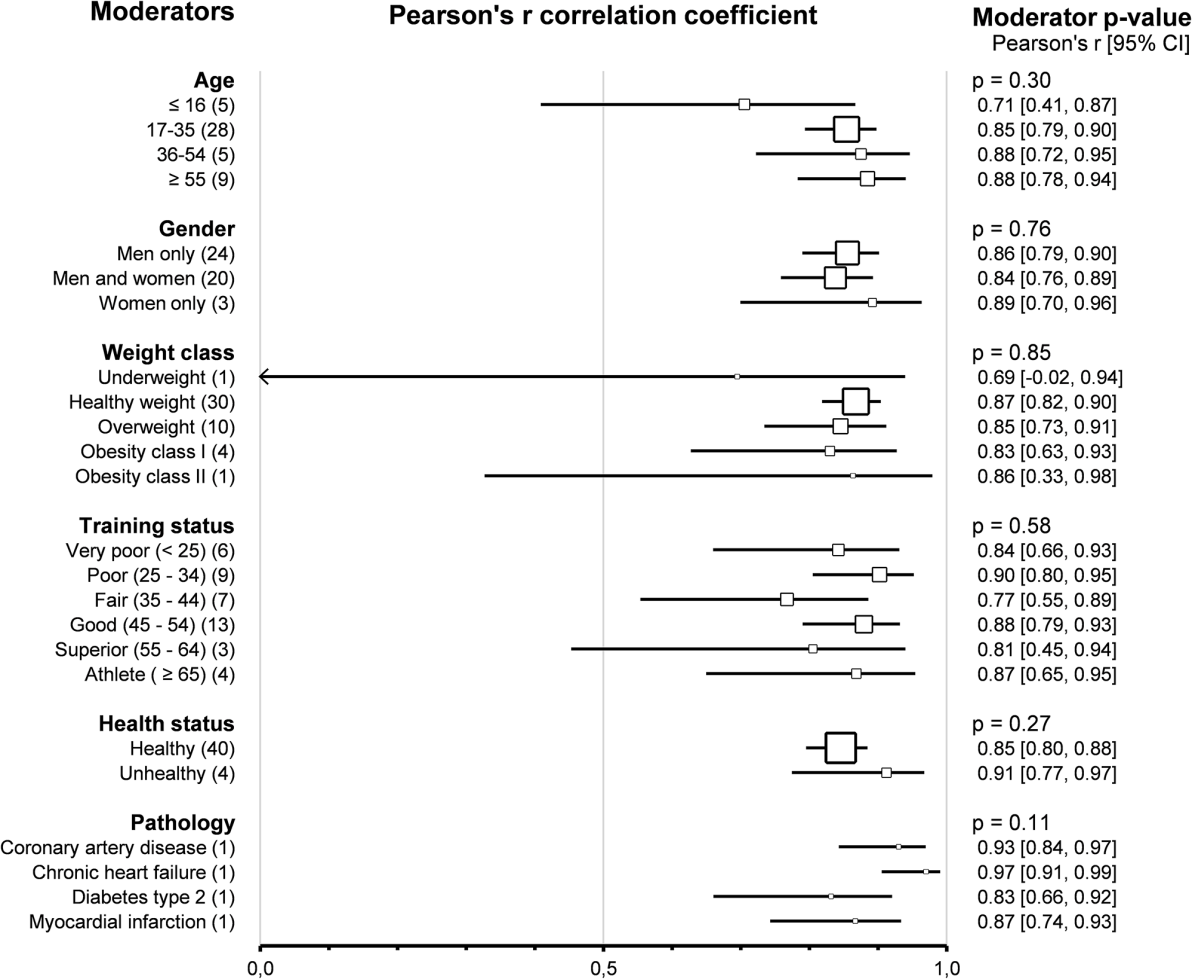
Forest Plots of correlation between HRVT2 and LT2-VT2 with subjects’ characteristics as moderators. Square sizes are proportional to the number of studies in subgroup. CAD, coronary artery disease; CHF, chronic heart failure; n, number of studies. Training status was classified according to V̇O_2_max (mL · min-1 · kg-1) as Very poor (< 25), Poor (25–34), Fair (35–44), Good (45–54), Superior (55–64), or Athlete (≥ 65). Weight class was classified according to BMI (kg/m^2^) as Underweight (< 18.5), Healthy weight (18.5–24), Overweight (25–29), Obesity class I (30–34), or Obesity class II (35–39)

Subgroup comparison analyses for subjects’ characteristics revealed that agreement and correlation between HRVT2 and LT2-VT2 were not impacted by *age* (*p* = 0.66 and *p* = 0.30 respectively), *sex* (*p* = 0.94 and *p* = 0.76 respectively), *weight class* (*p* = 0.61 and *p* = 0.85 respectively) and *training status* assessed by V̇O_2_max (*p* = 0.22 and *p* = 0.60 respectively). All these subgroup analyses were confirmed using meta-regressions on the corresponding continuous variable (age, % of men included BMI and V̇O_2_max), which showed no correlation between subjects’ characteristics and the corresponding effect size (SMD and Person’s r). Subjects’ *health status* did not impact the agreement and correlation between HRVT2 and LT2-VT2 (*p* = 0.47 and *p* = 0.27, respectively). Furthermore, the *pathology* (coronary artery disease [117, 134], myocardial infarction [29], cardiac heart failure [117] or diabetes type 2 [17]) affecting the patients included in this meta-analysis also showed no impact on the SMD and Pearson’s r between HRVT2 and LT2-VT2 (*p* = 0.11 and *p* = 0.06 respectively). Overall, none of the subjects’ characteristics impacted either the agreement or the correlation between HRVT1 and LT1-VT1. Details of subjects’ characteristics subgroup analyses as forest plots in Figs. 13 and 14.

#### Second Threshold Determination Methods

**Fig. 15:**
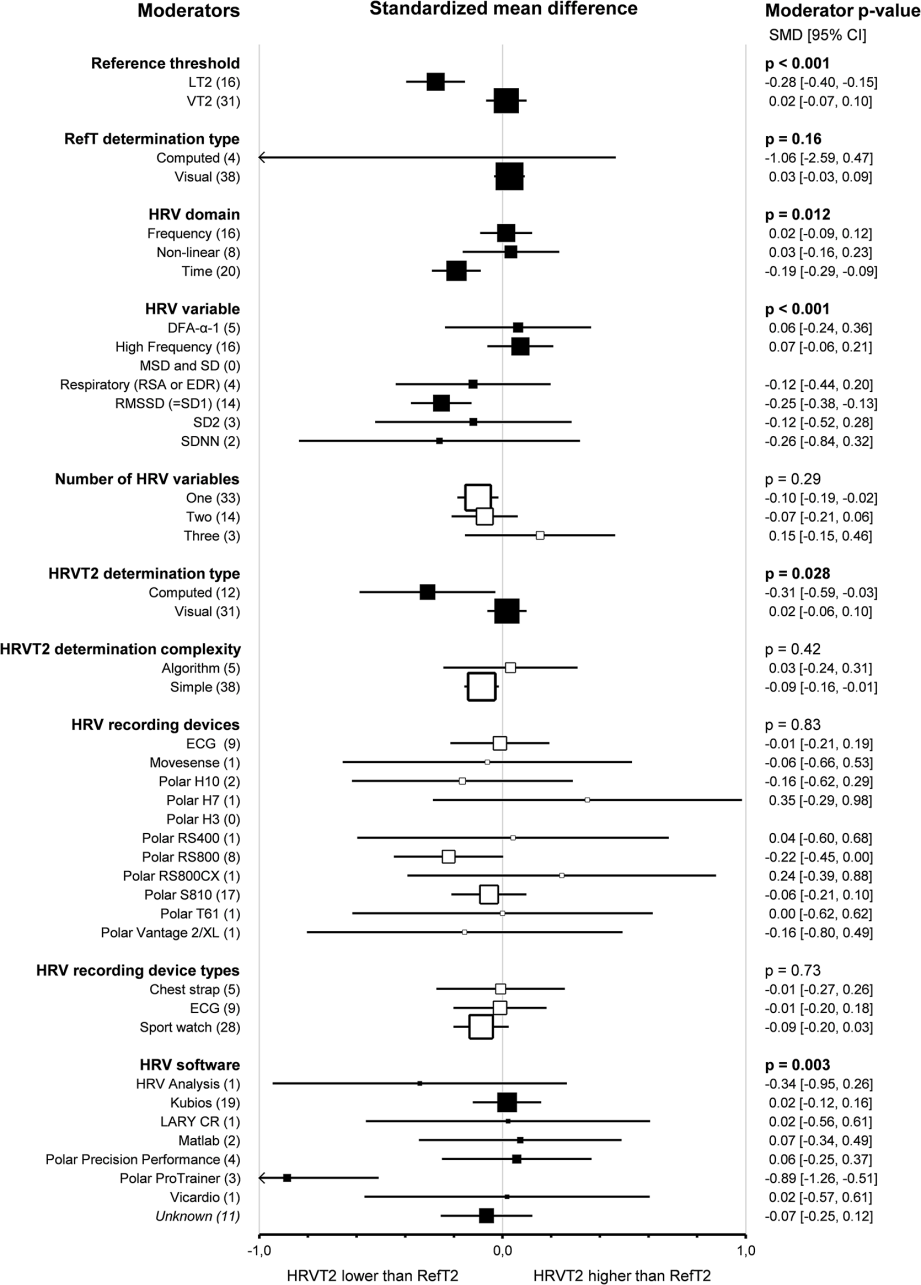
Forest Plots of agreement between HRVT2 and LT2-VT2 with thresholds determination characteristics as moderators. Solid black squares indicate moderators with significant impact on effect size. Square sizes are proportional to the number of studies in subgroup. DFA-ɑ1, detrended fluctuation analysis alpha 1; ECG, electrocardiogram; EDR, ECG derived respiration; HRVT2, heart rate variability threshold 2; MSD, mean successive differences; n, number of studies; LT2-VT2, reference threshold 2; RMSSD, root mean square of successive differences; RSA, respiratory sinus arrhythmia; SDNN, standard deviation of NN intervals; SD1, Poincaré plot standard deviation perpendicular the line of identity; SD2, Poincaré plot standard deviation along the line of identity

**Fig. 16:**
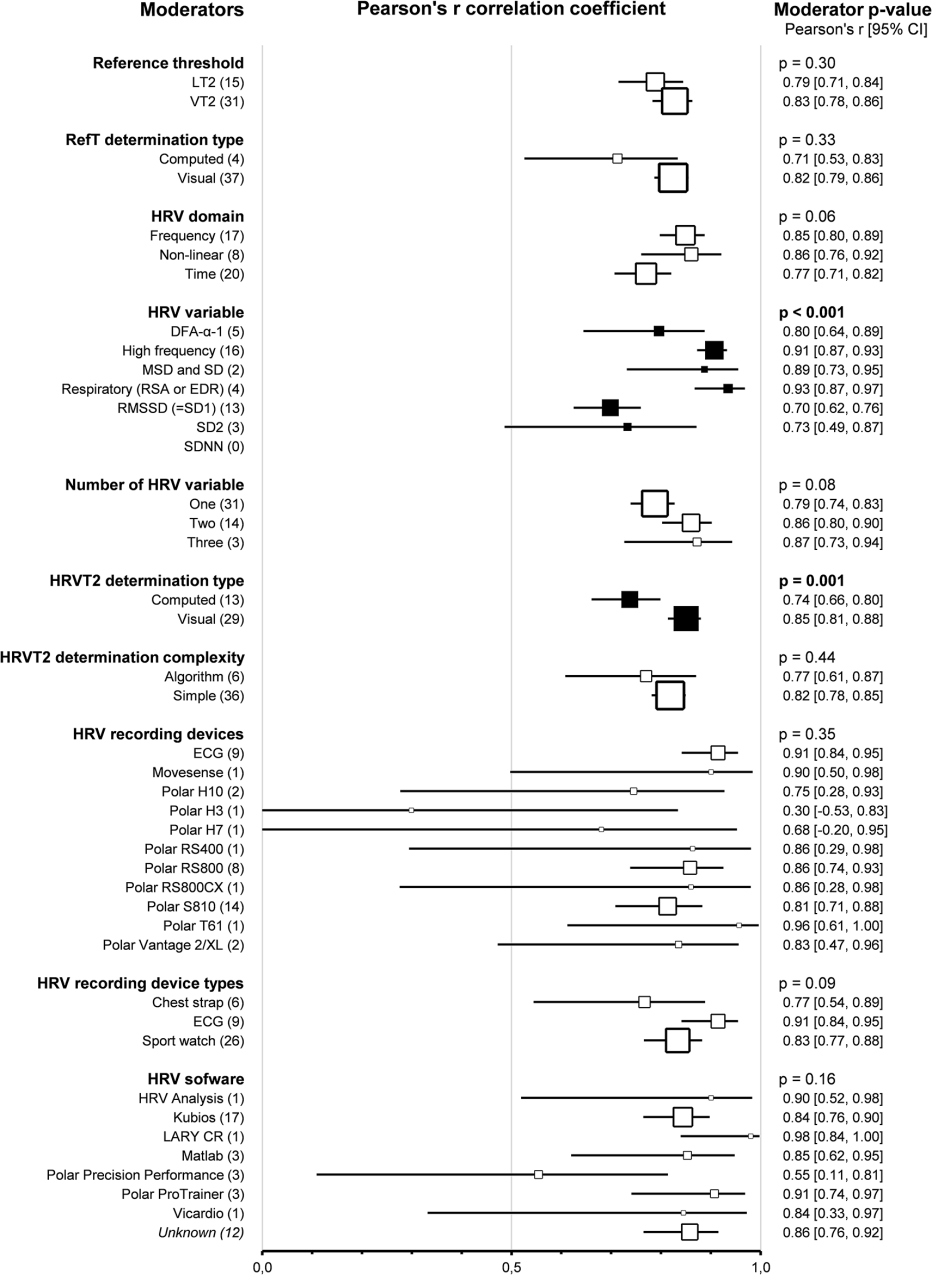
Forest Plots of correlation between HRVT2 and LT2-VT2 with thresholds determination characteristics as moderators. Solid black squares indicate moderators with significant impact on effect size. Square sizes are proportional to the number of studies in subgroup. DFA-ɑ1, detrended fluctuation analysis alpha 1; ECG, electrocardiogram; EDR, ECG derived respiration; HRVT2, heart rate variability threshold 2; MSD, mean successive differences; n, number of studies; LT2-VT2, reference threshold 2; RMSSD, root mean square of successive differences; RSA, respiratory sinus arrhythmia; SDNN, standard deviation of NN intervals; SD1, Poincaré plot standard deviation perpendicular the line of identity; SD2, Poincaré plot standard deviation along the line of identity

Subgroup comparison analyses for HRV and LT-VT methods revealed that *reference thresholds* impacted the agreement between HRVT2 and LT2-VT2 (*p* < 0.001). Indeed, HRVT2 was lower when compared to LT (-0.28, -0.40 – -0.15, *n* = 16) than when compared to VT (0.02, -0.07–0.10, *n* = 31, *p* < 0.001). Furthermore, when the LT was used as a reference for HRVT2 determination, there was a difference in agreement between LT2 and HRVT2 (*p* < 0.001). The reference threshold did not impact the correlation between HRVT2 and LT2-VT2 (*p* = 0.30). *Reference threshold determination type* (whether LT-VT was computed or visually determined) had no impact on agreement and correlation between HRVT2 and LT2-VT2 (*p* = 0.16 and *p* = 0.33, respectively). *HRV domains* used to determine HRVT2 impacted the agreement between HRVT2 and LT2-VT2 (*p* = 0.01). Indeed, when using time-domain HRV variables (-0.19, -0.29 – -0.09, *n* = 20), HRVT2 was lower than when using Frequency (0.02, -0.09–0.12, *n* = 16, *p* = 0.01) or Non-linear (0.03, -0.16–0.23, *n* = 8, *p* = 0.04) HRV variables. In addition, Time-domain derived HRVT2 were lower than LT2-VT2 (p = < 0.001). The domain of the HRV variable used had no impact on the correlation between HRVT2 and LT2-VT2 (*p* = 0.06). *HRV variables* used to determine HRVT2 impacted the agreement between HRVT2 and LT2-VT2 (*p* = 0.02). Indeed, the studies using RMSSD (-0.25, -0.38 – − 0.13, *n* = 14) obtained lower HRVT2 than studies using HF (0.07, -0.06–0.21, *n* = 16, *p* < 0.001). Furthermore, RMSSD-derived HRVT2 was lower than LT2-VT2 (*p* < 0.001). DFA-α1 derived HRVT2 (0.06, -0.24–0.36, *n* = 5) and HF-derived HRVT2 showed the best agreement with LT2-VT2, followed by Respiratory-derived HRVT2 (using respiratory sinus arrhythmia or ECG derived respiration) (-0.12, -0.44–0.20, *n* = 4), SD2 (-0.12, -0.52–0.28, *n* = 3), and SDNN (-0.26, -0.84–0.32, *n* = 2). The HRV variable also impacted the correlation between HRVT2 and LT2-VT2 (*p* < 0.001). Indeed, Pearson’s r was lower for RMSSD-derived HRVT2 (0.70, 0.62–0.76, *n* = 13) compared to HF (0.0.91, 0.87–0.93, *n* = 16, *p* < 0.001), Respiratory (0.93, 0.87–0.97, *n* = 4, *p* < 0.001) or Mean standard deviation-derived HRVT2 (0.89, 0.73–0.95, *n* = 2, *p* = 0.03). In addition, Pearson’s r was lower for SD2-derived HRVT2 (0.73, 0.49–0.87, *n* = 3) compared to HF (*p* = 0.01) or Respiratory derived HRVT2 (*p* = 0.01). Finally, Pearson’s r was lower for DFA-α1 derived HRVT2 (0.80, 0.64–0.89, *n* = 5) compared to HF (*p* = 0.02) or respiratory-derived HRVT2 (*p* = 0.02). HRV variables used only for one HRVT1 determination were not included in this subgroup analysis for reasons of clarity and robustness. The *number of HRV variables* used to determine each HRVT2 had no impact on the agreement between HRVT2 and LT2-VT2 (*p* = 0.29). HRVT2 determined with a Single HRV variable was lower than LT2-VT2 (-0.10, -0.19 – -0.02, *n* = 33, *p* = 0.02), but HRVT2 determined with Two (-0.07, -0.21–0.06, *n* = 14 [32, 37, 100, 102, 104–106, 116–118, 122, 126, 129, 135]) or Three (0.15, -0.15, 0.45, *n* = 3 [111, 129, 137]) HRV variable were not different than LT2-VT2. The number of HRV variables used did not impact the correlation between HRVT2 and LT2-VT2 (*p* = 0.08). The *HRVT2 determination type* (whether computed or visually determined) impacted the agreement and the correlation between HRVT2 and LT2-VT2. Indeed, the visual determination of HRVT2 showed better agreement (0.02, -0.06–0.10, *n* = 31) and stronger correlation (0.85, 0.81–0.88, *n* = 29) with LT2-VT2 than computed determinations (SMD = -0.31, -0.59 – -0.03, *n* = 12, *p* = 0.03; Pearson’s *r* = 0.74, 0.66–0.80, *n* = 13, *p* < 0.001). The *HRVT2 determination complexity* (whether the determination was algorithmic) had no impact on the agreement and correlation between HRVT2 and LT2-VT2 (*p* = 0.42 and *p* = 0.44, respectively). Of note, when HRVT2 determination was not algorithmic, it was lower than LT2-VT2 (-0.09, -0.16 – -0.01, *n* = 38, *p* = 0.02). The HRVT2 determination complexity did not impact the correlation between HRVT2 and LT2-VT2 (*p* = 0.44). *HRV recording devices* did not impact the agreement between HRVT2 and LT2-VT2 (*p* = 0.83). Moreover, none of the recording devices individually highlighted a difference in agreement between HRVT2 and LT2-VT2. However, HRV recording devices impacted the correlation between HRVT2 and LT2-VT2. Indeed, Pearson’s r was lower when using a Polar H3 (0.30, -0.53–0.83, *n* = 1) than ECG (0.91, 0.84–0.95, *n* = 9, *p* = 0.01), Polar RS800 (0.86, 0.74–0.93, *n* = 8, *p* = 0.045) or PolarT61 (0.96, 0.61, 0.99, *n* = 1, *p* = 0.04). *HRV recording device types* (whether chest strap, ECG, or sport watch was used) had no impact on the agreement and correlation between HRVT2 and LT2-VT2 (*p* = 0.73 and *p* = 0.09, respectively). Furthermore, none of the recording device types highlighted a difference in agreement between HRVT2 and LT2-VT2: Chest strap (-0.01, -0.27–0.26, *n* = 5), ECG (-0.01, -0.20–0.18, *n* = 9), sport watch (-0.09, -0.20–0.03, *n* = 28). *HRV software* impacted the agreement between HRVT2 and LT2-VT2 (*p* = 0.003). Indeed, the HRVT2 was statistically lower when using Polar ProTrainer (-0.89, -1.26 – -0.51, *n* = 3) compared to Kubios (-0.02, -0.12–0.16, *n* = 19, *p* < 0.001), Lary CR (0.02, -0.56–0.61, *n* = 1, *p* = 0.01), Matlab (0.07, -0.34–0.49, *n* = 2, *p* < 0.001), Polar precision performance (0.06, -0.25–0.37, *n* = 4, *p* < 0.001), Vicardio (0.02, -0.57–0.61, *n* = 1, *p* = 0.01) or if the software was not specified (-0.07, -0.25–0.12, *n* = 11, *p* < 0.001). The HRV software did not impact the correlation between HRVT2 and LT2-VT2 (*p* = 0.16). Details of thresholds determinations subgroup analyses are shown as forest plots in Figs. 15 and 16, in which solid black squares indicate moderators significantly impacting effect size.

#### Study Protocol

Subgroup comparison analyses for study protocols revealed that the *outcomes* impacted the agreement between HRVT2 and LT2-VT2 (*p* < 0.001). Indeed, HRVT2 was lower when expressed as a function of Power (-0.28, -0.39 – -0.18, *n* = 17) than as a function of Heart rate (0.01, -0.09–0.11, *n* = 20, *p* < 0.001), Speed (0.06, -0.11–0.23, *n* = 11, *p* < 0.001), or V̇O_2_ (0.04, -0.06–0.14, *n* = 16, *p* < 0.001). The SMD between HRVT2 and LT2-VT2 was equal to -0.08 (-0.34–0.19, *n* = 4) for Kg and − 0.07 (-0.35–0.21, *n* = 3) for Time. Outcomes also impacted the correlation between HRVT2 and LT2-VT2 (*p* = 0.04). Indeed, Pearson’s r was lower when HRVT2 was expressed as a function of Kg (0.66, 0.32–0.85, *n* = 3) or Time (0.67, 0.37–0.84, *n* = 3) compared to Heart rate (0.86, 0.81–0.90, *p* = 0.04 and *p* = 0.03 respectively) and Speed (0.87, 0.78–0.92, *p* = 0.048 and *p* = 0.04 respectively). *Outcome formats* impacted the agreement between HRVT2 and LT2-VT2 (*p* < 0.001). Indeed, when the outcomes were expressed as percentage values (-0.53, -0.70 – -0.37, *n* = 8), the HRVT2 was lower than when expressed as an absolute value (-0.01, -0.06–0.05, *n* = 41). However, the outcome format had no impact on the correlation between HRVT2 and LT2-VT2 expressed as absolute (0.84, 0.81–0.87, *n* = 41) or percentage (0.75, 0.62–0.84, *n* = 8) values (*p* = 0.06). *Ergometers* used for the incremental exercise test did not impact the agreement and correlation between HRVT2 and LT2-VT2 (*p* = 0.90 and *p* = 0.28, respectively).

**Fig. 17:**
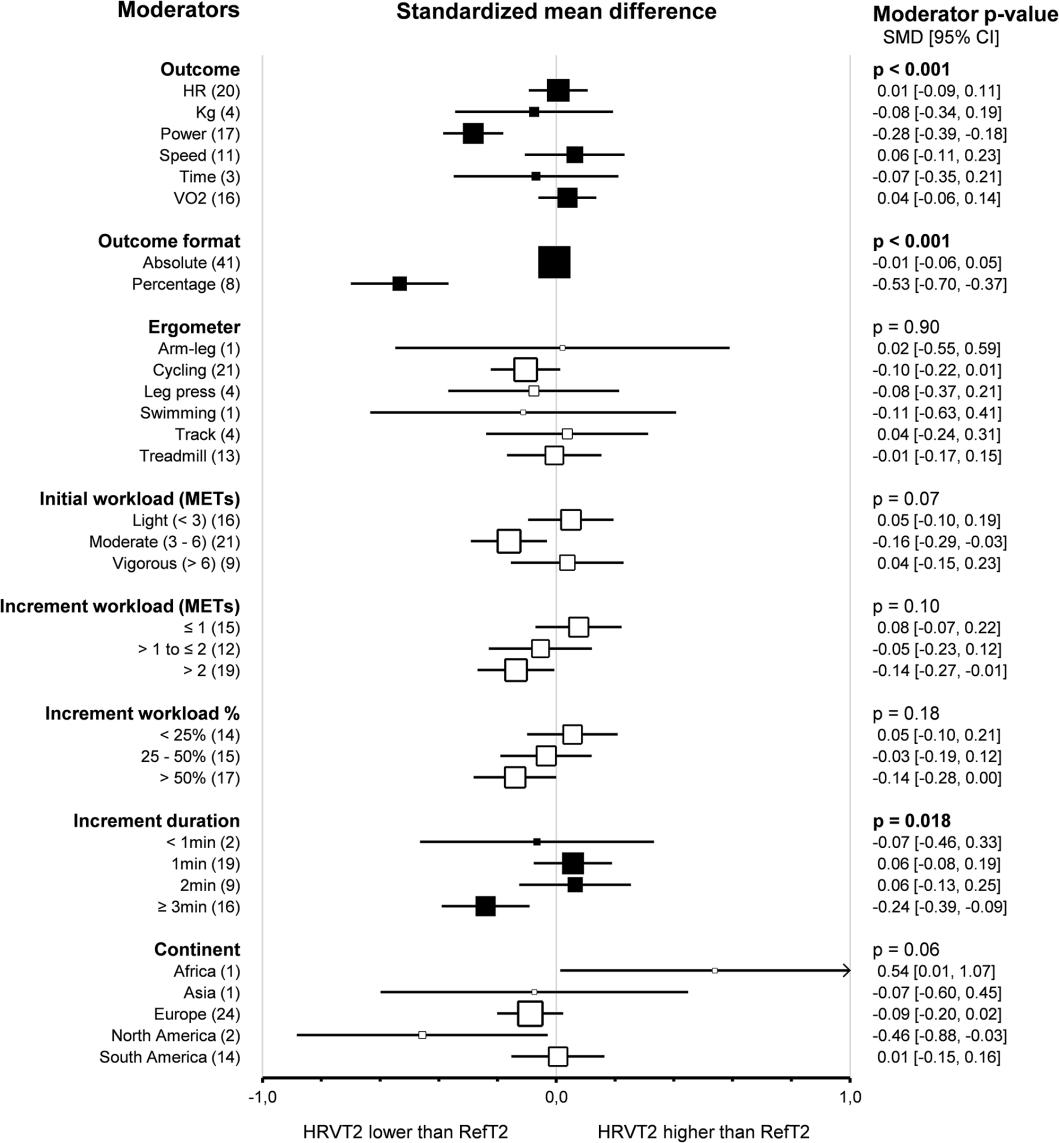
Forest Plots of agreement between HRVT2 and LT2-VT2 with study protocol characteristics as moderators. Solid black squares indicate moderators with significant impact on effect size. Square sizes are proportional to the number of studies in subgroup. MET, metabolic equivalent of task; n, number of studies. V̇O_2_max, oxygen consumption. Initial workload was classified according to the corresponding MET as Light (< 3), Moderate (3–6), or Vigorous (> 6)

**Fig. 18:**
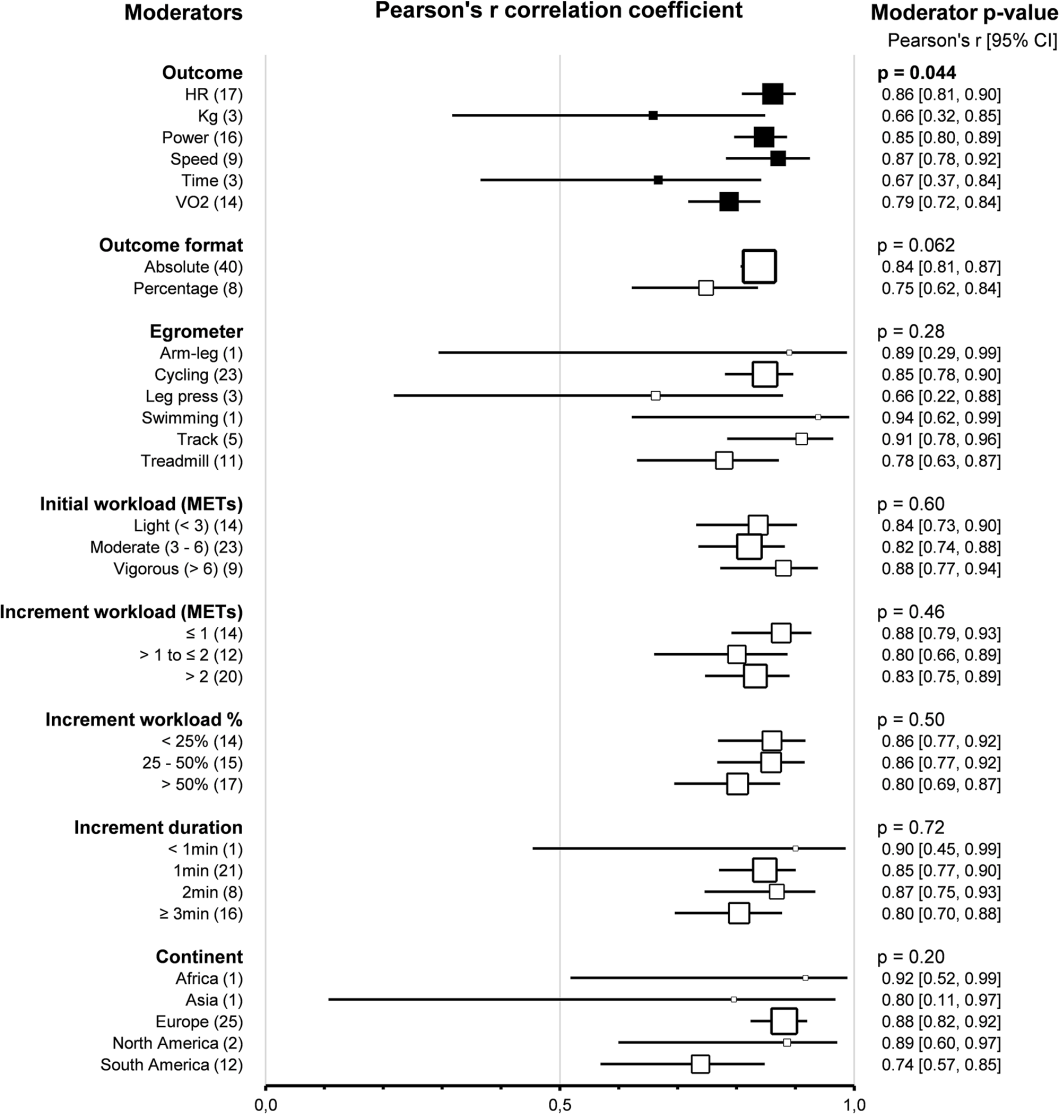
Forest Plots of correlation between HRVT2 and LT2-VT2 with study protocol characteristics as moderators. Solid black squares indicate moderators with significant impact on effect size. Square sizes are proportional to the number of studies in subgroup. MET, metabolic equivalent of task; n, number of studies. V̇O_2_max, oxygen consumption. Initial workload was classified according to the corresponding MET as Light (< 3), Moderate (3–6), or Vigorous (> 6)

Furthermore, subgroups analysis showed that *initial workload* in METs (*p* = 0.07, *p* = 0.60) and *increment workload* in METs (*p* = 0.10, *p* = 0.46) or percentage of initial workload (*p* = 0.18, *p* = 0.50) had no impact on the agreement and correlation between HRVT2 and LT2-VT2. All these subgroup analyses were confirmed using meta-regressions on the corresponding continuous variables, which showed no correlation between the characteristics of incremental test protocols and the corresponding effect size (SMD and Person’s r). However, the *increment duration* impacted the agreement (*p* = 0.02) but not the correlation (*p* = 0.72) between HRVT2 and LT2-VT2. Indeed, when 3 min increments or more were used (-0.24, -0.39 – -0.09, *n* = 16) during incremental exercise protocol, the HRVT2 determined was lower than with 1 (0.06, -0.08–0.19, *n* = 19, *p* = 0.04) or 2 min (0.06, -0.13–0.25, *n* = 9) increments. The *continent* where the study was conducted had no impact on the agreement and correlation between HRVT2 and LT2-VT2 (*p* = 0.06 and *p* = 0.20, respectively). Meta-regression analysis revealed that the *publication date* did not affect the agreement and correlation between HRVT2 and LT2-VT2 (*p* = 0.90 and *p* = 0.27, respectively). Furthermore, meta-regression showed that the SMD and Pearson’s r were unrelated to either the *study sample size* (*p* = 0.08 and *p* = 0.58, respectively) or the *number of comparisons* between HRVT2 and LT2-VT2 done in each study (*p* = 0.22 and *p* = 0.26, respectively). Details of study protocol subgroup analyses as forest plots in Figs. 17 and 18, in which solid black squares indicate moderators significantly impacting effect size.

### Risk of Bias Assessment

**Fig. 19:**
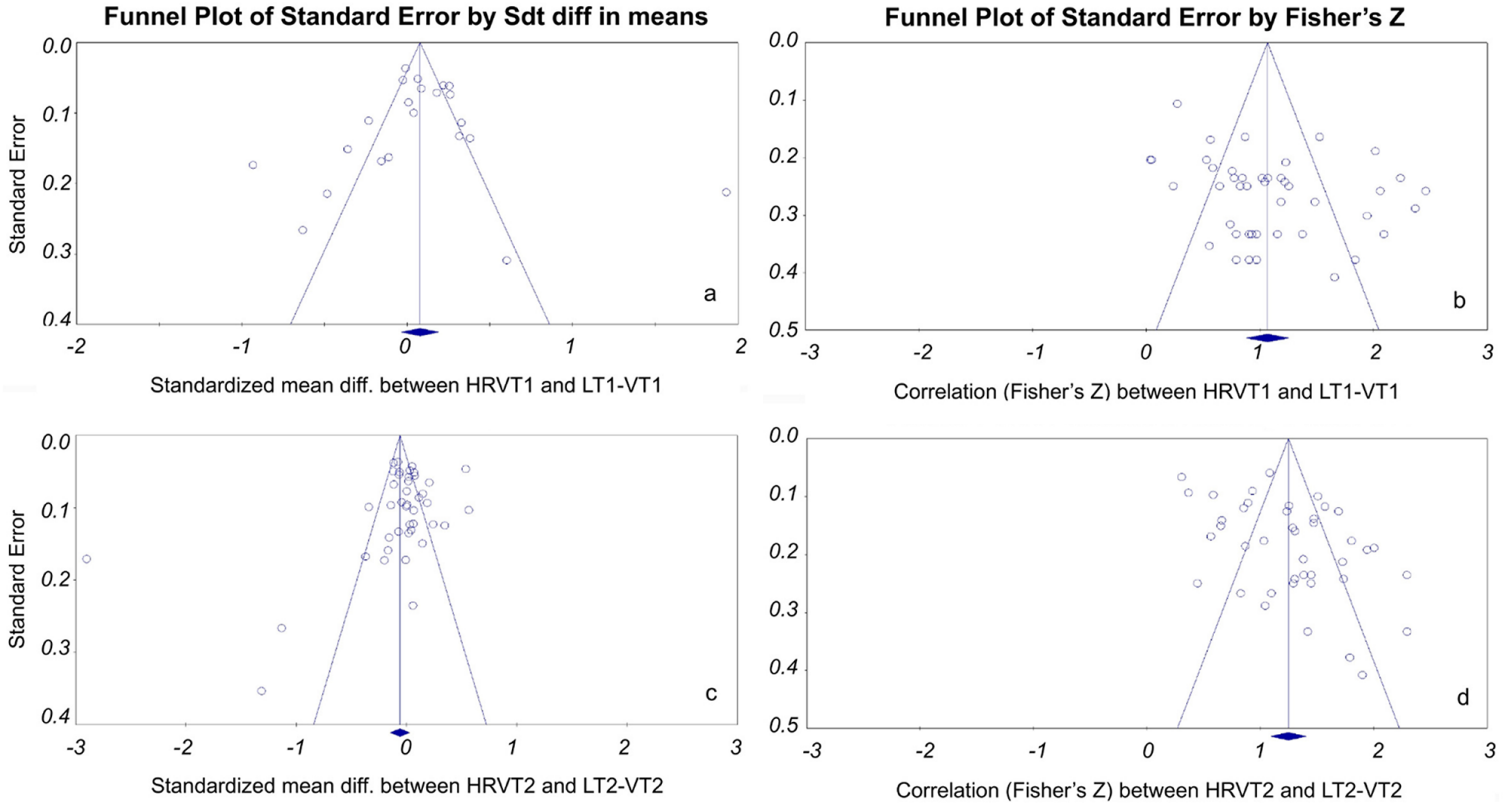
Funnel plots of selected studies for the four meta-analyses with x-axis representing effect size (standardized mean difference in panels “**a**” and “**c**”, and Fisher’s Z in panels “**b**” and “**d**”) and y-axis representing standard error; pseudo 95% confidence intervals are represented by two oblique lines, mean effect sizes are represented by vertical lines

The risk of bias assessment for the agreement meta-analysis between HRVT1 and LT1-VT1 showed a slightly asymmetrical funnel plot to the left (see Fig. 19a), no correlation between effect size and study sample size according to the Begg and Mazumdar rank correlation test (*p* = 0.43), and no significance of the Egger’s test (*p* = 0.92). The fail-safe N was not applicable since the combined standardised mean difference between HRVT1 and LT1-VT1 was not statistically significant (*p* = 0.18). The leave-one-out sensitivity analysis highlighted no outlier. Furthermore, none of the effect sizes computed after the sequential exclusion of each study showed a significant difference between HRVT1 and LT1-VT1. The RoB assessment for the correlation meta-analysis between HRVT1 and LT1-VT1 showed a symmetrical funnel plot (see Fig. 19b), no correlation between effect size and study sample size according to the Begg and Mazumdar rank correlation test (*p* = 0.14), and a significant Egger’s test of the intercept (*p* < 0.001). The fail-safe N suggested that 9644 null effects studies would be required to overturn the overall significant correlation between HRVT1 and LT1-VT1. The leave-one-out sensitivity analysis highlighted no outlier.

The RoB assessment for the LT2-VT2 – HRVT2 agreement meta-analysis showed an asymmetrical funnel plot to the right (see Fig. 19c), no correlation between effect size and study sample size according to the Begg and Mazumdar rank correlation test (*p* = 0.19), and no significance of the Egger’s test (*p* = 0.15). The fail-safe N was not applicable since the combined standardised mean difference between HRVT1 and LT1-VT1 was not statistically significant (*p* = 0.19). The leave-one-out sensitivity analysis highlighted no outlier. Furthermore, none of the effect sizes computed after the sequential exclusion of each study showed a significant difference between HRVT2 and LT2-VT2. The RoB assessment for the LT2-VT2 – HRVT2 correlation meta-analysis showed a slight asymmetric funnel plot to the right (see Fig. 19d), no correlation between effect size and study sample size according to the Begg and Mazumdar rank correlation test (*p* = 0.20), and a significant Egger’s test of the intercept (*p* = 0.002). The fail-safe N suggested that 24,200 null effects studies would be required to overturn the overall significant correlation between HRVT1 and LT1-VT1. The leave-one-out sensitivity analysis highlighted no outlier.

### Certainty Assessment

As the studies included were not randomised controlled trials, the level of evidence was considered low a priori [94]. Thus, low-certainty evidence indicates that HRV thresholds (HRVT1 and HRVT2) are not statistically different from reference thresholds (LT1-VT1 and LT2-VT2). Moderate-certainty evidence indicates that HRV thresholds are correlated with reference thresholds. Indeed, the evidence for both correlation meta-analyses was upgraded once because of the large magnitude of the effect and its narrow confidence interval.

## Discussion

This systematic review with meta-analyses is the first to compute overall effect sizes to assess the agreement and correlation between heart rate variability thresholds (HRVT1/HRVT2) and reference – lactate and ventilatory – thresholds (LT1-VT1/LT2-VT2). Furthermore, for the first time, the impact of the subjects’ characteristics, HRV methods, and study protocols on the agreement and correlation between LT-VTs and HRVTs was assessed comprehensively and methodically. HRVT1 and HRVT2 showed trivial standardised mean differences (SMD = 0.08 and SMD = -0.06) and very strong correlations (*r* = 0.84 and *r* = 0.85) with LT1-VT1 and LT2-VT2, respectively. None of the subjects’ characteristics impacted either the agreement or the correlation between HRVTs and LT-VTs, but some HRV methods and study protocol-related variables did. The results of relevant moderator analyses are discussed below. Details of all moderator analyses for HRVT1 and HRVT2 can be found in Online Resource 5 and Online Resource 6, respectively.

A few methodological considerations are required to interpret these meta-analyses results further. The agreement and correlation between HRVT1/HRVT2 and LT1-VT1/LT2-VT2, respectively, were assessed regardless of the type (LT or VT) and method by which the reference thresholds were determined, which raises two points. Firstly, the agreement between LTs and VTs is still debatable [143–145], but there is a growing body of evidence to view them as closely related [2, 14, 27, 146, 147]. Secondly, the various methods used to determine LTs and VTs can lead to divergent results. Although all the included studies compared HRVTs to LT-VTs derived from pre-established, validated, and widely used determination methods, the latter may not be equivalent depending on the context. However, given the lack of meta-analysis on HRVTs determination to date, this review focused on the characteristics of the methods used to determine HRVTs. These HRVTs were thus compared with their corresponding LT-VTs, regardless of their determination methods, allowing this review to be more straightforward and emphasise the option of HRVTs as a potential solution to the multiple LT-VTs determination methods issue. All in all, the following results obtained by comparing studies using LTs and VTs as references should be interpreted cautiously and considering the aforementioned elements.

Concerning the applicability of the present meta-analyses results, it should be noted that the SMD is widely used as an agreement effect size index when studies assess the same outcome but measure it in different ways [55]. However, the SMD has the disadvantage of not being expressed in easily interpretable units. Nevertheless, the SMD is more generalizable than the mean difference [148]. In this context, since LTs and VTs are closely related [2, 14, 27, 146, 147] and allow for training prescription, planning, and control [14], comparing their agreement and correlation to the agreement and correlation between HRVTs and LT-VTs might help to determine if HRVTs could be used as a surrogate for LT-VTs. The overall agreements and correlations between HRVTs and LT-VTs yielded by our meta-analyses are in the range of values reported for VTs – LTs comparisons [13, 149–151], as mentioned by [52]. Moreover, according to the computation proposed by Grice and Barret [152], who revised Cohen’s overlapping proportions [75], the overlap in agreement between HRVT1/HRVT2 and LT1-VT1/LT2-VT2 is equal to 96.9% (SMD = 0.08) and 97.7% (SMD = -0.06) respectively. Altogether, these findings suggest that, in given situations detailed in the moderator analysis thereafter, HRVTs might be an appropriate surrogate for conventional reference thresholds when taken as a whole.

### Moderator Analyses for First Heart Rate Variability Threshold Determination

Our analyses revealed that subjects’ characteristics such as age, sex, weight class or training status have no significant impact on HRVT1 determination. Even varying health conditions, including coronary artery disease and cardiac heart failure, did not exhibit significant differences in HRVT1 agreement and correlation. However, the latter statement about health conditions is limited by the small number of studies including patients in their protocol [113, 117, 124, 133]. A more detailed analysis of the various demographic characteristics yielded some interesting findings. Indeed, *ageing* is associated with a decrease in HRV, primarily due to decreased parasympathetic modulation [36, 39, 153, 154], and lower time domain HRV indices were observed in elderly subjects at rest and during exercise compared to young subjects [131]. However, since HRVT1 determination is not impacted by age, this suggests that, despite lower levels in elderly subjects, HRV variations and dynamics still allow for precise HRVT1 determination. In addition, the higher vagal activity in premenopausal *women* [155] and the impact of ovarian hormones during the menstrual cycle on autonomic tone [156, 157] do not appear to interfere with HRVT1 determination despite the previously described issues surrounding agreement between HRVT1 and VT1 in women [128]. The fact that one of the two studies determining HRVT1 in women only [116, 128] enrolled professional cyclists may explain why the overall results of HRVT1 determination in women are similar to those of men. Indeed, reduced ovarian hormones [158, 159] and athletic oligo- or amenorrhea [160] are common in female elite endurance athletes and may result in HRV activity comparable with men. Concerning *training status*, some previous considerations [108, 131], such as different heart rate acceleration dynamics between trained and untrained subjects [161] which may account for earlier vagal withdrawal in trained subjects [161, 162] or the impact of V̇O_2_max on cardiac autonomic control [162–164] suggested that physical condition may influence HRVTs determination. According to our results, these differences in the autonomic nervous system activation among different aerobic capacities, however, do not appear to impact HRVT1 determination directly. Finally, despite the low parasympathetic modulation in *obese* [121], diabetic [127] or cardiac [117] *patients* and the multiple influences of their various medications on HRV [113], HRVT1 had a good overall agreement and correlation with LT1-VT1. These findings highlight the potential applicability and suggest that HRVT1 determination remains consistent across different population demographics.

The analyses regarding determination methods for HRVT1 and LT1-VT1 showed interesting and contrasting influence patterns on agreement and correlation. The *reference threshold* used (lactate or ventilatory) significantly impacted agreement, with HRVT1 showing better agreement when compared to LT than to VT. Furthermore, contrary to previous results [52, 127], HRVT1 values were, on average, higher than VTs but lower than LTs. Different LT-VT determination methods may explain these discrepancies in results [8, 165]. This difference in agreement did not affect the correlation between HRVT1 and LT1-VT1, indicating that while agreement might vary, the overall correlation remained strong. The *domain of HRV variables* used to determine HRVT1 had no impact on the agreement or the correlation between HRVT1 and LT1-VT1. Indeed, neither the limitation of the non-linear methods mentioned by [53] (intrinsic individual variability, accumulation of sampling error, non-stationarity or dependence on the parametric values) nor the putative superiority of frequency-domain over linear-domain for HRVT determination [122] were observed in the present agreements results. In fact, the time-domain showed a non-significant tendency to have better agreement with LT-VTs than frequency or non-linear domain. In addition, the *HRV variables* used for HRVT1 determination did not significantly affect the agreement between HRVT1 and LT1-VT1 but had an impact on their correlation. Indeed, RMSSD-derived HRVT1 yielded a lower correlation than HF-related HRVT1, which may be explained by the fact that information about breathing mechanics is embedded in the HF signal. In contrast, RMSSD reflects primarily the activity of the autonomic nervous system itself [122, 166]. Furthermore, the *method used to determine HRVT1* had no impact on the agreement between HRVT1 and LT1-VT1 but visually determined HRVT1 yielded higher correlation with LT1-VT1 than computed ones. The latter is in line with previous results showing that visual determinations had higher reliability than computed methods [20, 167]. The *determination complexity of HRVT1* significantly impacted both agreement and correlation, with simpler determination methods resulting in better agreement and stronger correlation than algorithmic methods. This suggests that a straightforward approach to HRVT1 determination may yield more reliable results and that algorithmic determinations sometimes described as promising are not, to date, superior for HRVT1 determination.

The moderator analysis for studies protocols showed contrasting influence patterns on agreement and correlation between HRVT1 and LT1-VT1. On the one hand, the *outcomes* used to assess HRVT1 did not significantly impact the agreement between HRVT1 and LT1-VT1 but influenced correlation, with outcomes expressed as time resulting in lower correlation compared to heart rate (bpm), power (W) and V̇O_2_ (mL · min^−1^ · kg^−1^). This suggests that the outcome variables may affect the strength of the correlation between HRVT1 and LT1-VT1. However, further practical implications remain to be clarified, especially since only two studies used speed to assess HRVT1. In this context, it is noteworthy to emphasise that the units used to assess HRVTs are important. Indeed, when expressed in km/h (speed) or W (power), for example, HRVTs do not measure only aerobic endurance but also V̇O_2_max and mechanical efficiency [6]. Moreover, whether expressed as absolute values or percentages, the *outcome format* significantly impacted the agreement between HRVT1 and LT1-VT1. Indeed, HRVT1 expressed in percentage values resulted in a worse agreement and lower HRVT1 values than when expressed in absolute values. However, this difference did not affect correlation, indicating that the format of outcomes may influence the absolute values of HRVT1 but not its relationship with LT1-VT1. Conversely, none of the incremental exercise protocol characteristics impacted the agreement or correlation between HRVT1 and LT1-VT1. Firstly, the *ergometer* used for the incremental exercise test (cycling, treadmill, running track or even leg-press) did not influence HRVT1 determination, confirming and generalizing previous results. Indeed, HRVT1 has already been reliably determined across various ergometers such as cycle ergometry [167, 168] and treadmill [110]. However, some mentions in the literature seemed to suggest that, when using frequency domain HRV variables, the results obtained for HRVT1 determination with a treadmill and a cyclo-ergometer are not concordant [20, 169–171]. This seemed to be explained by the fact that HRVT1 may happen simultaneously with the transition between walking and running [170], which does not occur using cycle ergometry. Furthermore, the walking–running transition may alter physiological variables (HR, V̇E, and V̇O_2_ among other), causing interference in autonomic control and thus making the interpretation of HRV parameters to identify HRVT1 more challenging [170]. In addition, since the cadence is typically maintained constant on the cycle-ergometer, the influence of the increased striding frequency inherent to running during an incremental exercise test may influence the breathing frequency and thus cause further contrasting HRV dynamics between treadmill or track ergometers and cycle-ergometry [100, 172]. Overall, none of this inter-ergometer variation was confirmed either on agreement, or on correlation between HRVT1 and LT1-VT1 by the present moderator analysis, suggesting that HRVT1 determination remains consistent across different ergometers. Secondly, neither the *initial workload* nor the *incremental workload or duration* impacted the agreement and correlation between HRVT1 and LT1-VT1, which confirmed and extended previous findings obtained on cycle ergometer [173].

### Moderator Analyses for Second Heart Rate Variability Threshold Determination

Only the new elements specific to the determination of HRVT2 are discussed here for clarity and concision. Indeed, the moderator analyses for HRVT1 and HRVT2 revealed substantial similarities, and the considerations when discussing HRVT1 determinations also apply for HRVT2.

As for HRVT1, the moderator analysis revealed that subjects’ characteristics, including age, sex, weight class, training and health status, did not significantly impact the agreement or correlation between HRVT2 and LT2-VT2. Additionally, despite the small number of studies that included patients [117, 127, 129, 134], specific pathologies such as coronary artery disease, myocardial infarction, chronic heart failure, or type 2 diabetes did not influence either the agreement or the correlation between HRVT2 and LT2-VT2. Those results are not surprising given that intensities at HRVT2 are demanding, require intense autonomic modulations [101] and correspond to a loss of physiological sustainability and organismic destabilisation [125, 128]. Those physiological adaptations may, therefore, result in better recognition of inflexion points and less discrepancy between LT2-VT2 and HRVT2 [123] and might be more resistant to external influence than HRVT1. The latter has already been shown for the impact of hormonal change. Indeed, comparing HRVT2 determination in men and women yielded similar results [128].

The moderator analyses regarding determination methods for HRVT2 and LT2-VT2 showed similarities with those concerning HRVT1 and LT1-VT1. Indeed, *reference threshold* used (lactate or ventilatory) also significantly impacted agreement, with HRVT2 values on average slightly higher than VTs and lower than LTs. However, contrary to HRVT1, HRVT2 showed better agreement when compared to VTs than LTs. However, contrary to HRVT1, the *domain of HRV variables* used to determine HRVT2 impacted the agreement between HRVT2 and LT2-VT2. Indeed, time-domain derived HRVT2 showed significantly worse agreement than frequency-domain or non-linear HRVT2 determinations. This poorer agreement and difference between HRVT1 and HRVT2 can be explained by the low signal-to-noise ratio in time-domain HRV indices at exercise intensities corresponding to HRVT2, as previously described [52]. In addition, time-domain showed a non-significant tendency also to yield a weaker correlation between HRVT2 and LT2-VT2. Furthermore, analyses of *HRV variables* confirmed those results with time-domain HRV variables (RMSSD and SDNN) showing worse agreements and weaker correlations between HRVT2 and LT2-VT2 than other frequency or non-linear indices. The *method used to determine HRVT2* impacted the agreement between HRVT2 and LT2-VT2. Indeed, computed determination showed worse agreement than visually determined HRVT2. Moreover, as for HRVT1, visually determined HRVT2 yielded a higher correlation with LT2-VT2 than computed methods, confirming that visual methods are, to date, still superior for HRVT determinations. Unlike HRVT1, *HRVT2 determination complexity* did not impact the agreement and correlation. Nevertheless, since more complicated methods do not provide better results, the conclusion is the same as for HRVT1 determination: promising algorithmic methods are not yet superior to simple methods for HRVT determination.

The analysis of studies protocols showed contrasting patterns of influence on agreement and correlation between HRVT2 and LT2-VT2 as for HRVT1. On the one hand, the *outcomes* used to assess HRVT2 impacted agreement and correlation between HRVT2 and LT2-VT2. Indeed, when power (W) was used to express HRVT2, it resulted in lower HRVT2 than when expressed as heart rate (bpm), speed (km/h) or V̇O_2_ (mL · min^−1^ · kg^−1^). Moreover, the correlation between HRVT2 and LT2-VT2 was weaker when expressed as a function of Kg or time (s) compared to heart rate and speed. The choice of outcome variable may affect the agreement and the strength of the correlation between HRVT2 and LT2-VT2. The *outcomes format* had a similar impact on HRVT2 than on HRVT1. Indeed, HRVT2 expressed in percentage values also resulted in a worse agreement and lower HRVT2 values than when expressed in absolute values, and this difference did not affect correlation. On the other hand, as for HRVT1, the majority of the incremental exercise protocol characteristics did not impact the agreement or correlation between HRVT2 and LT2-VT2, suggesting that ergometer, initial and incremental workload did not significantly impact the agreement or correlation between HRVT2 and LT2-VT2. Indeed, the different *ergometers* used (even those involving the upper body, such as swimming or those for simultaneous arms and legs movements) showed no significant difference in HRVT2 determination. It is noteworthy because some HRV parameters, especially frequency-domain HRV indices, are more likely to be affected by upper body movements at high intensity corresponding to HRVT2 than at relatively low HRVT1 intensity. It should also be noted that, unlike for HRVT1, HRVT2 determination was impacted by the *increment duration*. Indeed, the agreement between HRVT2 and LT2-VT2 was worse, and HRVT2 values were lower than LT2-VT2 when increments of 3 min or more were used. Using such long increments in included studies is understandable since it allows for better stability in the RR intervals [112]. However, unfortunately, it also reduces the accuracy of the V̇O_2_max estimation [87] and thus might explain the lower agreement between HRVT2 and LT2-VT2.

### Comparison of First vs. Second Heart Rate Variability Threshold Determination

The moderator analyses for HRVT1 and HRVT2 revealed many similarities, demonstrating the robustness of the analyses performed in this review. However, contrasting results were shown regarding the impact of the reference threshold chosen. Indeed, HRVT1 – VT1 and HRVT2 – LT2 values disagreed significantly, whereas there was good agreement between HRVT1 and LT1 and between HRVT2 and VT2. This suggests that HRVT1 better agree with LT1 and HRVT2 better agree with VT2. Furthermore, the agreement between HRVTs and their respective LT-VTs highlights an interesting pattern. Indeed, both HRVTs were defined above their corresponding VTs but below their lactic thresholds. At this point, it is not possible to state that HRVTs lie between ventilatory and lactic thresholds, especially since the included studies were not designed to compare LTs to VTs. Nevertheless, these results demonstrate the absence of unidirectional bias and strong correlation but ambiguous agreement between HRVTs and LT-VTs.

### Methodological Quality Assessment

The QUADAS-2 assessment revealed a generally low risk of bias across its four domains, with most studies demonstrating low bias in flow and timing (88%), reference standard (84%), patient selection (80%), and index test (64%). Furthermore, the applicability of the results of included studies was excellent, as low concerns for applicability were reported for the three corresponding domains in 98% (reference standard), 90% (index test), and 86% (patient selection) of included studies. Methodological quality assessment using the adapted STARD_HRV_ provides a more nuanced evaluation. The included studies achieved an average score of 78 ± 8%. The distribution of scores indicates that while half of the included studies showed good HRV methodology (STARD_HRV_ score ≥ 80%), there is still room for improvement, as 20% of studies scored < 70%. Improvement is particularly needed in information about sample size determination, mention of a stabilization period prior to HRV sampling, and specification of whether breathing was controlled during HRV recording since these three items were often underreported. Meanwhile, some areas where most studies performed well, such as validation study designation, structured abstracts, background clarity, within-subject design, and extensive description of setups and protocols, highlight the strengths of current research practices in HRVTs determination but were also often inclusion criteria for the studies in this systematic review. Altogether, the QUADAS-2 and STARD_HRV_ assessments indicated a predominantly low RoB, good applicability and moderate to good HRV-related methodology in included studies, providing an appropriate basis for our data analyses and interpretations.

### Risk of Bias Assessment

The slightly asymmetrical funnel plot to the left for the agreement meta-analysis between HRVT1 and LT1-VT1 suggested a minor publication bias or small study effects favouring smaller studies. However, the statistical tests do not support this visual inspection. The lack of correlation between effect size and study sample size and a non-significant Egger’s test indicates no firm evidence of publication bias. Moreover, no outliers were identified during the leave-one-out sensitivity analysis. Concerning the correlation meta-analysis between HRVT1 and LT1-VT1, the RoB assessment showed that, while there might not be a visual indication of bias (symmetrical funnel plot), a significant Egger test suggests potential RoB. However, the fail-safe N indicated that an extremely large number of unpublished or null studies would be needed to invalidate the significant correlation between HRVT1 and LT1-VT1, and the leave-one-out sensitivity analysis found no outliers, thus supporting the robustness of this correlation.

An asymmetrical funnel plot to the right for the agreement meta-analysis between HRVT2 and LT2-VT2 suggested potential publication bias, yet this is not corroborated by Begg and Mazumdar (*p* = 0.19) or Egger’s test (*p* = 0.15), suggesting no firm evidence of bias, which is reinforced by the absence of outliers or significant changes in effect size upon sequential study exclusion in the leave-one-out sensitivity analysis. Concerning the correlation meta-analysis between HRVT2 and LT2-VT2, a slight asymmetry in the funnel plot and the significant Egger’s test suggested the presence of bias. However, this is not confirmed by the Begg and Mazumdar test. First and foremost, the extremely large fail-safe N suggests a robust correlation between HRVT2 and LT2-VT2 that unpublished or additional studies would not easily overturn. The consistency of the correlation is further supported by the leave-one-out analysis, which identified no influential outliers.

In conclusion, while there are some indications of potential publication bias in the four meta-analyses, the overall RoB assessment generally suggesteda low risk of publication bias. Funnel plots asymmetries and significances of statistical tests for RoB were observed, butthe substantial evidence from the fail-safe N (for the correlation meta-analyses) and sensitivity analyses results reinforce the validity of the meta-analyses conducted in this review. Overall, the RoB assessment suggested that the results of the present meta-analyses are reliable.

### Practical Implications

The following potential applications highlight the usefulness of heart rate variability thresholds in clinical and exercise prescription settings:

- HRVTs have great potential for clinical and exercise prescription applications.
- Age, sex, weight class, training status and health status do not impact HRVT’s accuracy.
- Ergometer type, initial and incremental workload do not impact HRVT’s accuracy.
- The choice of outcome variable impacts HRVT’s determination and interpretation.
- Increment duration under 3 min is recommended for accurate HRVT2 determination.
- Frequency-domain and non-linear HRV indices yield better agreement and stronger correlation between HRVT2 and LT2-VT2 than time-domain HRV variables.

### Recommendations for Future Research

Further research in the field should:

- Report exact p-values for agreement and correlation analyses, as well as the Pearson correlation coefficient (r) and Bland-Altmann plots with limits of agreement, for each comparison between HRVT and LT-VT.
- Expand subject diversity by incorporating more women, patients, young and old subjects.
- Develop and assess algorithmic and more generally computed approaches for HRVTs determinations.
- Assess the test-retest reliability of HRVT determination in different settings and subjects.
- Conduct longitudinal studies to assess the predictive value of acute HRV responses to exercise or long-term adaptations in various populations and clinical settings.
- Investigate HRVTs determination when the upper body is involved (e.g., rowing, swimming, cross-country skiing, or ski-mountaineering).
- Use the STARD_HRV_ tool during the conceptualization stage to ensure that all items are considered, with particular attention to allow for a stabilization period prior to HRV sampling, to acknowledge whether breathing was controlled or not during HRV recording and to provide information about sample size determination. To this end, future studies could use the concordance and correlation values provided in this review to calculate the sample size required for their study (e.g. in the same way as [114]).

The seven recommendations reported above will improve the homogeneity and the scientific quality of the next publications in this field.

### Strengths and Limitations

The primary strength of this systematic review with meta-analysis is the exhaustiveness of the literature review carried out using a wide range of databases with search equations reviewed and corrected by an expert and adapted to each database. Moreover, and despite the strict inclusion criteria, the number of studies included in this review is relatively largecompared with previous reviews. Finally, the detailed and differentiated analysis of all main moderators that could impact HRVTs determination provides, for the first time, crucial information for future studies in this active research field.

According to the methodological quality assessment, the quality of the included studies should be improved to draw even more solid conclusions about the correlations and agreements between HRVTs and LT-VTs and the different moderators’ analyses conducted in this study. In addition, the comprehensive RoB analyses showed that a slight publication bias could not be ruled out for each of the four meta-analyses conducted in this review. Furthermore, most subjects were young, healthy men, which somewhat also limits the conclusions that can be drawn from this meta-analysis. Moreover, the moderators’ analyses hardly explained the heterogeneity in the four computed effect sizes.

There are also limitations to this study’s methodology and the choices made during its conceptualisation. Firstly, LT and VT were considered equivalent for the global effect sizes computations, although the agreement between ventilatory and LTs is still an ongoing debate. Secondly, the limits of agreement, which are frequently displayed in Bland-Altmann plots, were not analysed because they were available in less than half of the agreement analyses between HRVT and LT-VTs. Thirdly, since the HRVTs were determined using various outcomes expressed in different units, it was not possible to provide confidence intervals in the units of the corresponding outcomes. This would have made the reader’s assessment of the present results much easier. However, the standardised scales used to classify the SMD and Pearson’s r are adequate substitutes widely used in meta-analyses. Finally, due to clarity and sample size constraints, it was not possible to thoroughly evaluate each pair of exact HRVT and LT-VT determination methods separately. Indeed, because of the tremendous amount of HRVTs, LTs and VTs determination methods, this made impossible to create groups of sufficient size to assess the impact of the different HRV methods. As a result, the HRVT determination methods have been grouped by variable.

## Conclusion

Overall, HRV-derived thresholds (HRVT1 and HRVT2) showed trivial standardised mean differences and very strong correlation with their respective reference thresholds. However, ambiguous agreements were found when LTs and VTs were compared separately to HRVTs, suggesting that HRVT1 better agreed with LT1 and HRVT2 better agreed with VT2. Nevertheless, this systematic review with meta-analyses showed that subjects’ characteristics, ergometer, or initial and incremental workload had no impact on HRVTs determination and that straightforward, simple, and visual HRVTs determination methods yielded reliable results. In addition, frequency-domain and non-linear HRV indices, and short increment duration during graded exercise are better for HRVT2 determination. Considering the aforementioned conditions and limitations, the present results indicate that HRVTs might serve as surrogates for traditional reference thresholds when taken as a whole. However, it is essential to acknowledge the presence of heterogeneity across study results and differences in agreement when LTs and VTs are compared separately to HRVTs, underscoring the need for further research and development in this area, especially since HRVTs allowed non-invasive and cost-effective threshold determinations. The present findings contribute to the growing body of knowledge in the field, emphasizing the utility of HRVTs as promising and accessible tools for clinical and exercise prescription purposes.

## Electronic Supplementary Material

Below is the link to the electronic supplementary material.

**Supplementary Material 1:** Peer-reviewed search strategies

**Supplementary Material 2:** Full text screening exclusions

**Supplementary Material 3:** QUADAS-2

**Supplementary Material 4:** STARD HRV

**Supplementary Material 5:** HRVT1 moderator analyses

**Supplementary Material 6:** HRVT2 moderator analyses

## Data Availability

Data are available from the corresponding author upon reasonable request.

## Abbreviations

CI: Confidence Interval
DFA-α1: Detrended Fluctuation Analysis α1
ECG: Electrocardiogram
fHF: Frequency peak of the HF band
Fisher’s Z: Normally distributed Fisher transformation of the Pearson correlation coefficient
HF: High-Frequency spectral power
HRV: Heart Rate Variability
HRVT1/2: Heart Rate Variability Threshold 1/2
I^2^: Proportion of variance between studies attributed to true variation in effect sizes
LF: Low Frequency spectral power
LT1/2: Lactate Threshold 1/2
METs: Metabolic Equivalent of Task (a measure of exercise intensity)
LT1/2: Lactate Threshold 1/2
Power: Exercise power output
LT1/2: Lactate Threshold 1/2
Pearson’s r: Pearson correlation coefficient
Q-test: Cochrane Q-test for heterogeneity significance
RoB: Risk of Bias
RQA: Recurrence Quantification Analysis
RMSSD: Root Mean Square of Successive Differences
RSA: Respiratory Sinus Arrhythmia
SDNN: Standard Deviation of NN intervals
SD1/2: Poincaré plot Standard Deviation 1/2
SMD: Standardized Mean Difference
VT1/VT2: Ventilatory Threshold 1/2
