# Supplementary material for: Agreement Between Heart Rate Variability - Derived vs. Ventilatory and Lactate Thresholds: A Systematic Review with Meta-Analyses
Source: Sports Med Open. 2024 Oct 8;10:109. doi: 10.1186/s40798-024-00768-8 (PMC11461412; doi:10.1186/s40798-024-00768-8)
Supplement: Supplementary file 5 — Supplementary Material 5: HRVT1 moderator analyses [file 40798_2024_768_MOESM5_ESM.pdf]

Agreement between heart rate variability - derived vs. ventilatory and lactate thresholds: A systematic review with meta-analyses

Electronic Supplementary Material 5

HRVT1 Moderator analyses

Sports Medicine - Open

Valérian Tanner[1]*, Grégoire P. Millet[1], Nicolas Bourdillon[1]

[1]Institute of Sport Sciences, University of Lausanne, Lausanne, Switzerland.

*Corresponding author:

- E-mail: valerian.tanner@unil.ch
- Adress: Quartier UNIL-Centre, Bâtiment Synathlon, 1015 Lausanne, Switzerland.

Subjects characteristics **3**
Age: 3
Gender: 4
Weight class: 5
Training status: 6
Health status: 7
Pathology: 8
HRVT1 and LT1-VT1 determination methods **9**
Reference thresholds: 9
Reference threshold determination type: 10
HRV domains: 11
HRV variables: 12
Number of HRV variables for HRVT determination: 13
HRVT1 determination type: 14
HRVT1 determination complexity: 15
HRV recording devices: 16
HRV recording device type: 17
HRV Softwares: 18
Study protocol **19**
Outcomes: 19
Outcomes formats: 21
Ergometers: 22
Initial workload: 23
Increment workload (METs) : 24
Increment workload (%) : 25
Increment duration: 26
Continent: 27

Subjects characteristics

Age:

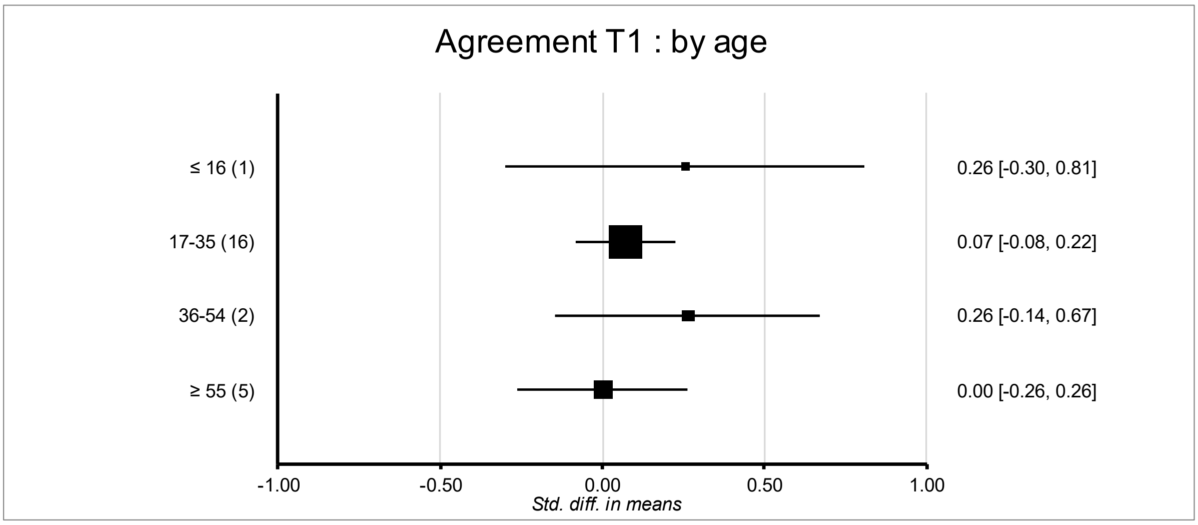

Between subgroups: p = 0.678

There is no difference in std. diff. in means between subgroups.

	Heterogeneity	
	P-value	***I-squared (%)***
≤ 16	1	0.0
17-35	0.000	90.6
36-54	0.000	92.2
≥ 55	0.014	68.1

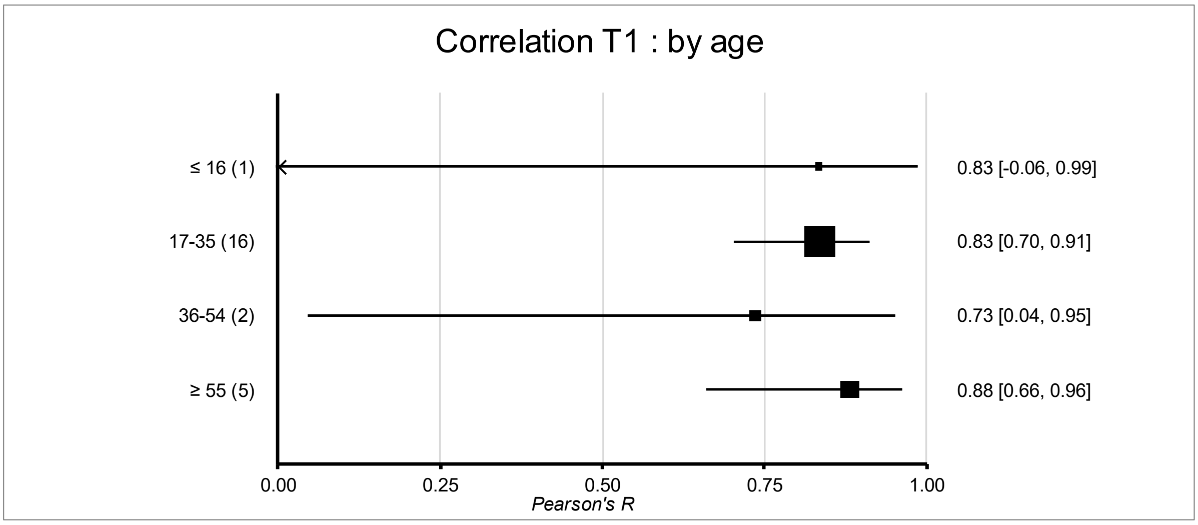

Between subgroups: p = 0.879

There is no difference in Pearson's R between subgroups.

	Heterogeneity	
	P-value	***I-squared (%)***
≤ 16	1	0.0
17-35	0.000	93.3
36-54	0.036	77.2
≥ 55	0.000	86.4

Gender:

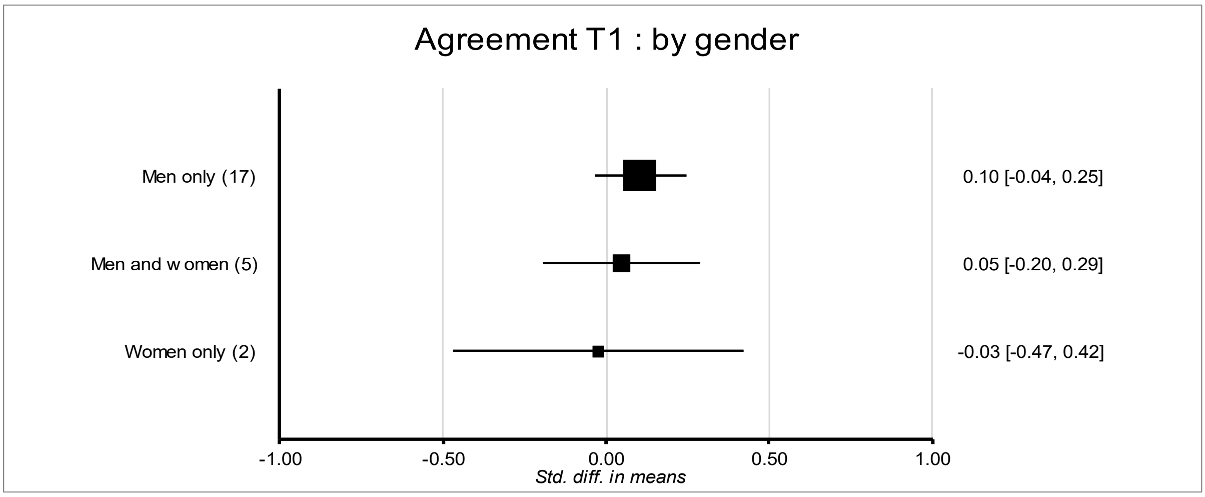

Between subgroups: p = 0.820

There is no difference in std. diff. in means between subgroups.

	Heterogeneity	
	P-value	***I-squared (%)***
Men only	0.000	90.7
Men and women	0.003	74.5
Women only	0.001	90.9

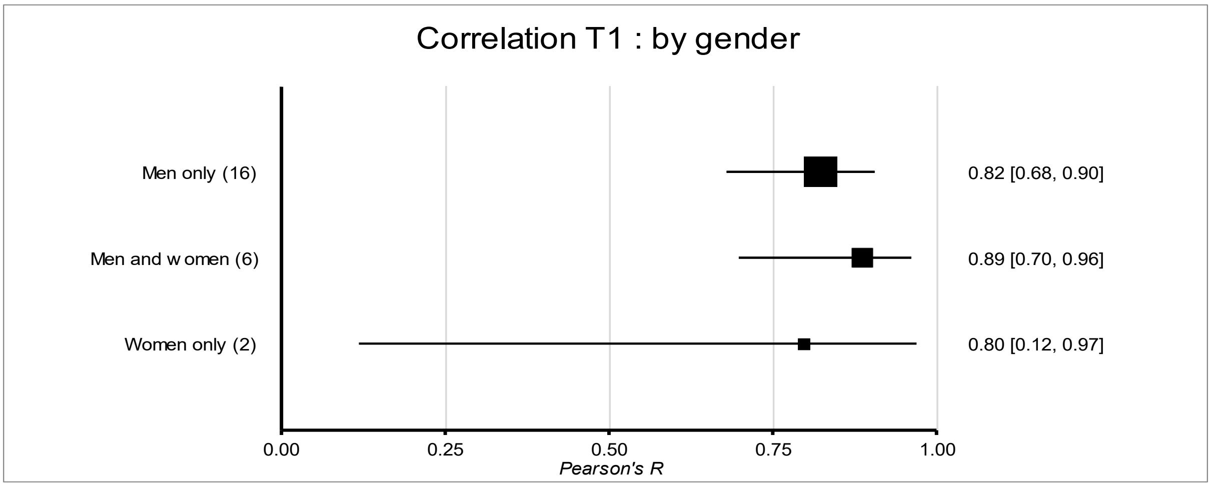

Between subgroups: p = 0.732

There is no difference in Pearson's R between subgroups.

	Heterogeneity	
	P-value	***I-squared (%)***
Men only	0.000	83.5
Men and women	0.000	97.5
Women only	0.446	0.0

Weight class:

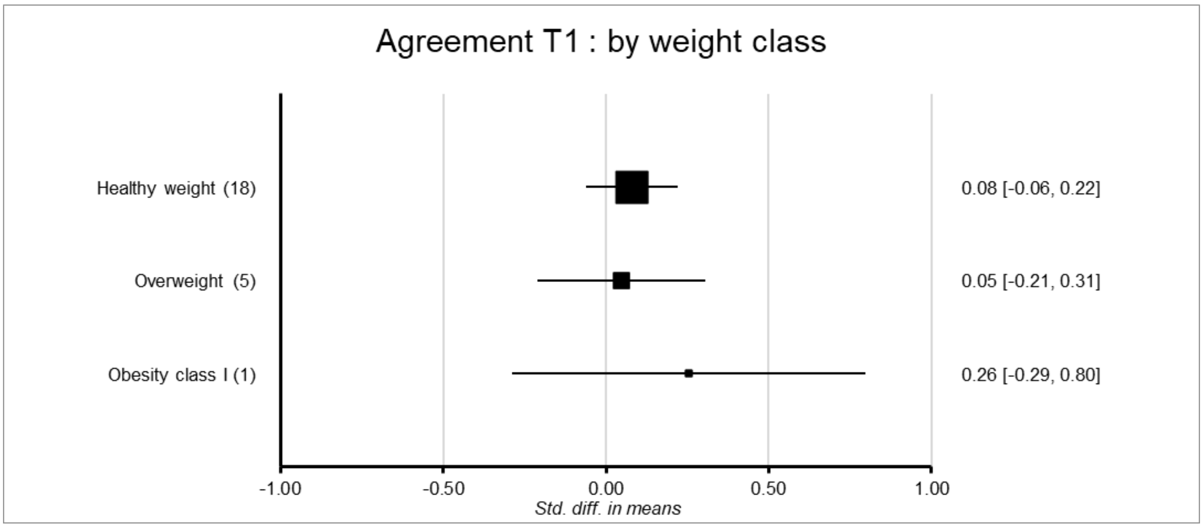

Healthy weight, BMI 18.5 – 25; Overweight, BMI 25 – 30; Obesity class I, BMI 30 – 35.

Between subgroups: p = 0.796

There is no difference in std. diff. in means between subgroups.

	**Heterogeneity**	
	P-value	***I-squared (%)***
Healthy weight	0.000	89.6
Overweight	0.000	84.5
Obesity class I	1	0.0

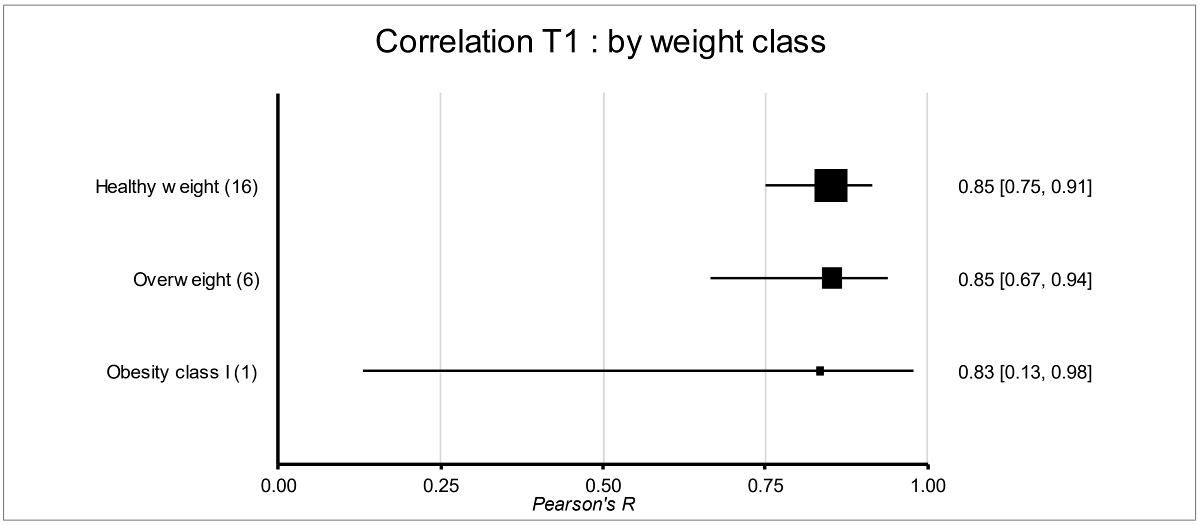

Healthy weight, BMI 18.5 – 25; Overweight, BMI 25 – 30; Obesity class I, BMI 30 – 35.

Between subgroups: p = 0.994

There is no difference in Pearson's R between subgroups.

	**Heterogeneity**	
	P-value	***I-squared (%)***
Healthy weight	0.000	85.9
Overweight	0.000	89.8
Obesity class I	1	0.0

Training status:

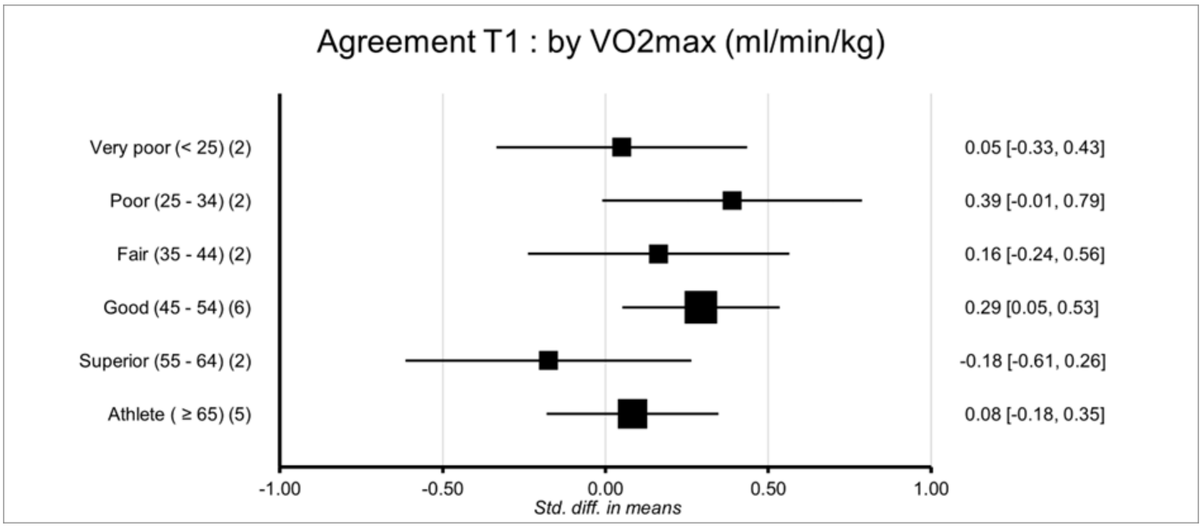

Between subgroups: p = 0.377

There is no difference in std. diff. in means between subgroups.

	Heterogeneity	
	P-value	*I-squared (%)*
Very poor (< 25)	0.130	56.4
Poor (25 - 34)	0.036	77.4
Fair (35 - 44)	0.024	80.3
Good (45 - 54)	0.000	94.6
Superior (55 - 64)	0.010	84.7
Athlete (≥ 65)	0.028	63.2

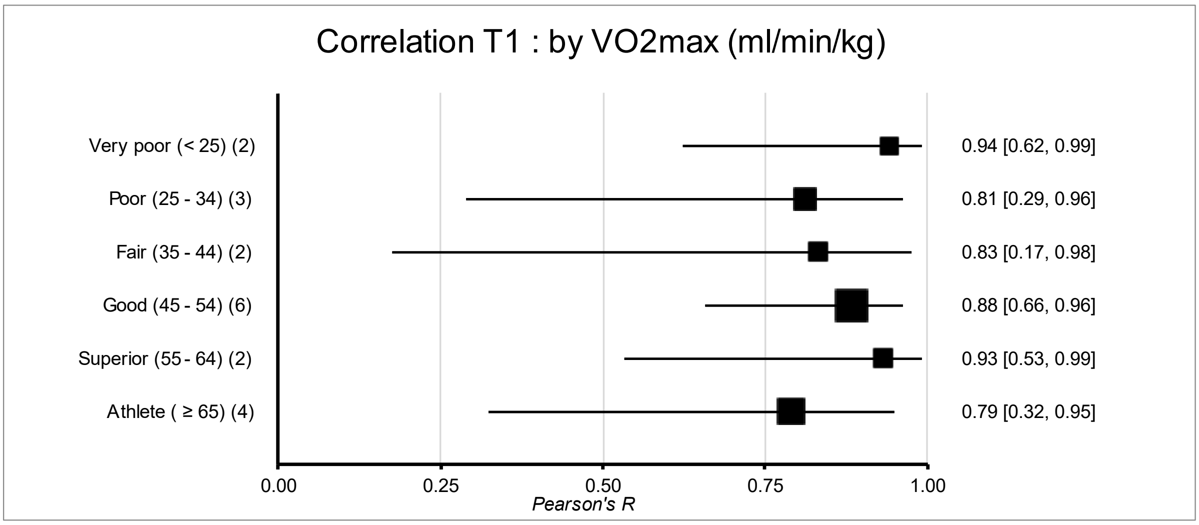

Between subgroups: p = 0.872

There is no difference in Pearson's R between subgroups.

	Heterogeneity	
	P-value	*I-squared (%)*
Very poor (< 25)	0.000	93.5
Poor (25 - 34)	0.008	79.2
Fair (35 - 44)	0.748	0.0
Good (45 - 54)	0.000	96.9
Superior (55 - 64)	0.001	90.6
Athlete (≥ 65)	0.026	67.7

Health status:

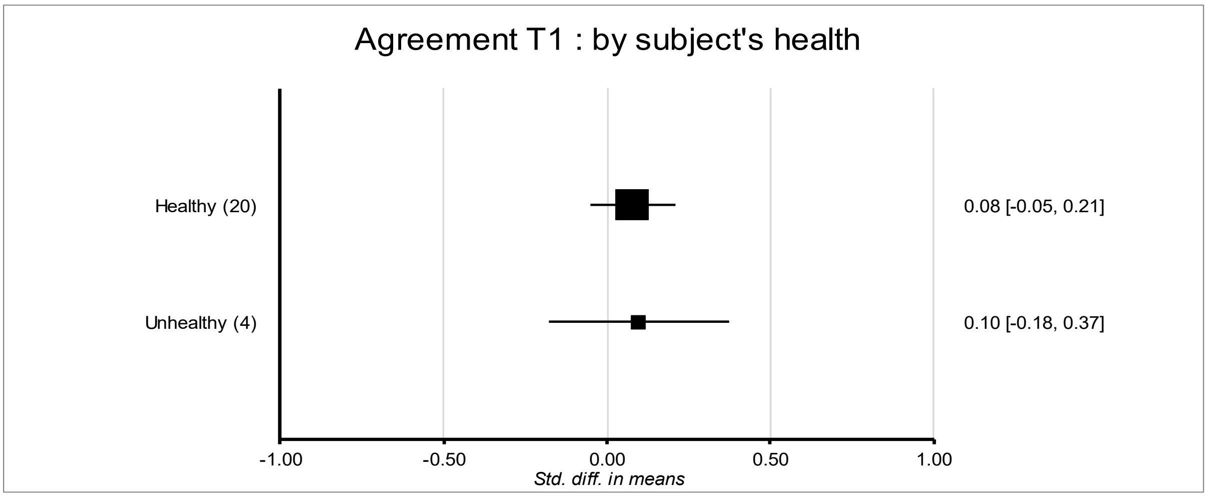

Between subgroups: p = 0.907

There is no difference in std. diff. in means between subgroups.

	Heterogeneity	
	P-value	*I-squared (%)*
Healthy	0.000	89.1
Unhealthy	0.000	87.5

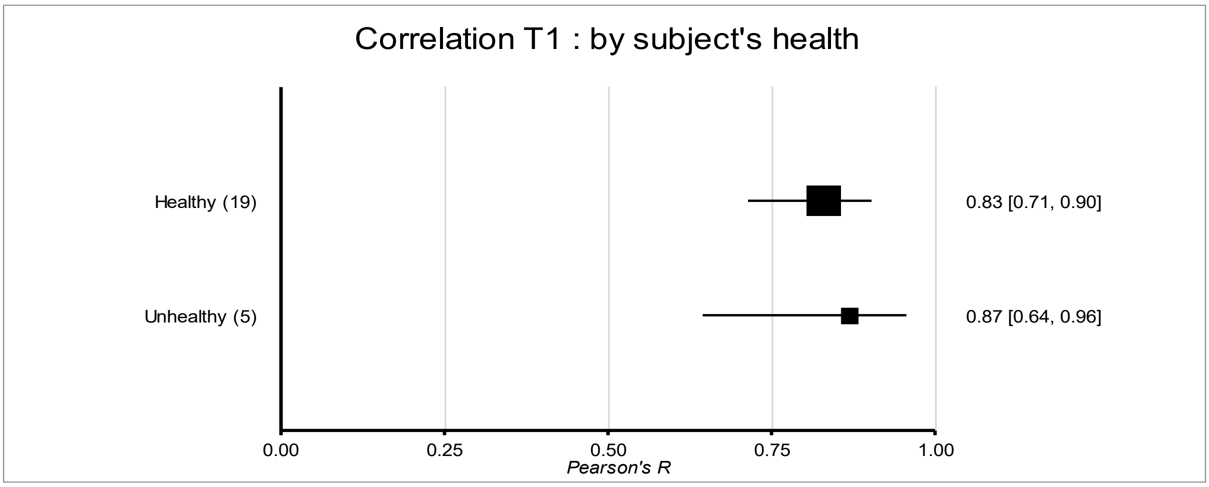

Between subgroups: p = 0.662

There is no difference in Pearson's R between subgroups.

	Heterogeneity	
	P-value	*I-squared (%)*
Healthy	0.000	92.6
Unhealthy	0.000	91.6

Pathology:

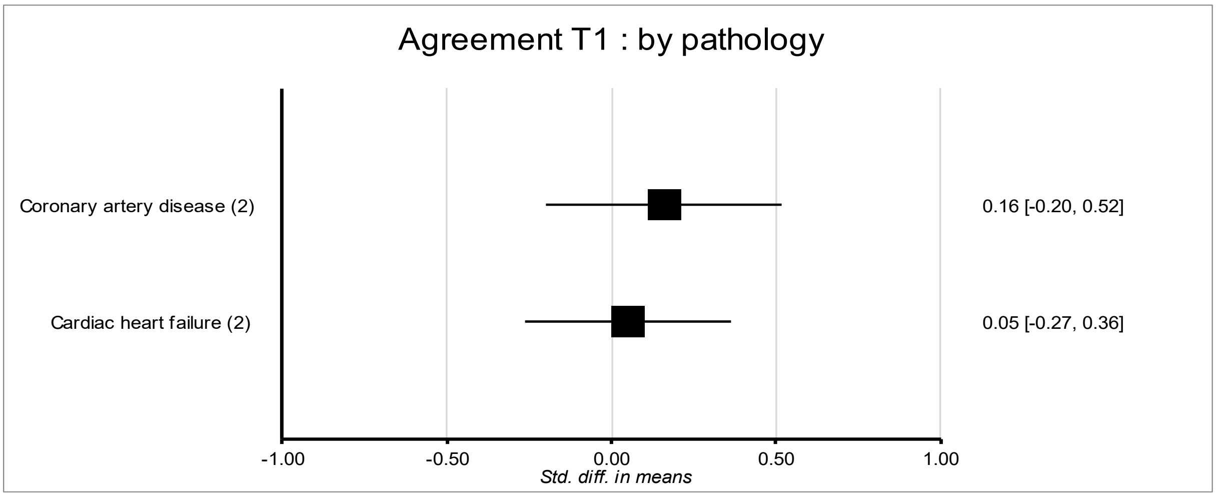

Between subgroups: p = 0.652

There is no difference in std. diff. in means between subgroups.

	Heterogeneity	
	P-value	*I-squared (%)*
Coronary artery disease	0.000	94.3
Chronic heart failure	0.130	56.4

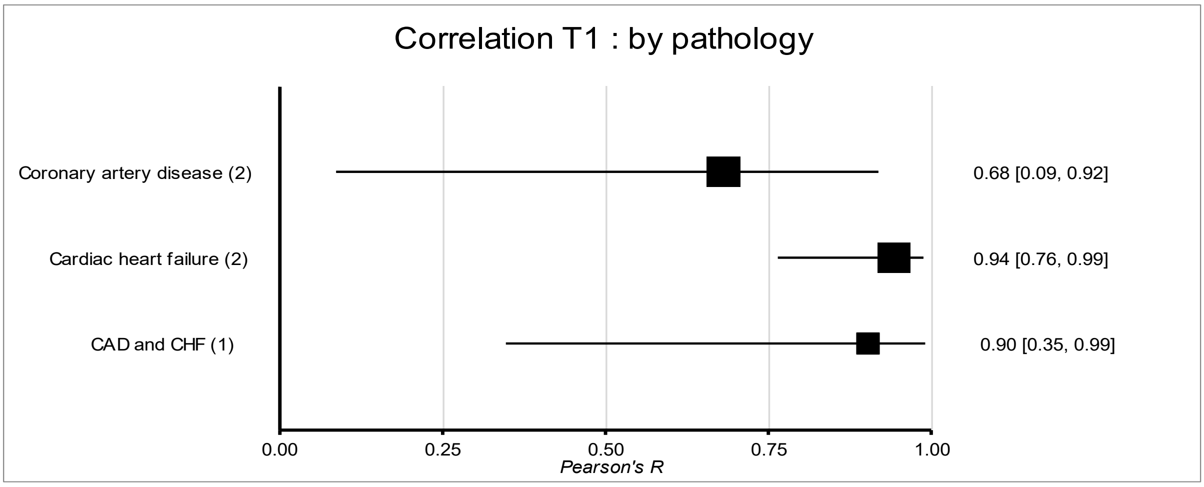

Between subgroups: p = 0.219

There is no difference in Pearson's R between subgroups.

	Heterogeneity	
	P-value	*I-squared (%)*
CAD	0.442	0.0
CHF	0.000	93.5
CAD and CHF	1	0.0

HRVT1 and LT1-VT1 determination methods

Reference thresholds:

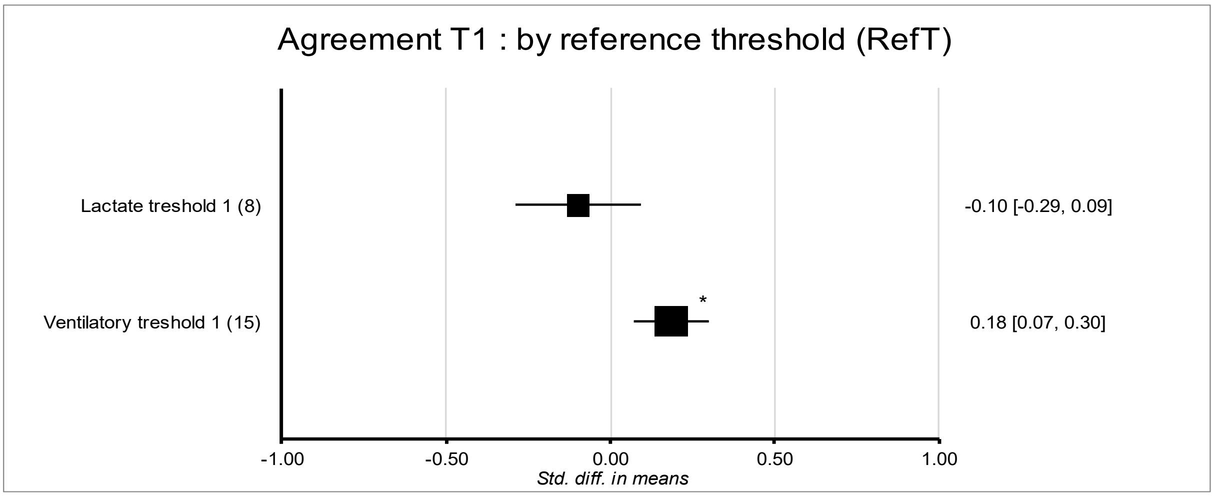

**p < 0.05 for difference with Lactate threshold 1*

Between subgroups: **p = 0.012**

The std. diff. in means are different between subgroups.

	Heterogeneity	
	P-value	***I-squared (%)***
Lactate threshold 1	0.000	90.6
Ventilatory threshold 1	0.000	76.7

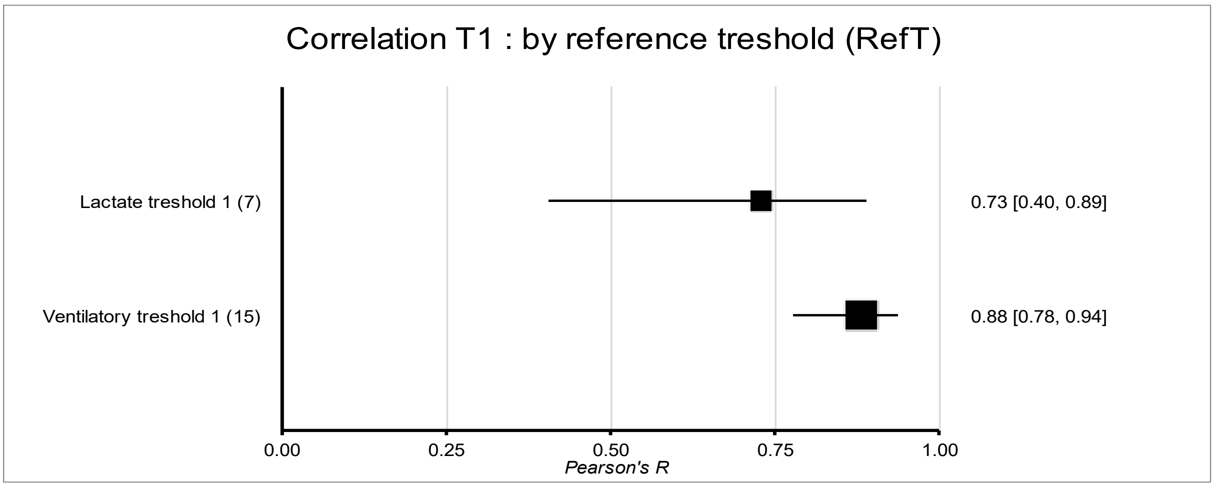

Between subgroups: p = 0.139

There is no difference in Pearson's R between subgroups.

	Heterogeneity	
	P-value	***I-squared (%)***
Lactate threshold 1	0.008	65.5
Ventilatory threshold 1	0.000	95.1

Reference threshold determination type:

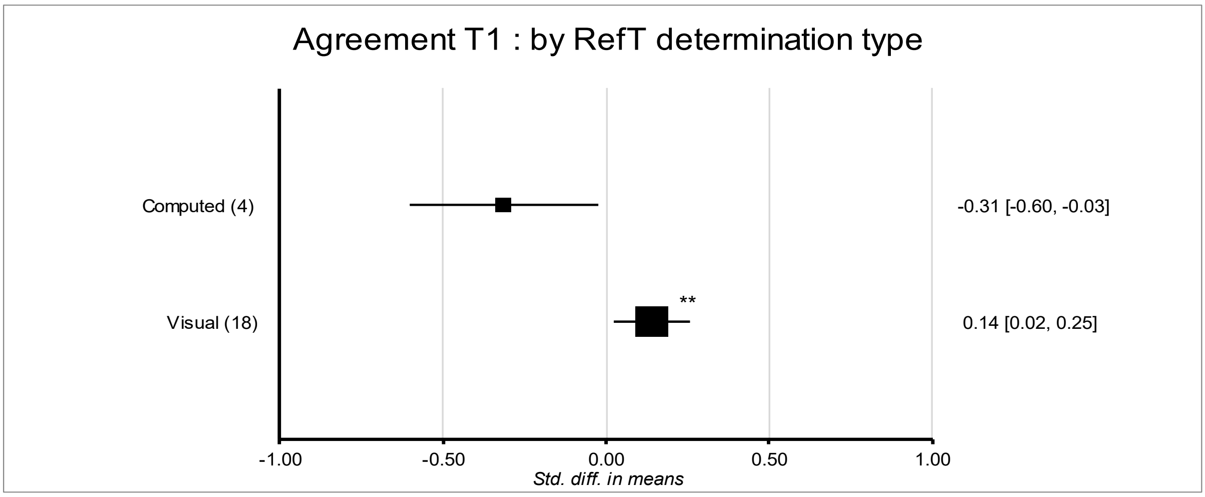

***p < 0.01 for difference with Computed*

Between subgroups: **p = 0.004**

The std. diff. in means are different between subgroups.

	Heterogeneity	
	P-value	*I-squared (%)*
Computed	0.000	86.3
Visual	0.000	88.1

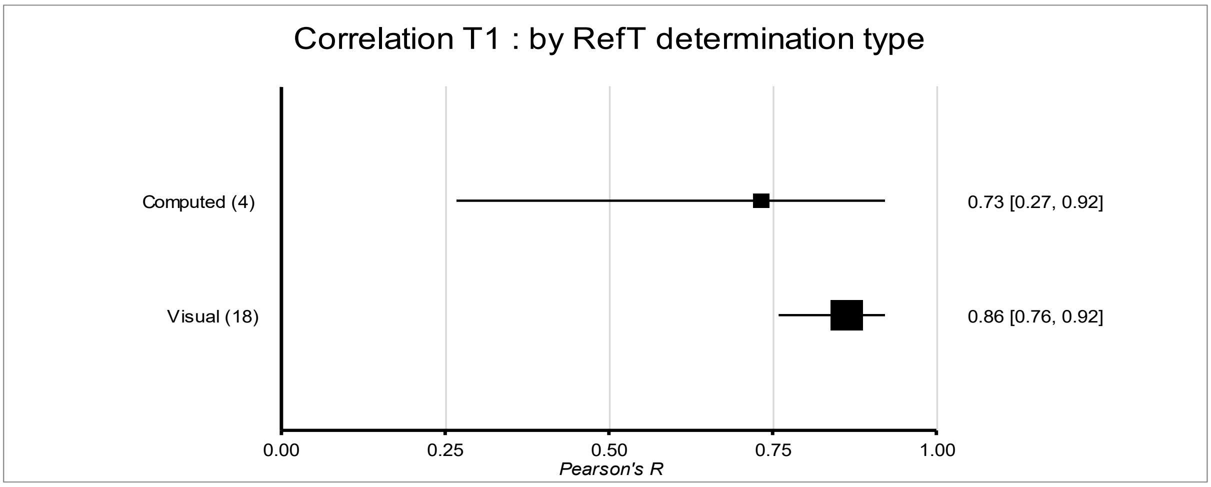

Between subgroups: p = 0.326

There is no difference in Pearson's R between subgroups.

	Heterogeneity	
	P-value	*I-squared (%)*
Computed	0.009	74.2
Visual	0.000	94.2

HRV domains:

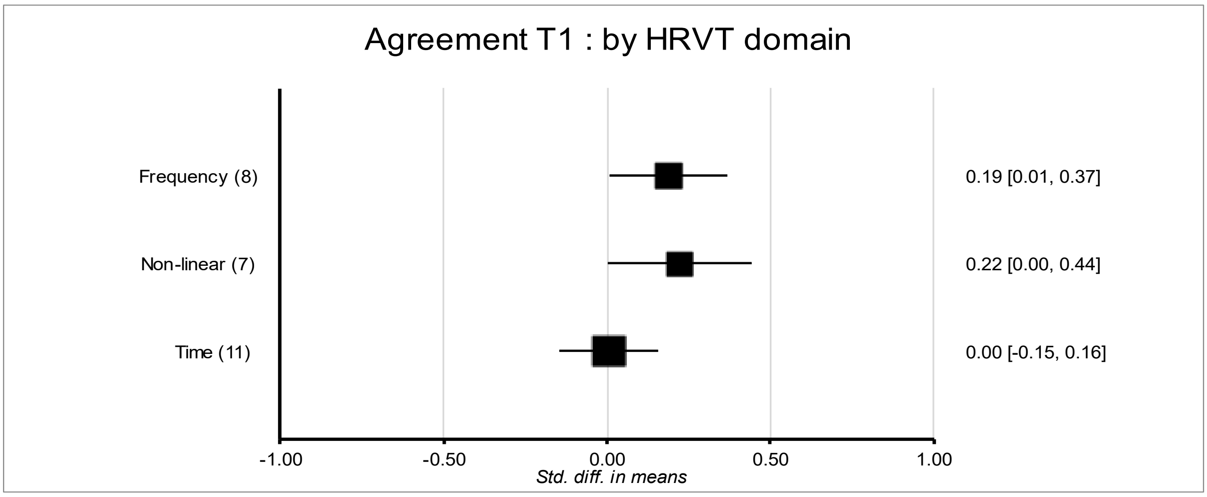

HRVT, heart rate variability threshold.

Between subgroups: p = 0.169

There is no difference in std. diff. in means between subgroups.

	**Heterogeneity**	
	P-value	***I-squared (%)***
Frequency	0.019	46.5
Non-linear	0.000	93.2
Time	0.000	84.1

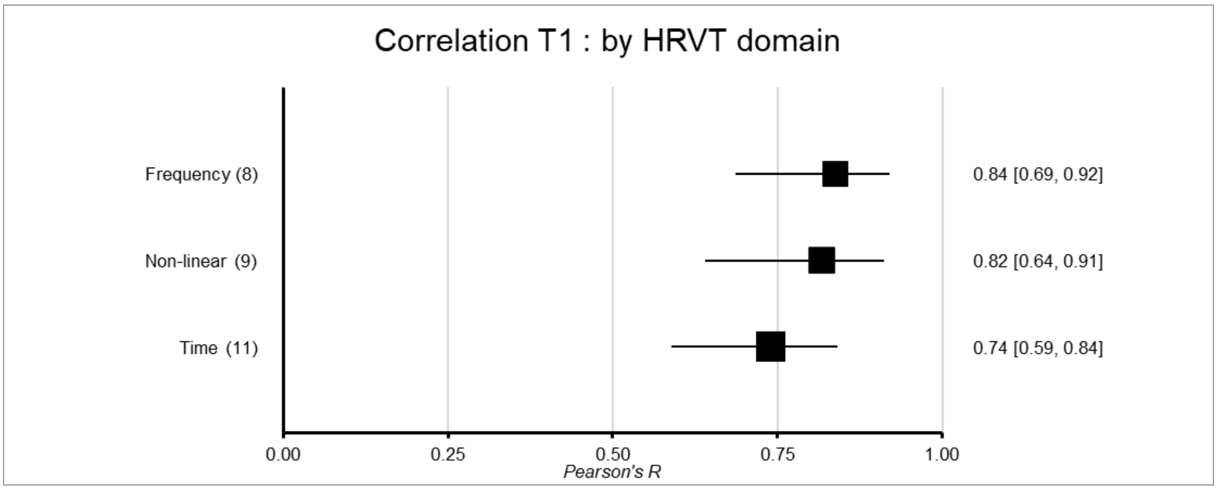

HRVT, heart rate variability threshold

Between subgroups: p = 0.476

There is no difference in Pearson's R between subgroups.

	**Heterogeneity**	
	P-value	***I-squared (%)***
Frequency	0.000	93.5
Non-linear	0.000	83.6
Time	0.000	79.4

HRV variables:

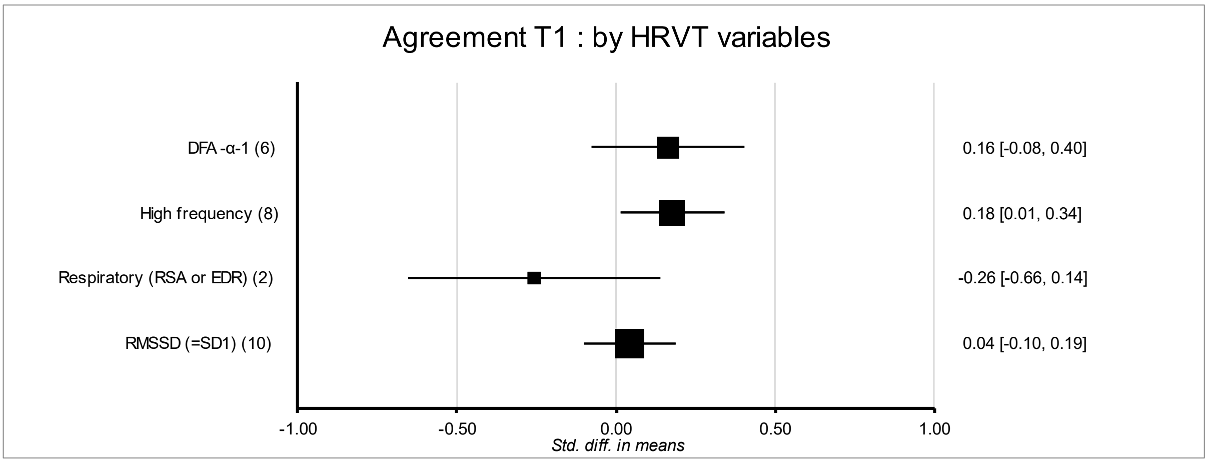

HRVT, heart rate variability threshold.

Between subgroups: p = 0.189

There is no difference in std. diff. in means between subgroups.

	Heterogeneity	
	P-value	*I-squared (%)*
DFA-α-1	0	79.1
High frequency	0.009	50.4
Respiratory (RSA or EDR)	0	90.3
RMSSD (=SD1)	0.000	84.3

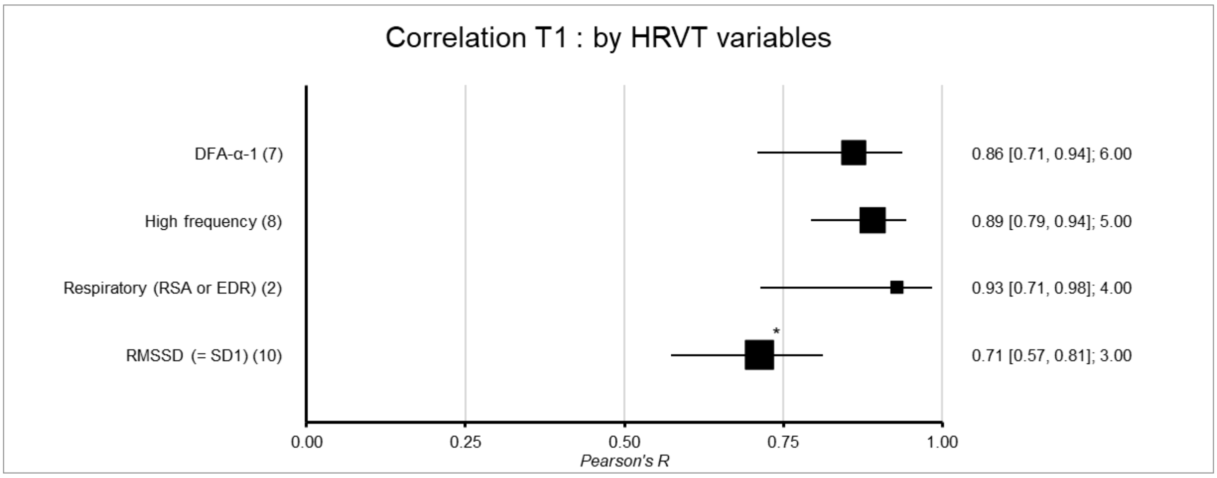

**<* 0.05 for difference with High frequency.*

Between groups: **p = 0.034**

RMSSD (= SD1) Pearson's R lower than:

- High frequency: p = 0.013

There is no other difference in Pearson's R between subgroups.

	Heterogeneity	
	P-value	*I-squared (%)*
DFA-α-1	0.000	82.7
High frequency	0.000	85.3
Respiratory (RSA or EDR)	0.000	92.0
RMSSD (= SD1)	0.000	75.7

Number of HRV variables for HRVT determination:

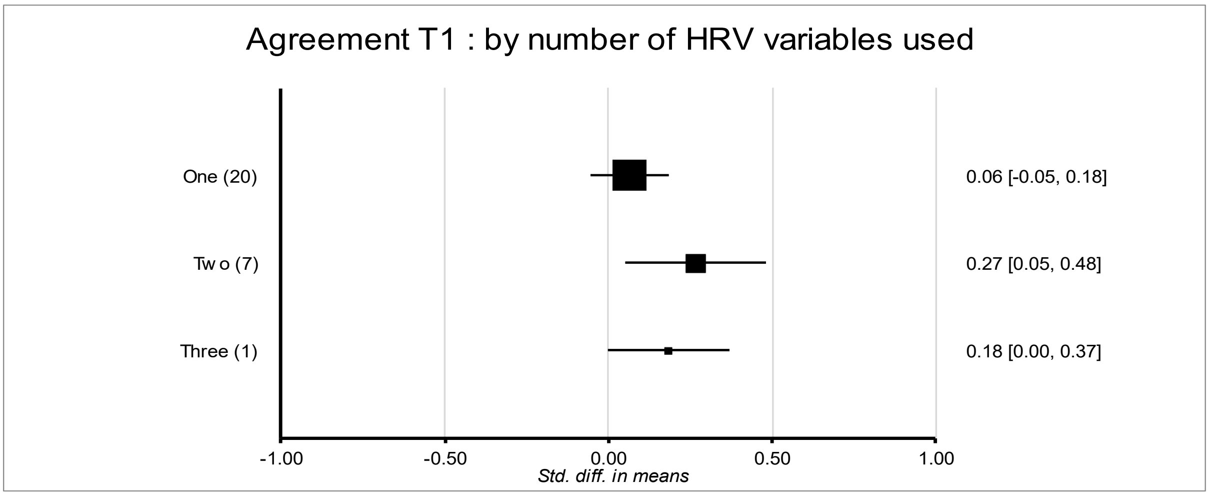

Between subgroups: p = 0.265

There is no difference in std. diff. in means between subgroups.

	Heterogeneity	
	P-value	***I-squared (%)***
One	0.000	86.3
Two	0.026	50.9
Three	1	0.0

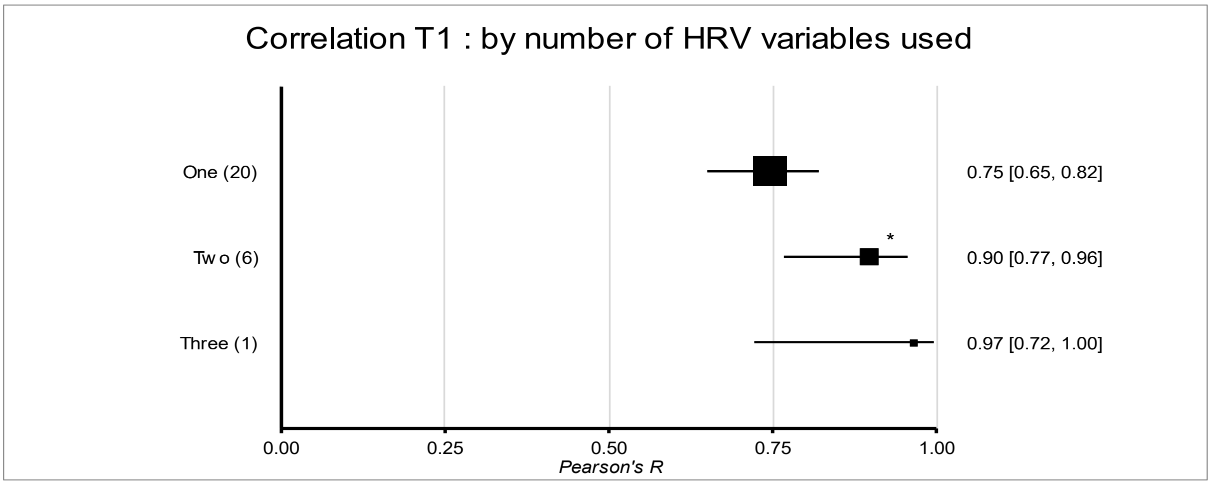

**p < 0.05 for difference with One*

Between subgroups: **p = 0.032**

One Pearson's R is higher than:

- Two: p = 0.046

There is no other difference in Pearson's R between subgroups.

	Heterogeneity	
	P-value	***I-squared (%)***
One	0.002	71.3
Two	0.000	85.2
Three	1	0.0

HRVT1 determination type:

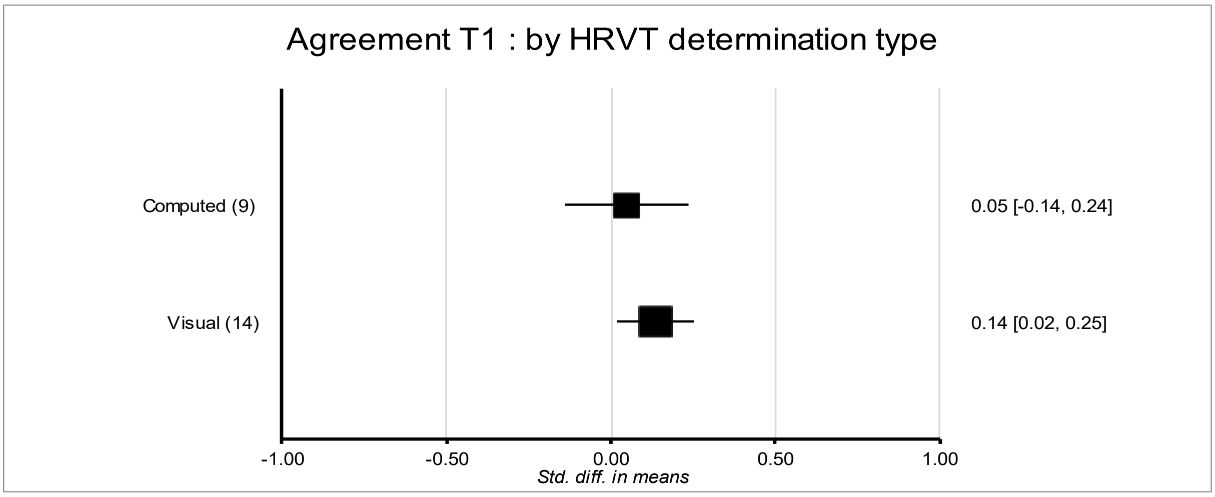

HRVT, heart rate variability threshold.

Between subgroups overall : p = 0.437

There is no difference in std. diff. in means between subgroups.

	Heterogeneity	
	P-value	***I-squared (%)***
Computed	0.000	92.7
Visual	0.000	72.2

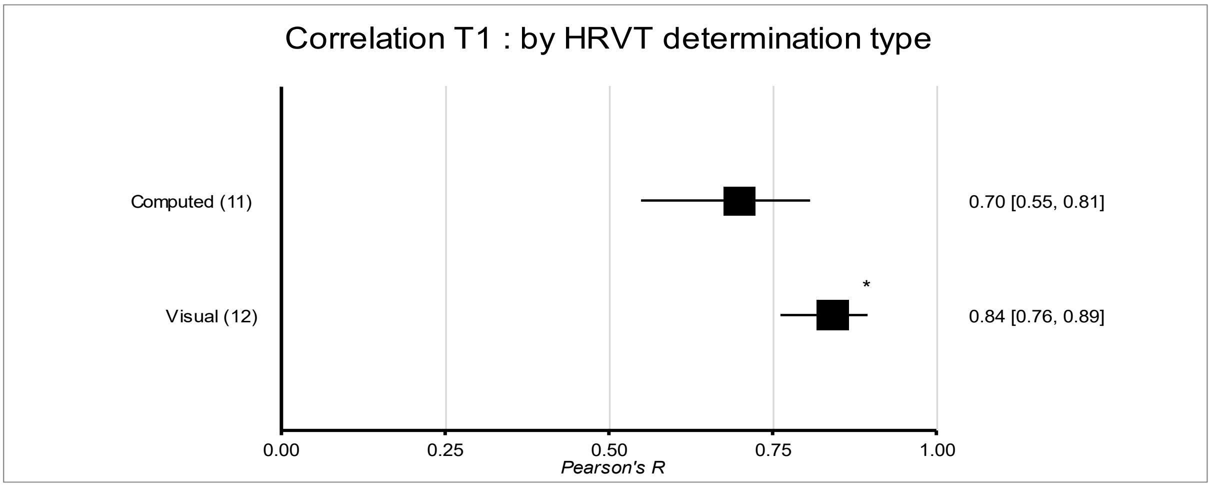

**p < 0.05 for difference with Computed. HRVT, heart rate variability threshold.*

Between subgroups overall : **p = 0.038**

The Pearson's R is different between subgroups.

	Heterogeneity	
	P-value	***I-squared (%)***
Computed	0.000	85.4
Visual	0.000	80.9

HRVT1 determination complexity:

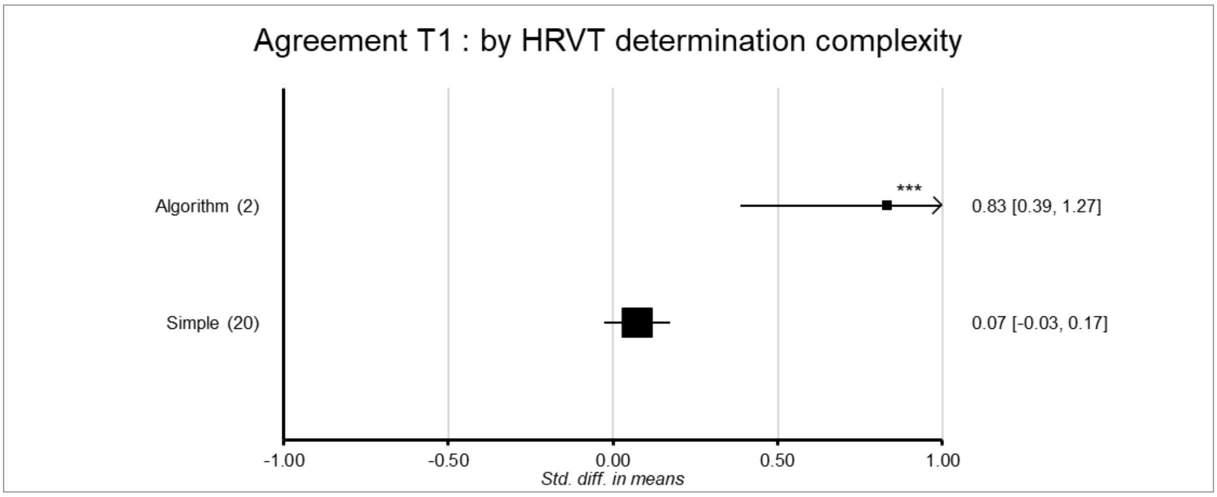

****p < 0.01 for difference with Simple. HRVT, heart rate variability threshold. Simple: HRVT determination made visually or, if calculated, by applying a fixed threshold to an HRV variable. Algorithm: HRVT determination requiring a more complex algorithm.*

Between subgroups: **p < 0.001**

The std. diff. in means are different between subgroups.

	Heterogeneity	
	P-value	*I-squared (%)*
Algorithm	0.000	98.5
Simple	0.000	78.5

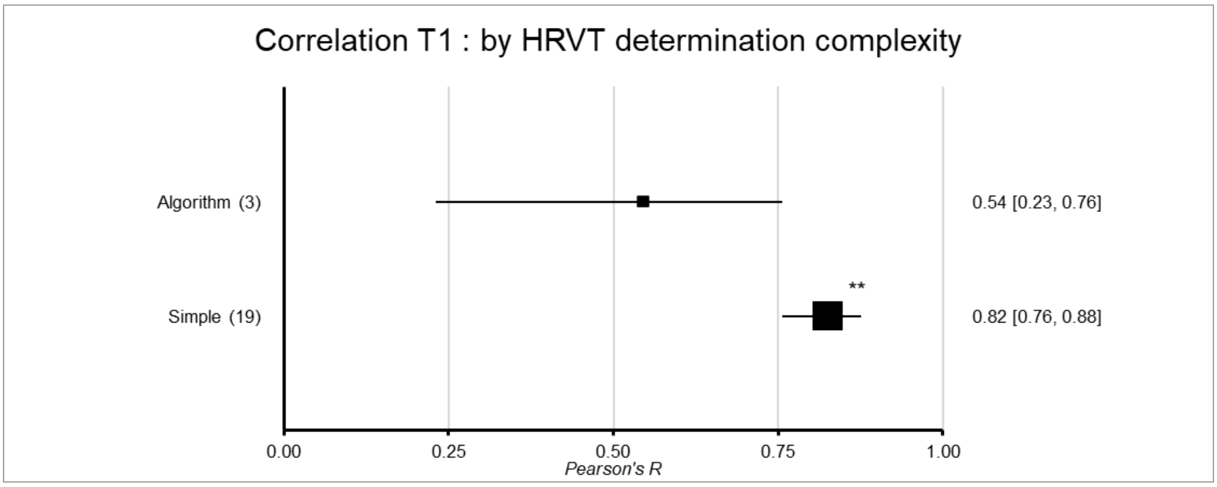

***p < 0.01 for difference with Algorithm. Simple: HRVT determination made visually or, if calculated, by applying a fixed threshold to an HRV variable. Algorithm: HRVT determination requiring a more complex algorithm.*

Between subgroups: **p = 0.009**

The Pearson's R is different between subgroups.

	Heterogeneity	
	P-value	*I-squared (%)*
Algorithm	0.000	92.4
Simple	0.000	77.2

HRV recording devices:

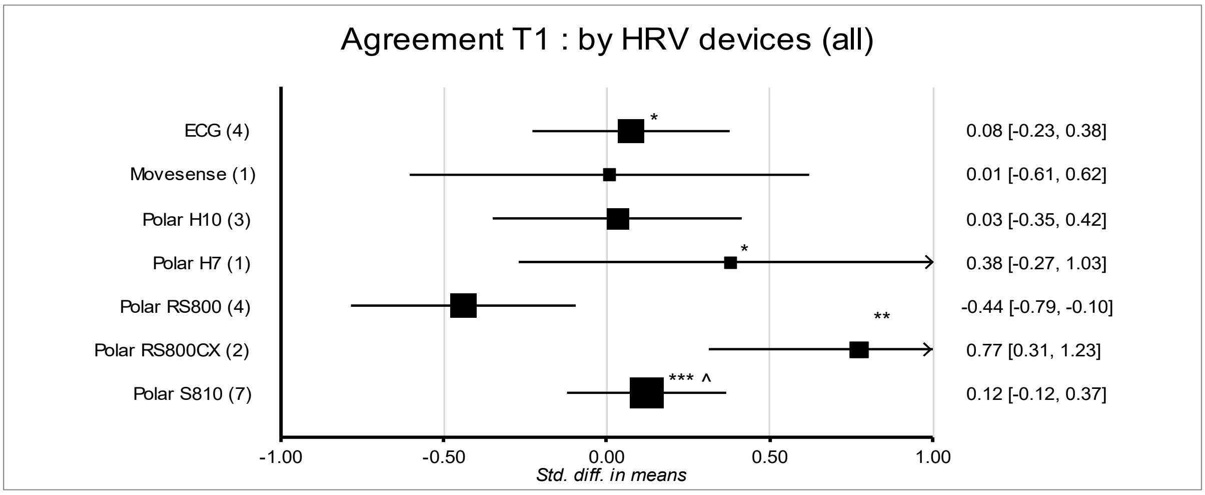

**p < 0.05, **p < 0.01 and ***p < 0.001 for difference with PolarRS800; ^p < 0.05 for difference with PolarS810*

Between subgroups: **p = 0.005**

PolarRS800 std. diff. in means is lower than:

- ECG: p = 0.028
- Polar H7 p = 0.029
- Polar S810: p = 0.009
- Polar RS800CX: p < 0.000

PolarS810 std. diff. in means is lower than:

- Polar RS800CX: p = 0.014

	**Heterogeneity**	
	P-value	***I-squared (%)***
ECG	0.000	74.8
Movesense	1	0.0
Polar H10	0.001	75.9
Polar H7	1	0.0
Polar RS800	0.000	90.1
PolarRS800CX	0.000	92.5
Polar S810	0.000	79.4

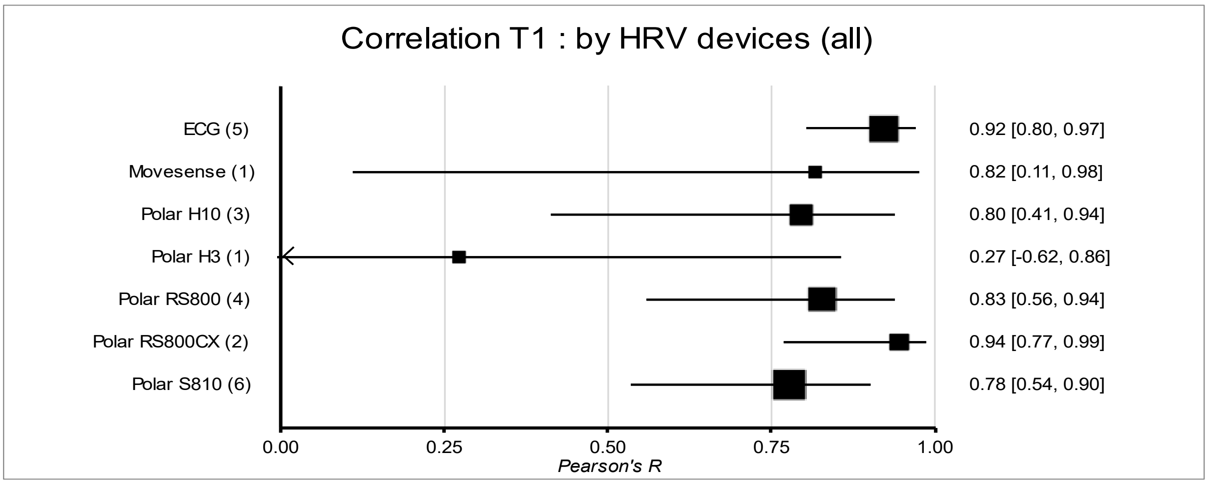

Between subgroups: p = 0.199

There is no difference in Pearson's R between subgroups.

	**Heterogeneity**	
	P-value	***I-squared (%)***
ECG	0.000	90.8
Movesense	1	0.0
Polar H10	0.007	79.7
Polar H3	1	0.0
Polar RS800	0.000	89.0
Polar RS800CX	0.001	90.5
Polar S810	0.000	78.7

HRV recording device type:

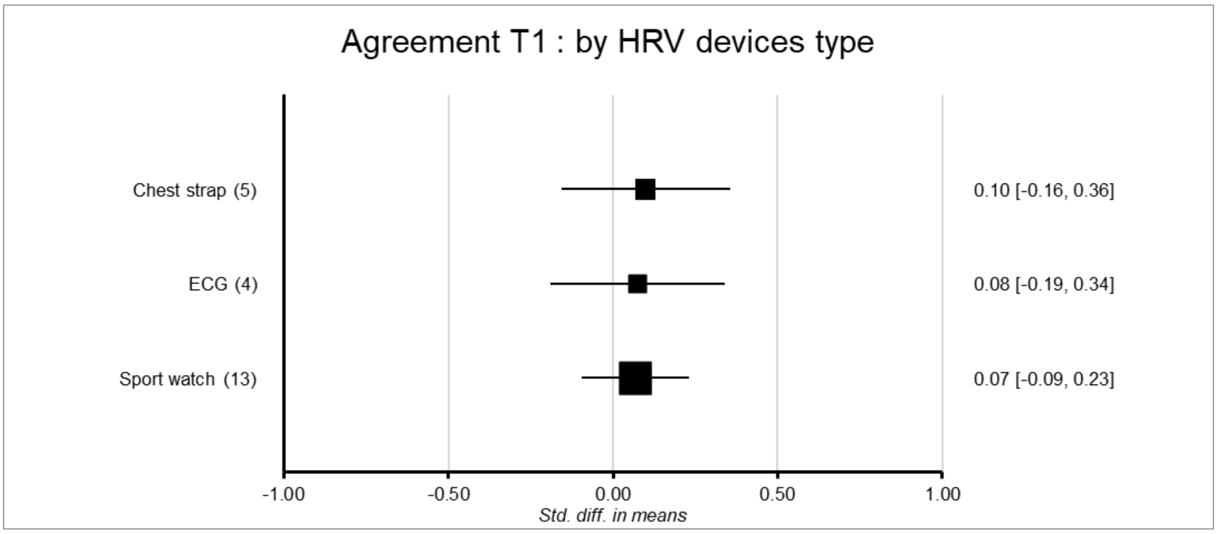

Between subgroups: p = 0.980

There is no difference in std. diff. in means between subgroups.

	Heterogeneity	
	P-value	***I-squared (%)***
Chest strap	0.009	70.1
ECG	0.026	67.5
Sport watch	0.000	92.8

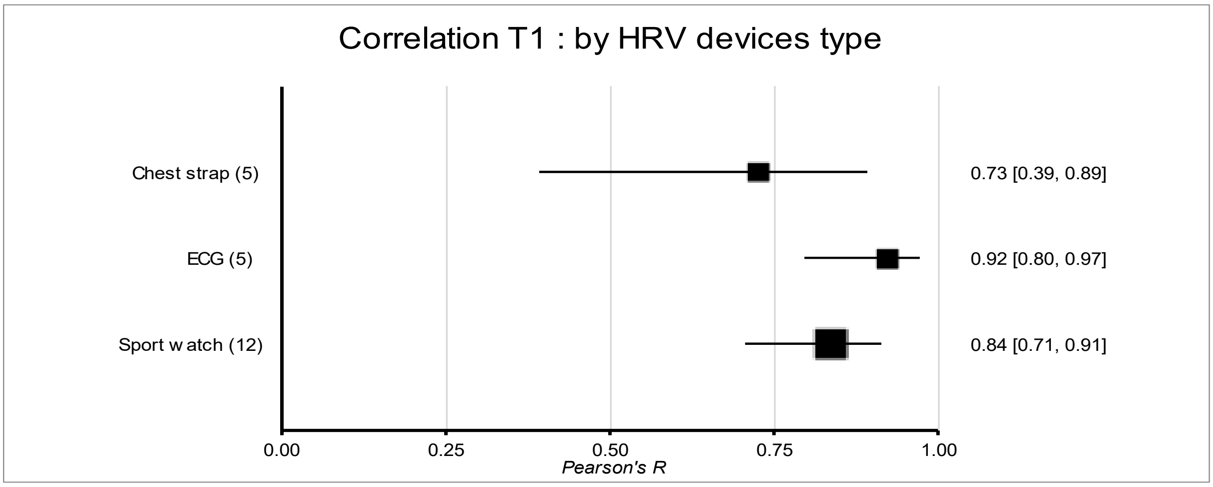

Between subgroups: p = 0.175

There is no difference in Pearson's R between subgroups.

	Heterogeneity	
	P-value	***I-squared (%)***
Chest strap	0.000	92.5
ECG	0.000	90.8
Sport watch	0.000	87.9

HRV Softwares:

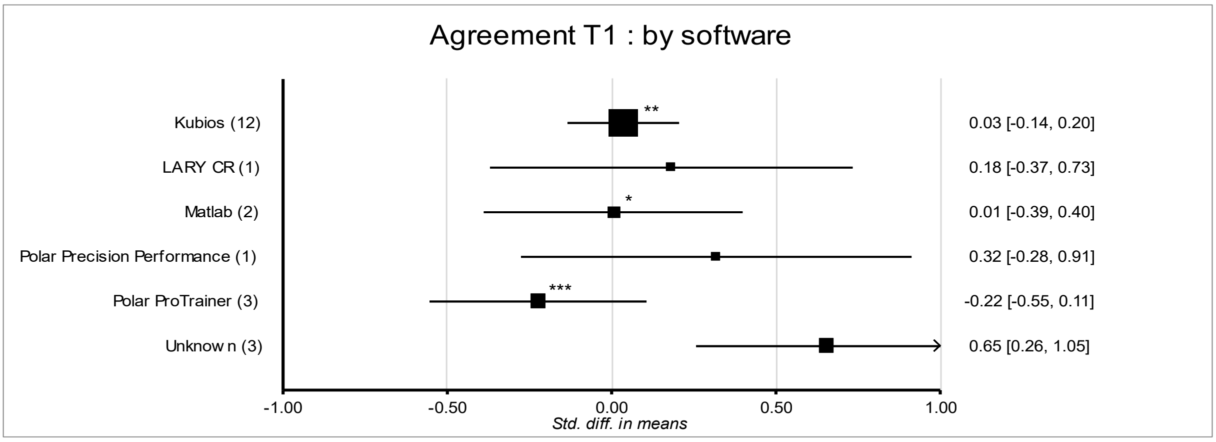

**p < 0.05, **p < 0.01 and ***p < 0.001 for difference with Unknown*

Between subgroups: **p = 0.029**

Unknown std. diff. in means is higher than:

- Kubios: p = 0.005
- Matlab: p = 0.023
- Polar ProTrainer: p < 0.001

There is no other difference in std. diff. in means between subgroups.

	Heterogeneity	
	P-value	***I-squared (%)***
Kubios	0.000	78.61
LARY_CR	1	0.00
Matlab	0.568	0.00
Polar Precision Performance	1	0.00
Polar ProTrainer	0.000	93.36
Unknown	0.000	97.41

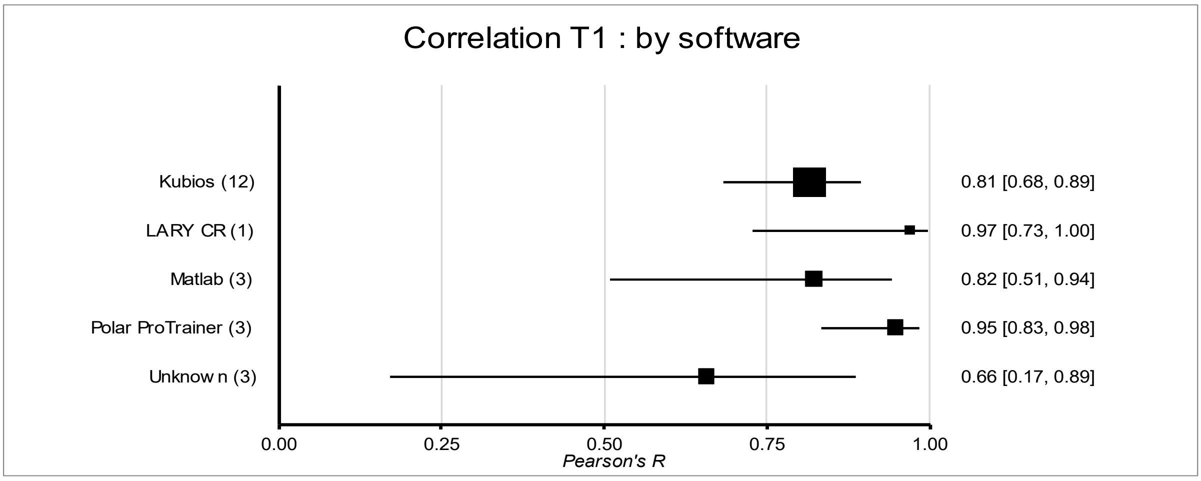

Between subgroups: p = 0.093

There is no difference in Pearson's R between subgroups.

	Heterogeneity	
	P-value	***I-squared (%)***
Kubios	0.015	76.33
LARY CR	1	0.00
Matlab	0.000	74.71
Polar ProTrainer	0.000	97.26
Unknown	0.000	90.30

Study protocol

Outcomes:

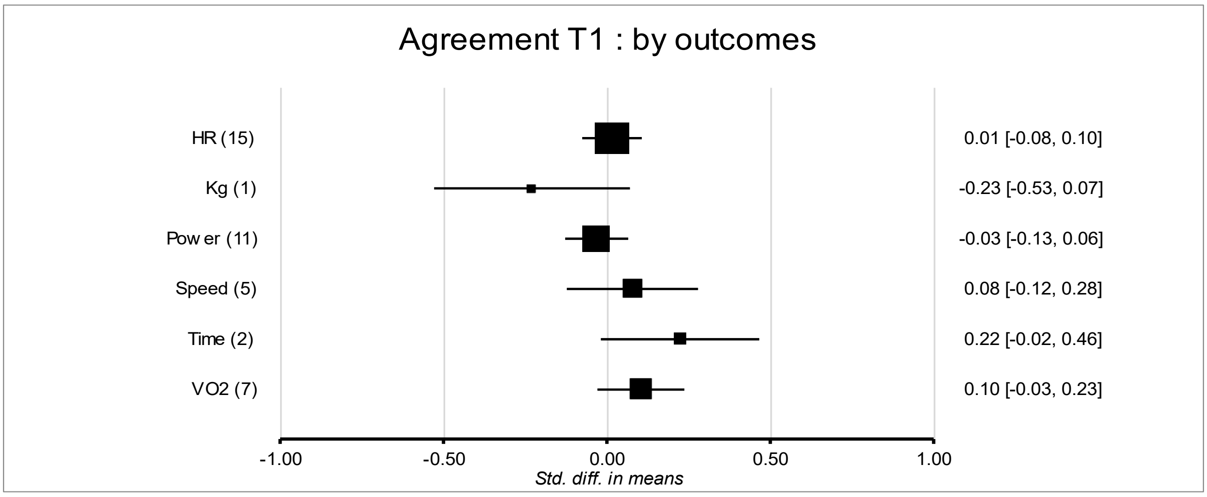

Between subgroups: p = 0.133

There is no difference in std. diff. in means between subgroups.

	Heterogeneity	
	P-value	***I-squared (%)***
HR	0.000	83.2
Kg	1	0.0
Power	0.000	93.5
Speed	0.000	79.4
Time	0.101	40.0
VO2	0.040	45.0

Correlation T1 by outcomes on the next page

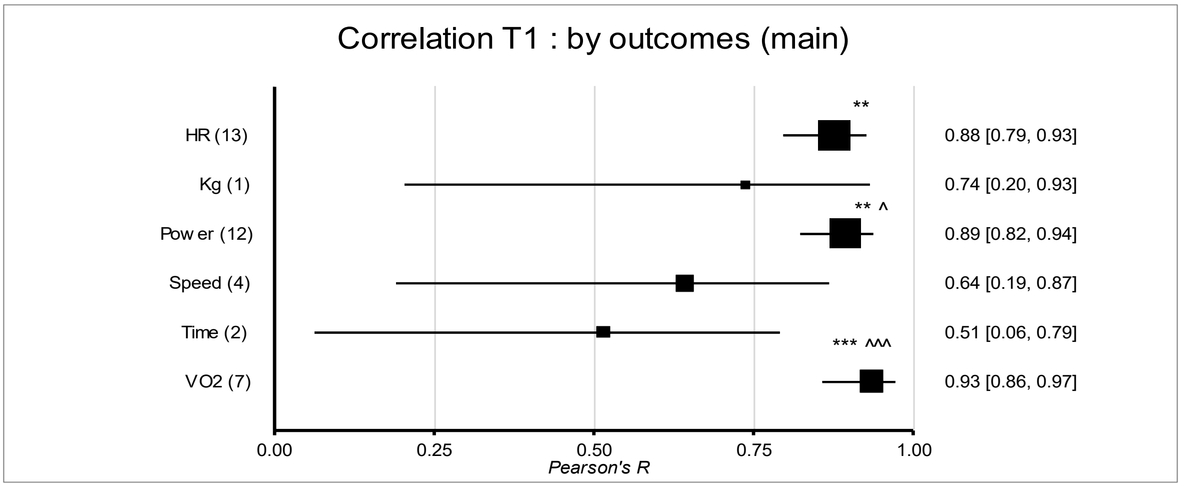

***p < 0.01 and ***p < 0.001 for difference with Time; ^p < 0.05 and ^^p < 0.01 for difference with Speed*

Between groups: **p = 0.004**

Time Pearson's R is lower than:

- HR: p = 0.007
- Power: p = 0.003
- VO2: p < 0.001

Speed Pearson's R is lower than:

- Power: p = 0.035
- VO2: p < 0.001

	Heterogeneity	
	P-value	*I-squared (%)*
HR	0.000	90.7
Kg	1	0.0
Power	0.000	83.8
Speed	0.000	81.8
Time	0.000	94.5
VO2	0.000	90.3

There is no other difference in Pearson's R between subgroups.

Outcomes formats:

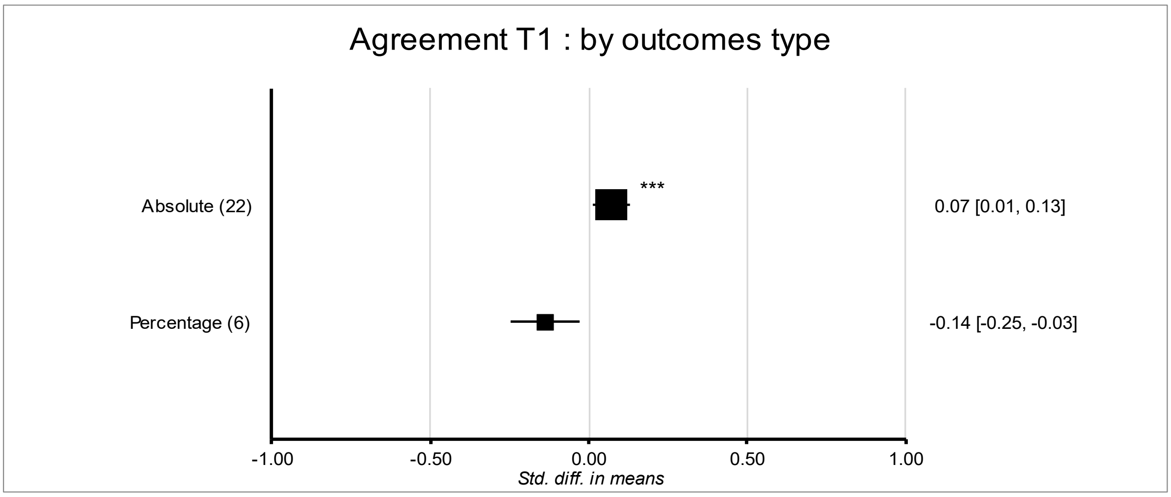

***p < 0.01 for difference with Percentage*

Between subgroups: **p < 0.001**

The std. diff. in means are different between subgroups.

	Heterogeneity	
	P-value	***I-squared (%)***
T1-HR	0.000	89.5
T1-Kg	0.000	84.9

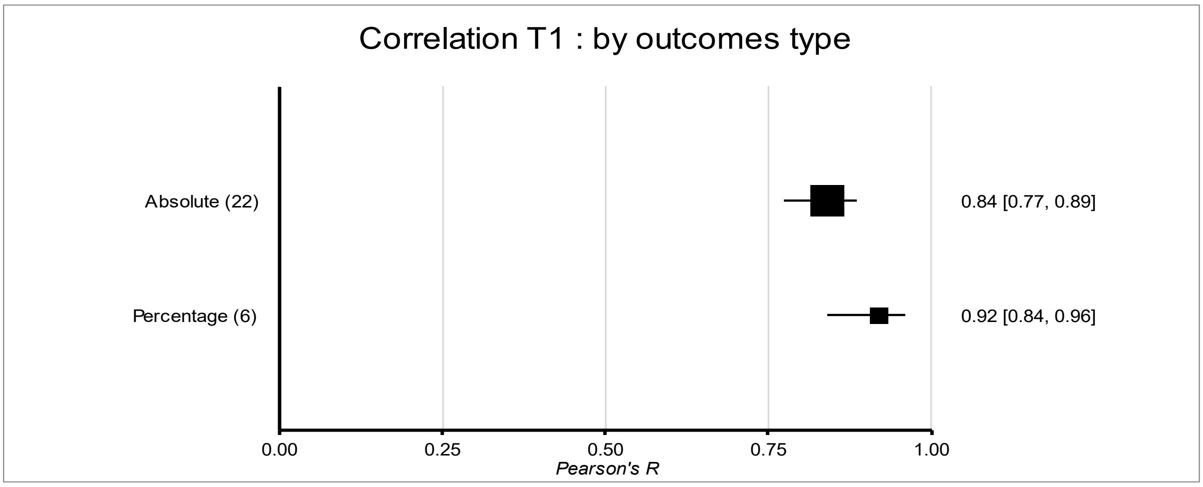

Between subgroups: p = 0.078

There is no difference in Pearson's R between subgroups.

	Heterogeneity	
	P-value	***I-squared (%)***
Percentage	0.000	89.5
Absolute value	0.000	84.9

Ergometers:

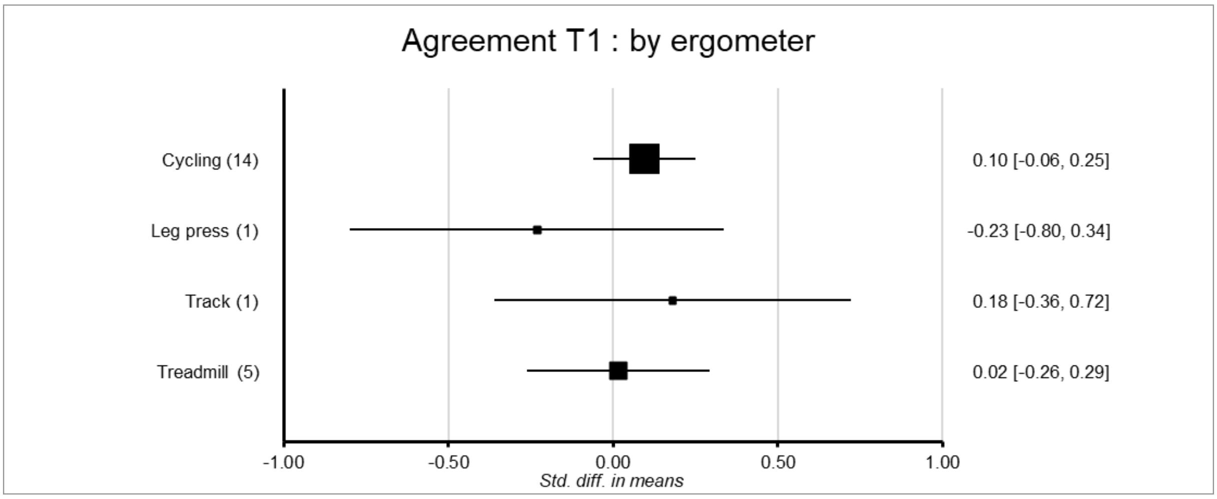

Between subgroups: p = 0.684

There is no difference in std. diff. in means between subgroups.

	Heterogeneity	
	P-value	*I-squared (%)*
Cycling	0.000	91.4
Leg press	1	0.0
Track	1	0.0
Treadmill	0.000	82.3

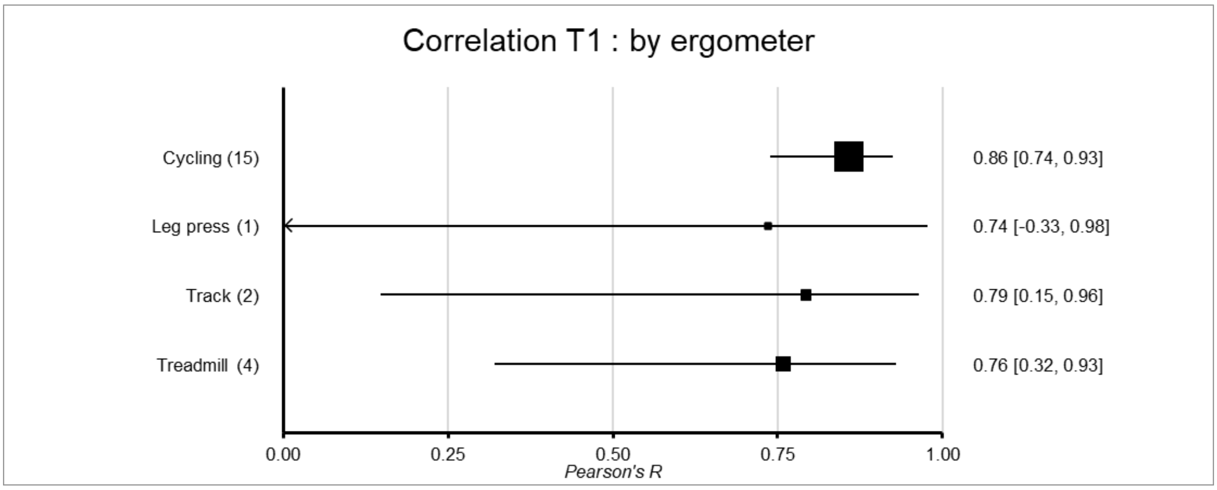

Between subgroups: p = 0.841

There is no difference in Pearson's R between subgroups.

	Heterogeneity	
	P-value	*I-squared (%)*
Cycling	0.000	91.7
Leg press	1	0.0
Track	0.000	96.5
Treadmill	0.000	91.4

Initial workload:

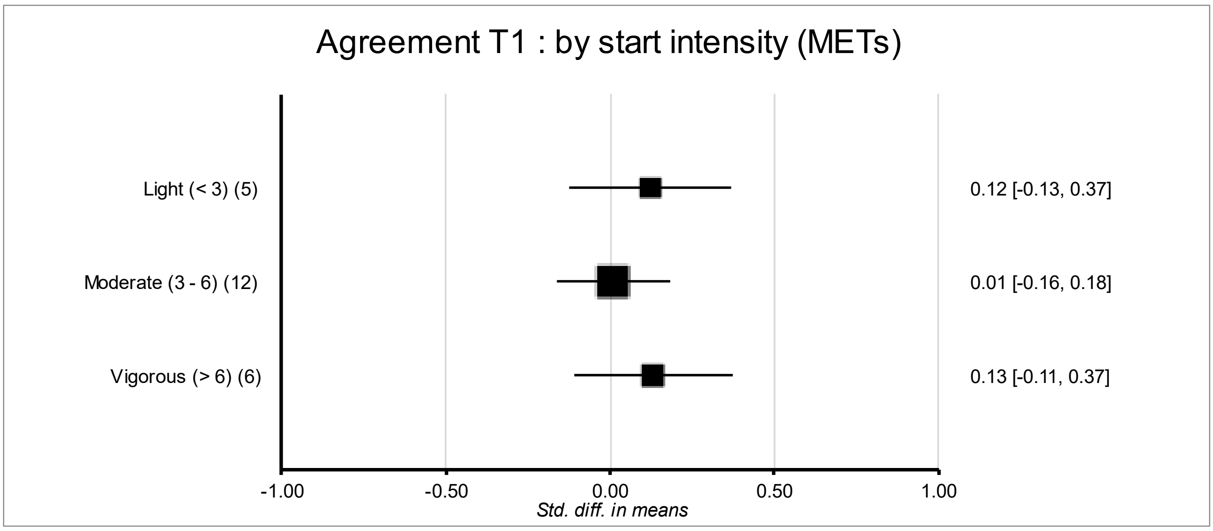

Between subgroups: p = 0.640

There is no difference in std. diff. in means between subgroups.

	Heterogeneity	
	P-value	*I-squared (%)*
Light (< 3)	0.000	86.3
Moderate (3 - 6)	0.000	91.8
Vigorous (> 6)	0.000	79.2

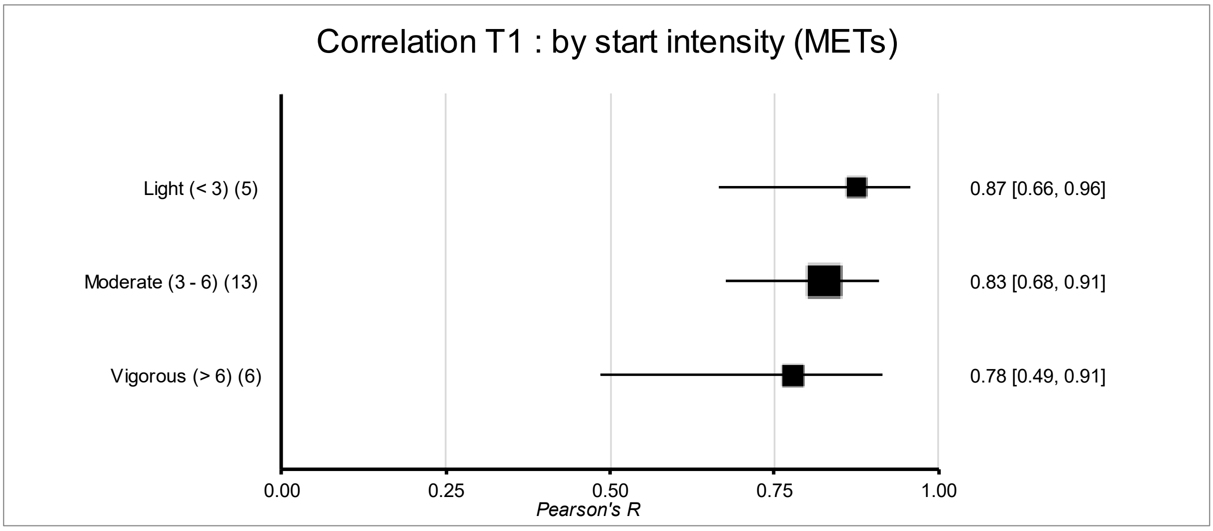

Between subgroups: p = 0.717

There is no difference in Pearson's R between subgroups.

	Heterogeneity	
	P-value	*I-squared (%)*
Light (< 3)	0.000	90.8
Moderate (3 - 6)	0.000	89.4
Vigorous (> 6)	0.000	95.1

Increment workload (METs) :

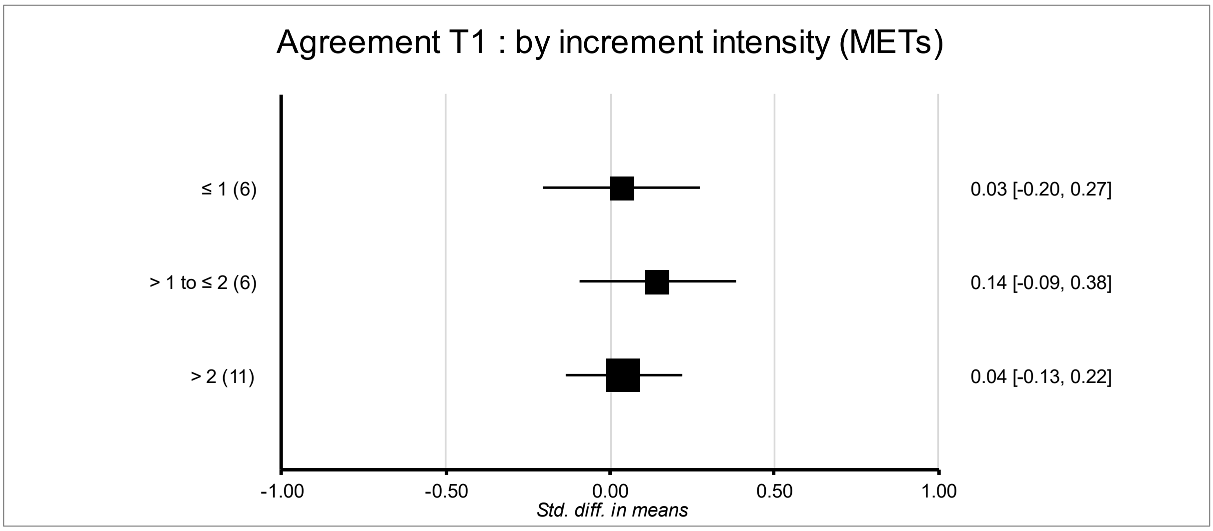

Between subgroups: p = 0.755

There is no difference in std. diff. in means between subgroups.

	Heterogeneity	
	P-value	*I-squared (%)*
≤ 1	0.000	80.7
> 1 to ≤ 2	0.001	76.4
> 2	0.000	93.0

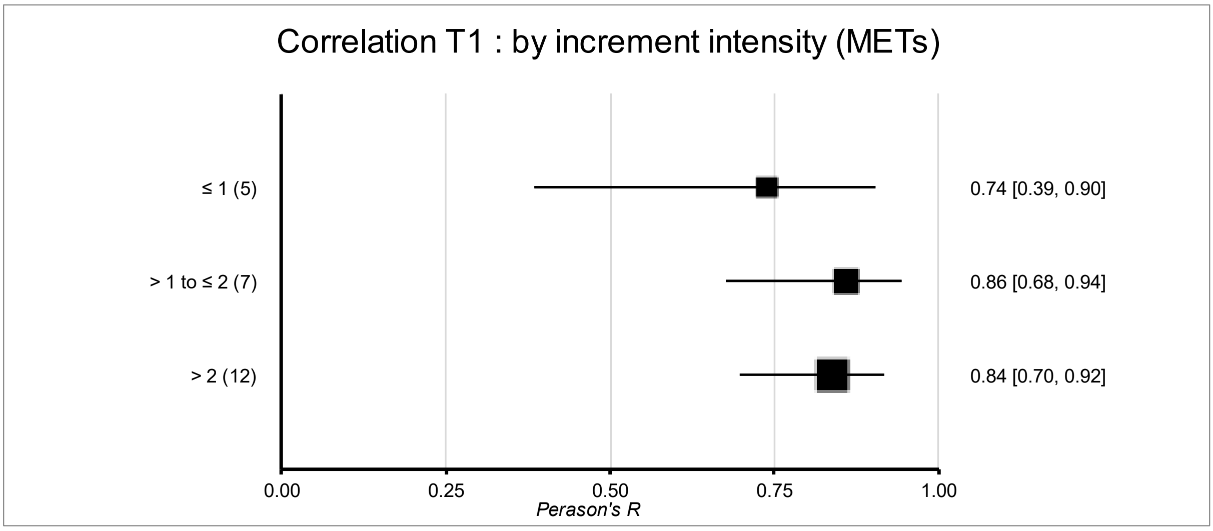

Between subgroups: p = 0.617

There is no difference in Pearson's R between subgroups.

	Heterogeneity	
	P-value	*I-squared (%)*
≤ 1	0.000	92.7
> 1 to ≤ 2	0.000	88.5
> 2	0.000	92.1

Increment workload (%) :

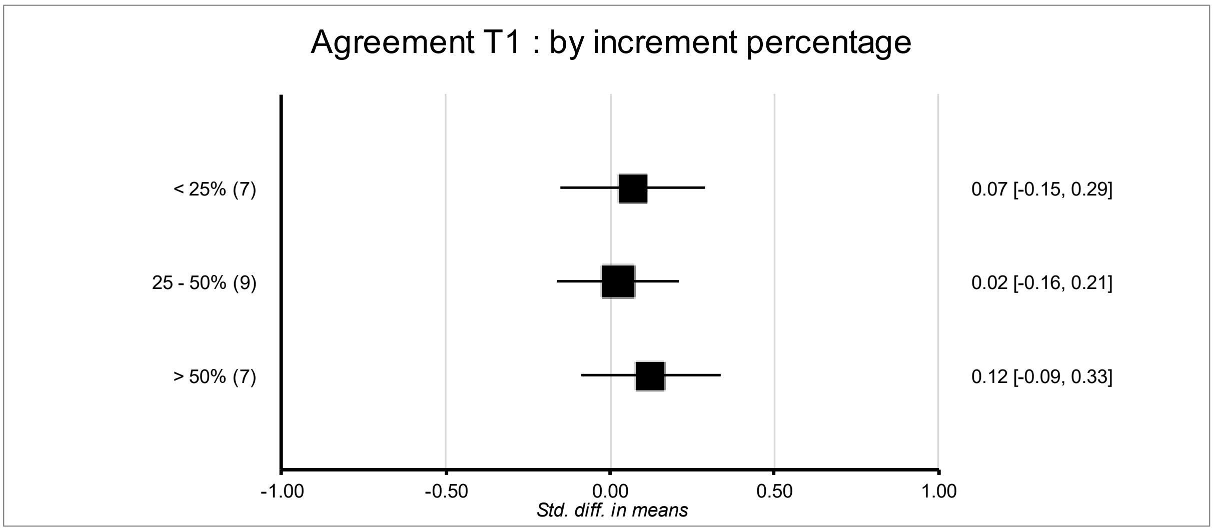

Between subgroups: p = 0.786

There is no difference in std. diff. in means between subgroups.

	Heterogeneity	
	P-value	*I-squared (%)*
< 25%	0.000	78.9
25 - 50%	0.002	67.3
> 50%	0.000	95.3

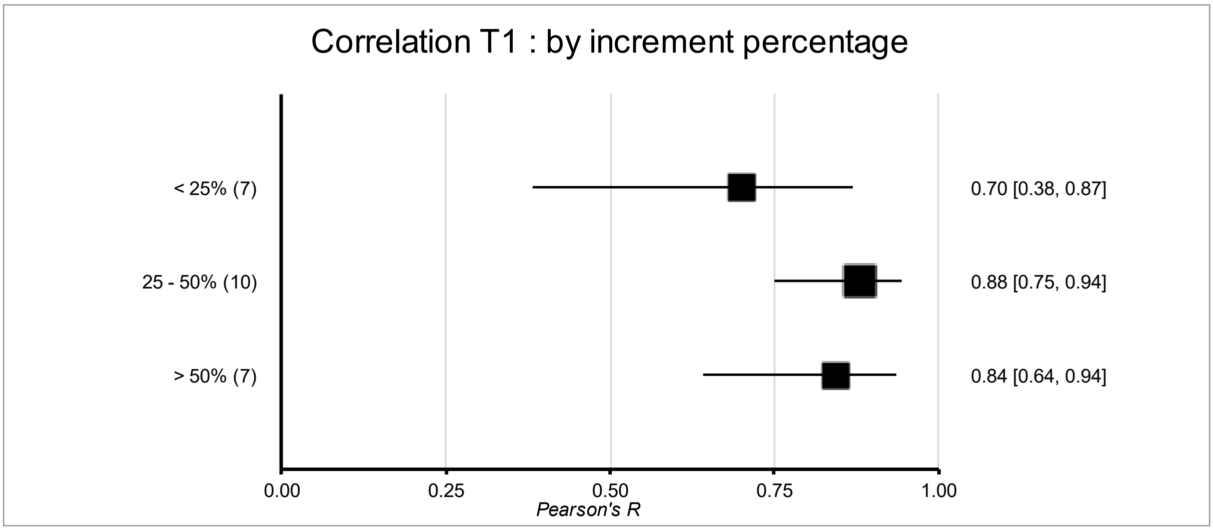

Between subgroups: p = 0.259

There is no difference in Pearson's R between subgroups.

	Heterogeneity	
	P-value	*I-squared (%)*
< 25%	0.000	91.4
25 - 50%	0.000	94.6
> 50%	0.000	75.5

Increment duration:

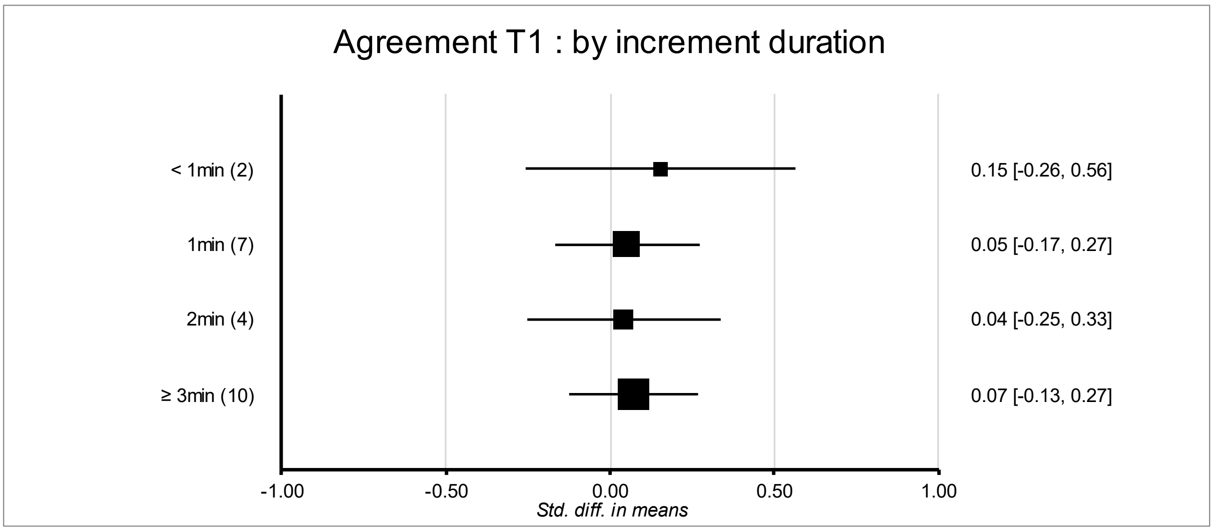

Between subgroups: p = 0.974

There is no difference in std. diff. in means between subgroups.

	Heterogeneity	
	P-value	*I-squared (%)*
< 1min	0.051	73.8
1min	0.000	77.0
2min	0.000	87.3
≥ 3min	0.000	93.3

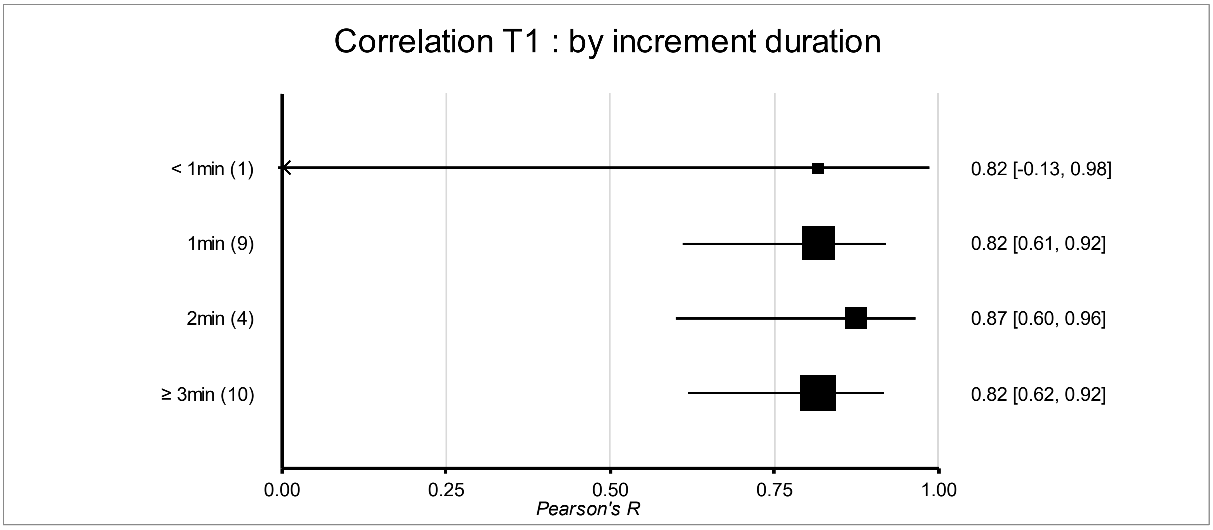

Between subgroups: p = 0.959

There is no difference in Pearson's R between subgroups.

	Heterogeneity	
	P-value	*I-squared (%)*
< 1min	1	0.0
1min	0.000	94.4
2min	0.000	86.4
≥ 3min	0.000	86.4

Continent:

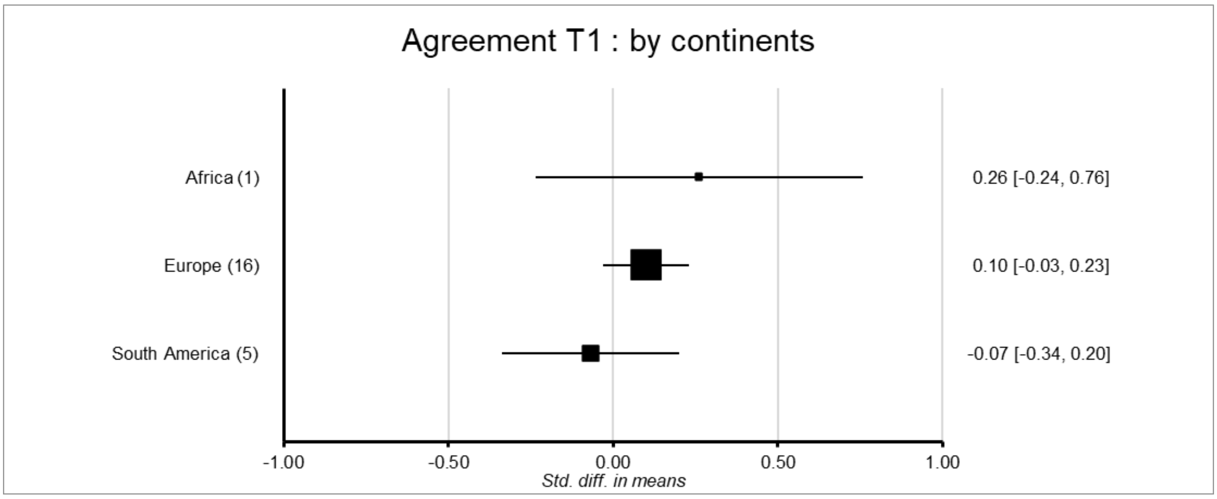

Between subgroups: p = 0.413

There is no difference in std. diff. in means between subgroups.

	Heterogeneity	
	P-value	***I-squared (%)***
Africa	1	0.0
Europe	0.000	90.2
South America	0.000	84.5

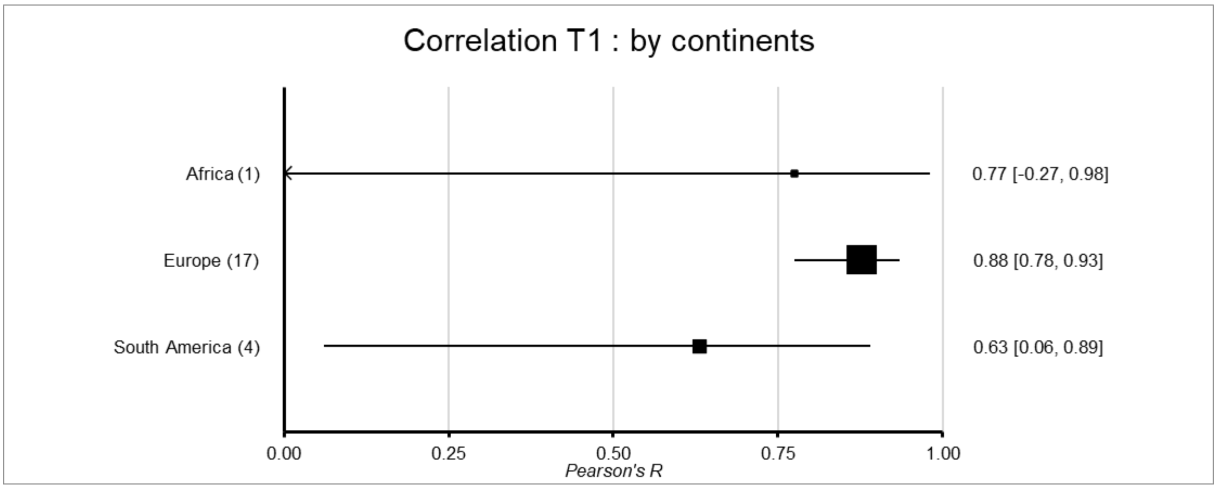

Between subgroups: p = 0.264

There is no difference in Pearson's R between subgroups.

	Heterogeneity	
	P-value	***I-squared (%)***
Africa	1	0.0
Europe	0.000	94.6
South America	0.118	49.0
