# Supplementary material for: Agreement Between Heart Rate Variability - Derived vs. Ventilatory and Lactate Thresholds: A Systematic Review with Meta-Analyses
Source: Sports Med Open. 2024 Oct 8;10:109. doi: 10.1186/s40798-024-00768-8 (PMC11461412; doi:10.1186/s40798-024-00768-8)
Supplement: Supplementary file 6 — Supplementary Material 6: HRVT2 moderator analyses [file 40798_2024_768_MOESM6_ESM.pdf]

Agreement between heart rate variability - derived vs. ventilatory and lactate thresholds: A systematic review with meta-analyses

Electronic Supplementary Material 6

HRVT2 Moderator analyses

Sports Medicine - Open

Valérian Tanner[1]*, Grégoire P. Millet[1], Nicolas Bourdillon[1]

[1]Institute of Sport Sciences, University of Lausanne, Lausanne, Switzerland.

*Corresponding author:
- E-mail: valerian.tanner@unil.ch
- Adress: Quartier UNIL-Centre, Bâtiment Synathlon, 1015 Lausanne, Switzerland.

Subjects characteristics 3
Age: 3
Gender: 4
Weight class: 5
Training status: 6
Health status: 7
Pathology: 8
HRVT2 and LT2-VT2 determination methods 9
Reference thresholds: 9
Reference threshold determination type: 10
HRV domains: 11
HRV variables: 12
Number of HRV variables used for HRVT determination: 14
HRVT2 determination type: 15
HRVT2 determination complexity: 16
HRV recording device: 17
HRV recording device type: 19
HRV Software: 20
Study protocol 21
Outcomes: 21
Outcomes format: 23
Ergometers: 24
Initial workload: 25
Increment workload (METs): 26
Increment workload (%): 27
Increment duration: 28
Continent: 29

Subjects characteristics

Age:

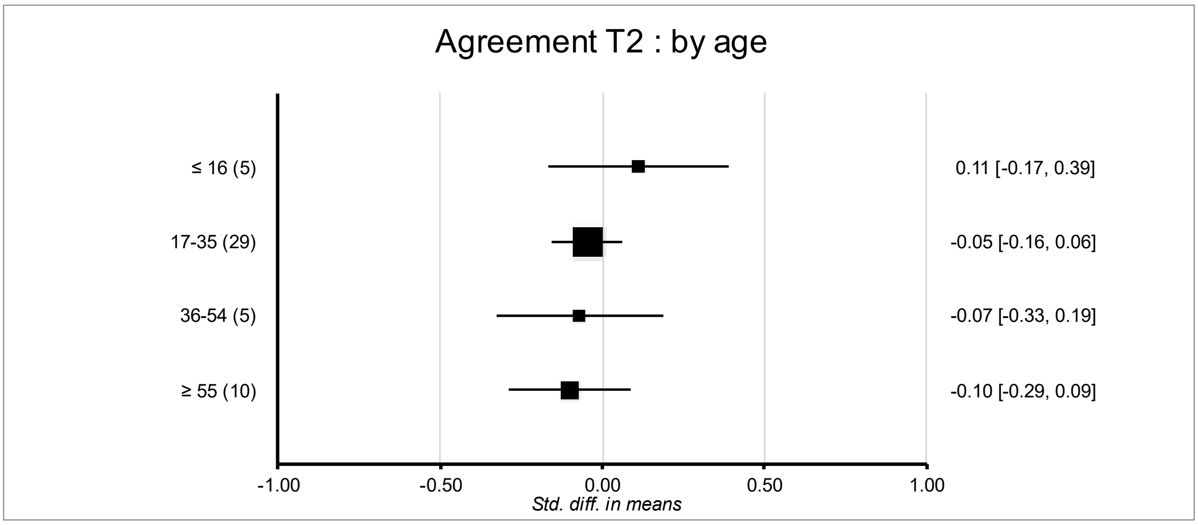

Between subgroups: p = 0.661

There is no difference in std. diff. in means between subgroups.

	Heterogeneity	
	P-value	*I-squared (%)*
≤ 16	0.665	0.0
18-35	0.000	94.5
36-54	0.040	60.0
≥ 55	0.729	0.0

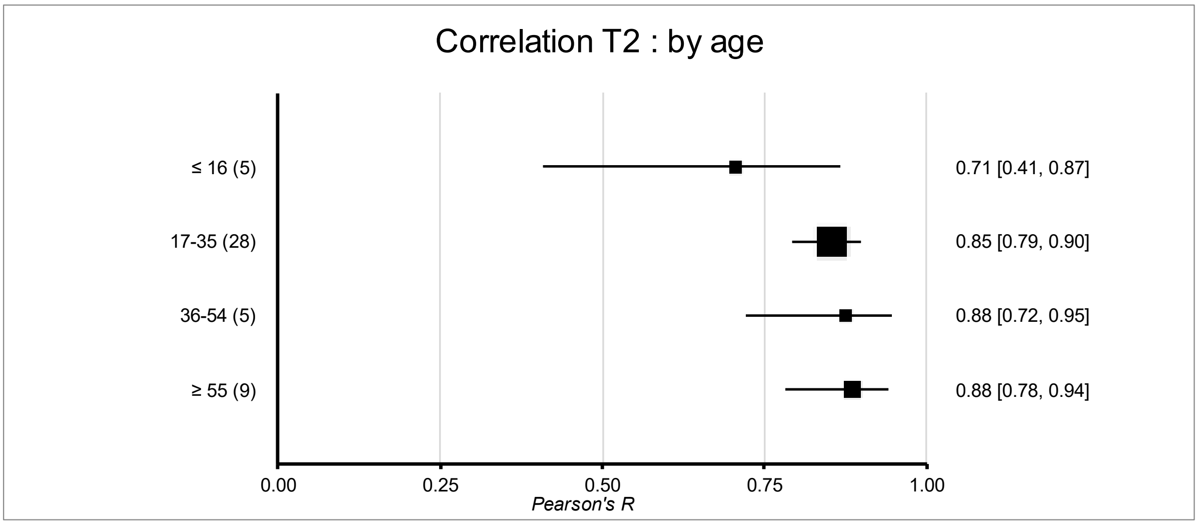

Between subgroups: p = 0.302

There is no difference in Pearson's R between subgroups.

	Heterogeneity	
	P-value	*I-squared (%)*
≤ 16	0.000	86.3
17-35	0.000	89.7
36-54	0.000	91.9
≥ 55	0.000	77.7

Gender:

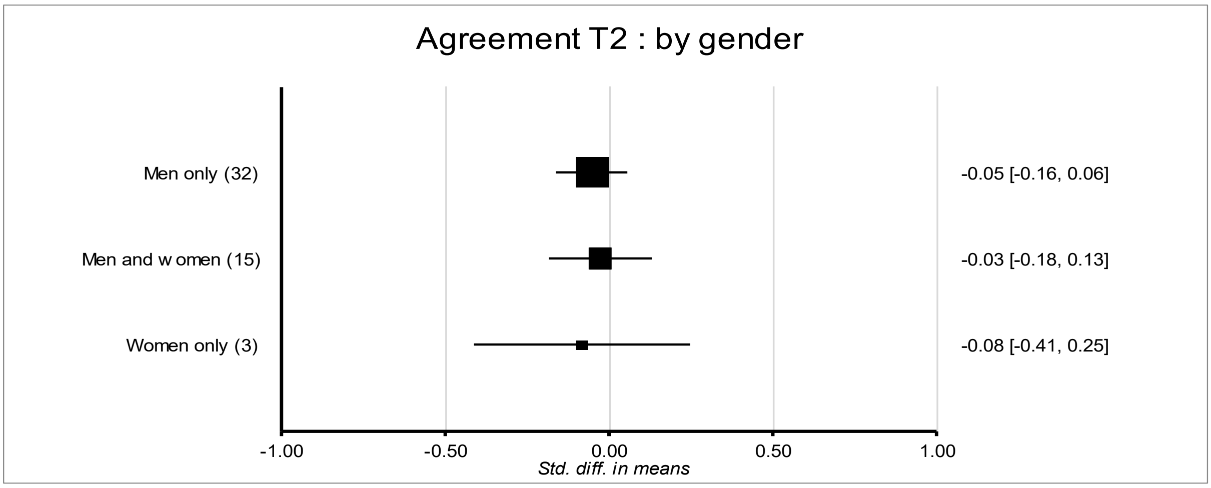

Between subgroups: p = 0.940

There is no difference in std. diff. in means between subgroups.

	Heterogeneity	
	P-value	*I-squared (%)*
Men only	0.000	94.3
Men and women	0.006	54.5
Women only	0.462	0.0

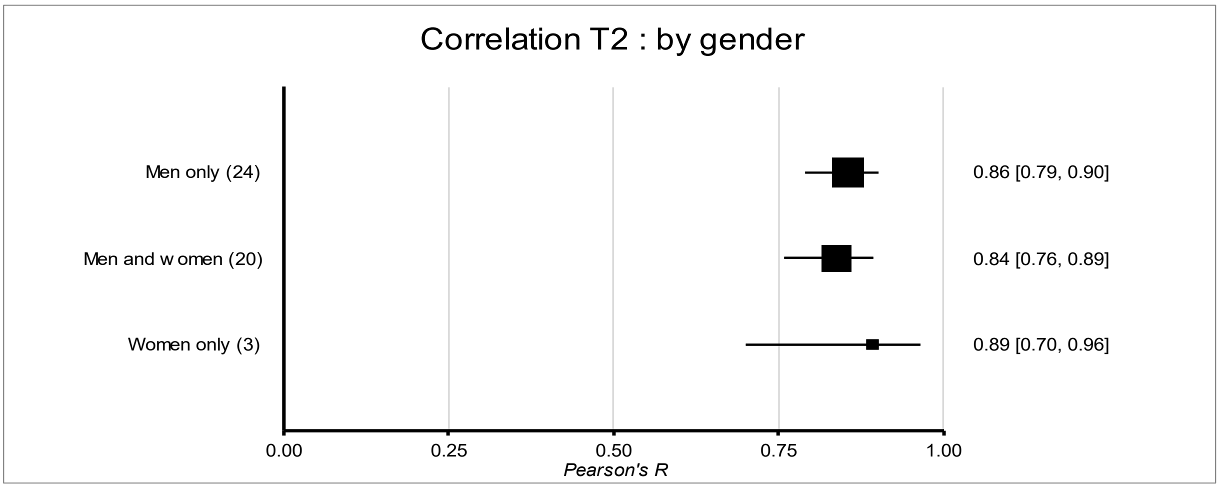

Between subgroups: p = 0.763

There is no difference in Pearson's R between subgroups.

	Heterogeneity	
	P-value	*I-squared (%)*
Men only	0.000	85.6
Men and women	0.000	93.1
Women only	0.795	0.0

Weight class:

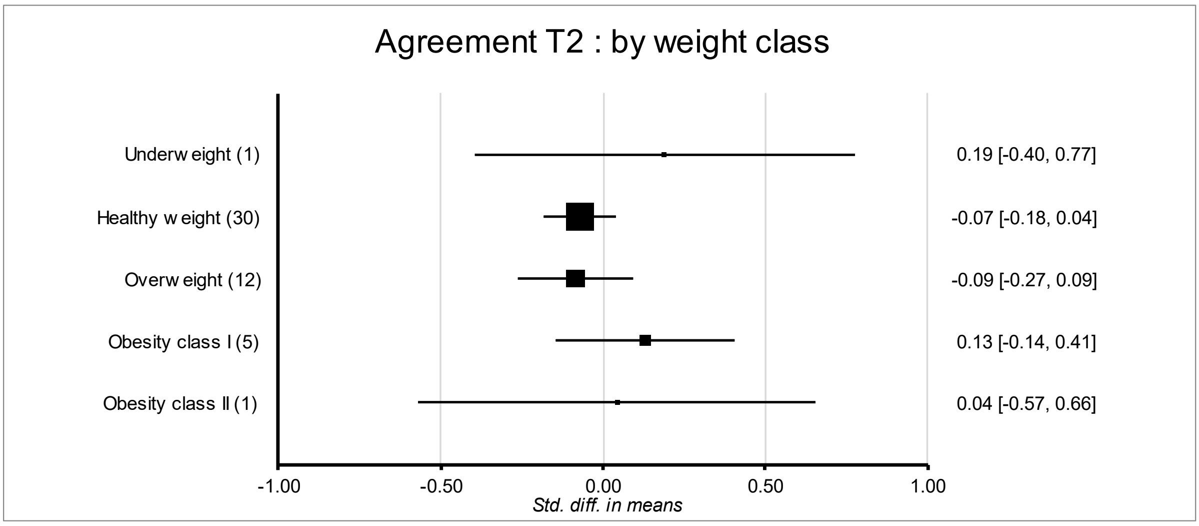

Underweight, BMI < 18.5; Healthy weight, BMI 18.5 – 25; Overweight, BMI 25 – 30; Obesity class I, BMI 30 – 35; Obesity class II, BMI 35 – 40.

Between subgroups: p = 0.609

There is no difference in std. diff. in means between subgroups.

	Heterogeneity	
	P-value	***I-squared (%)***
Underweight	1	0.0
Healthy weight	0.000	93.9
Overweight	0.000	78.1
Obesity class I	0.000	90.9
Obesity class II	1	0.0

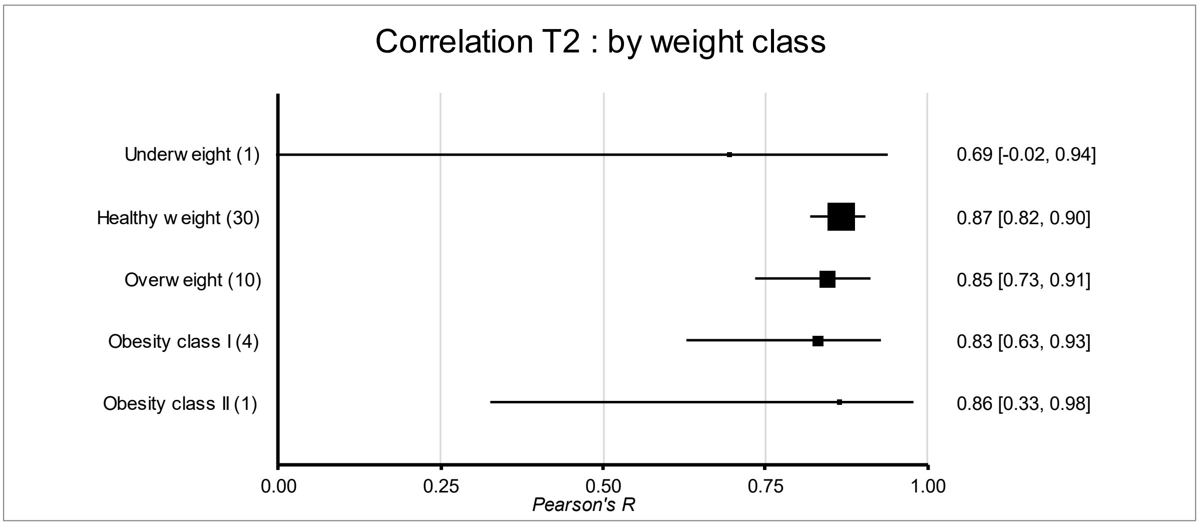

Healthy weight, BMI 18.5 – 25; Overweight, BMI 25 – 30; Obesity class I, BMI 30 – 35.

Between subgroups: p = 0.849

There is no difference in Pearson's R between subgroups.

	Heterogeneity	
	P-value	***I-squared (%)***
Underweight	1	0.0
Healthy weight	0.000	88.0
Overweight	0.000	88.0
Obesity class I	0.007	75.3
Obesity class II	1	0.0

Training status:

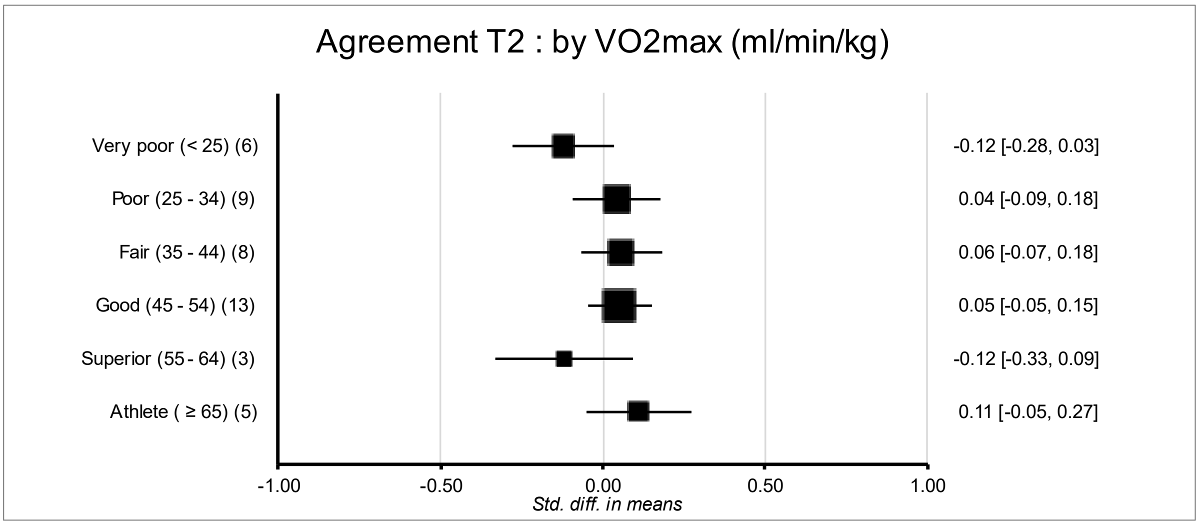

Between subgroups: p = 0.223

There is no difference in std. diff. in means between subgroups.

	Heterogeneity	
	P-value	*I-squared (%)*
Very poor (< 25)	0.481	0.0
Poor (25 - 34)	0.000	81.0
Fair (35 - 44)	0.002	69.3
Good (45 - 54)	0.002	62.0
Superior (55 - 64)	0.656	0.0
Athlete (≥ 65)	0.000	93.7

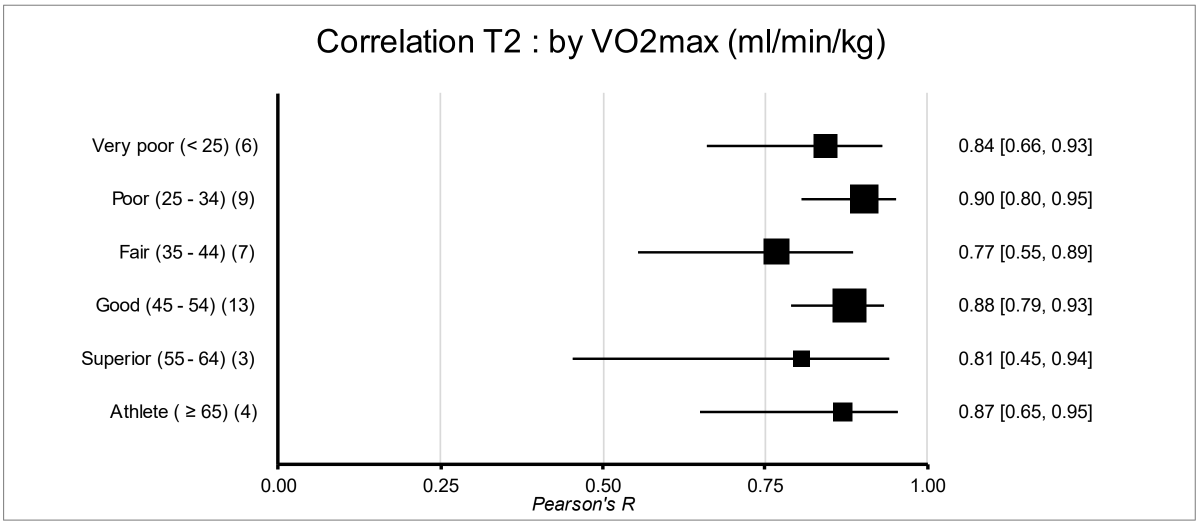

Between subgroups: p = 0.597

There is no difference in Pearson's R between subgroups.

	Heterogeneity	
	P-value	*I-squared (%)*
Very poor (< 25)	0.000	86.3
Poor (25 - 34)	0.223	24.8
Fair (35 - 44)	0.000	92.5
Good (45 - 54)	0.000	95.2
Superior (55 - 64)	0.198	38.2
Athlete (≥ 65)	0.000	88.8

Health status:

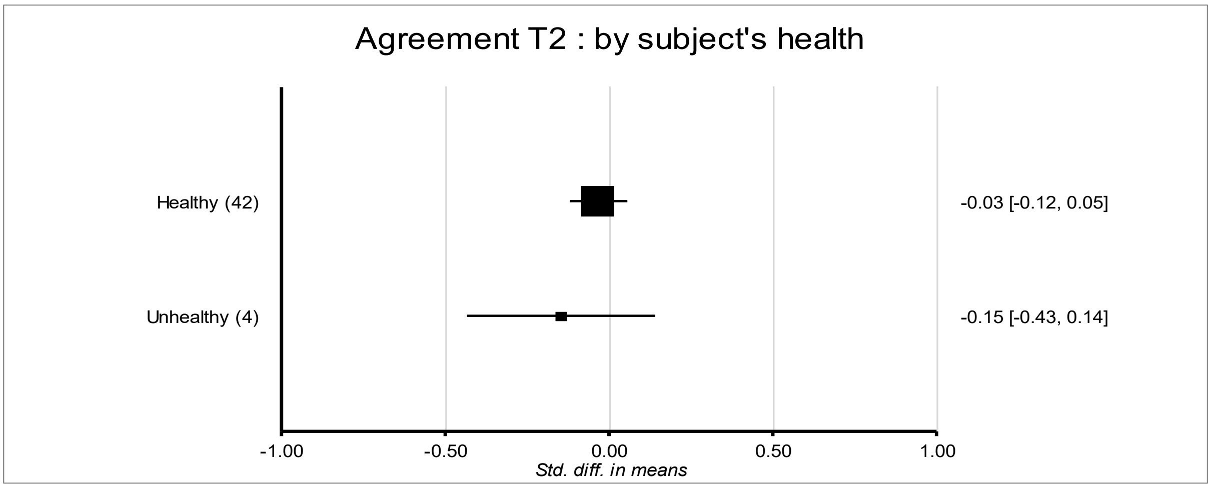

Between subgroups: p = 0.466

There is no difference in std. diff. in means between subgroups.

	Heterogeneity	
	P-value	*I-squared (%)*
Healthy	0.000	92.3
Non Healthy	0.140	45.2

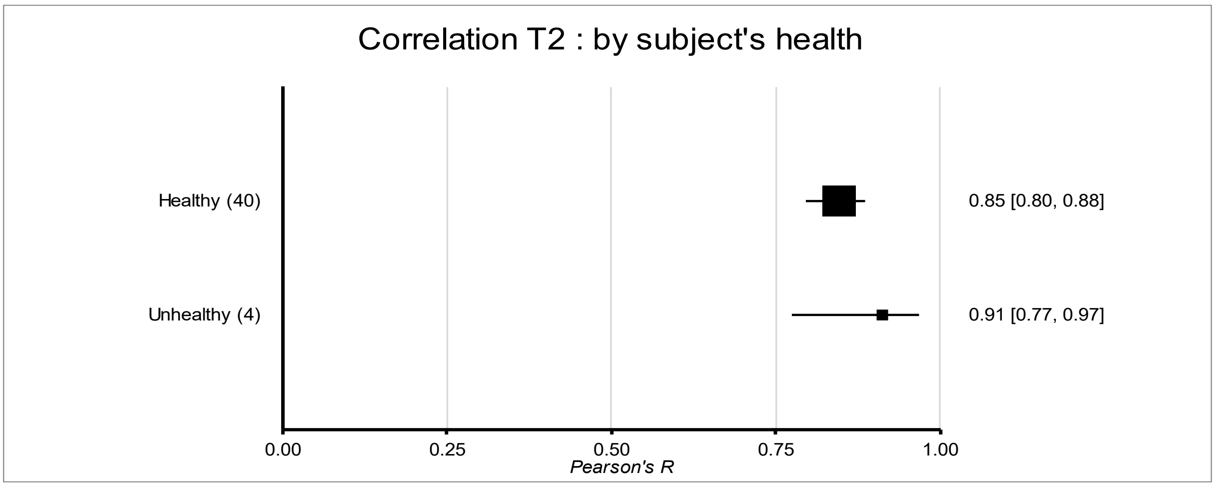

Between subgroups: p = 0.269

There is no difference in Pearson's R between subgroups.

	Heterogeneity	
	P-value	*I-squared (%)*
Healthy	0.000	91.2
Unhealthy	0.058	59.9

Pathology:

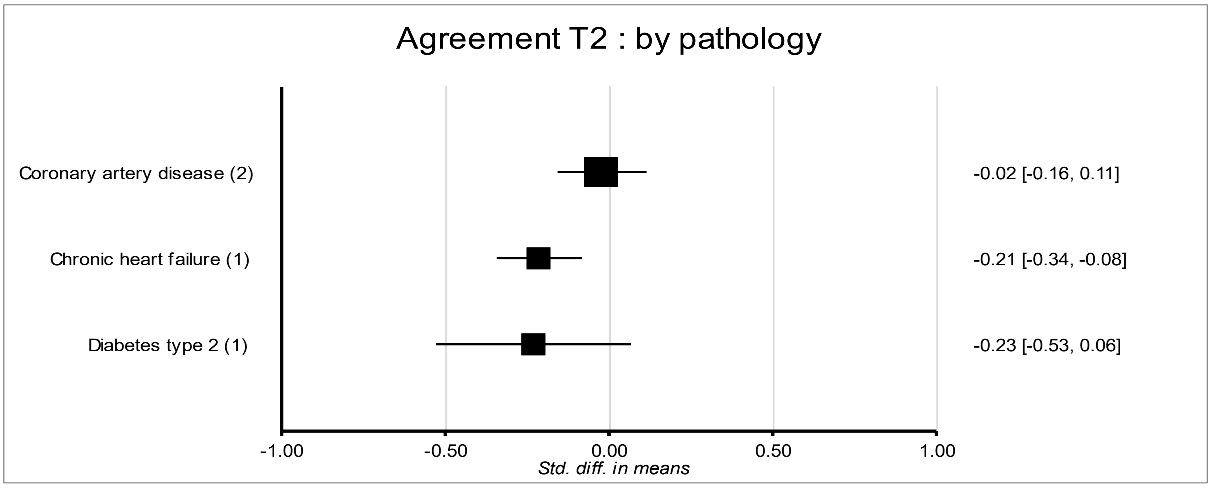

Between subgroups: p = 0.106

There is no difference in std. diff. in means between subgroups.

	Heterogeneity	
	P-value	*I-squared (%)*
Coronary artery disease	0.322	0.0
Chronic heart failure	1	0.0
Diabetes type 2	1	0.0

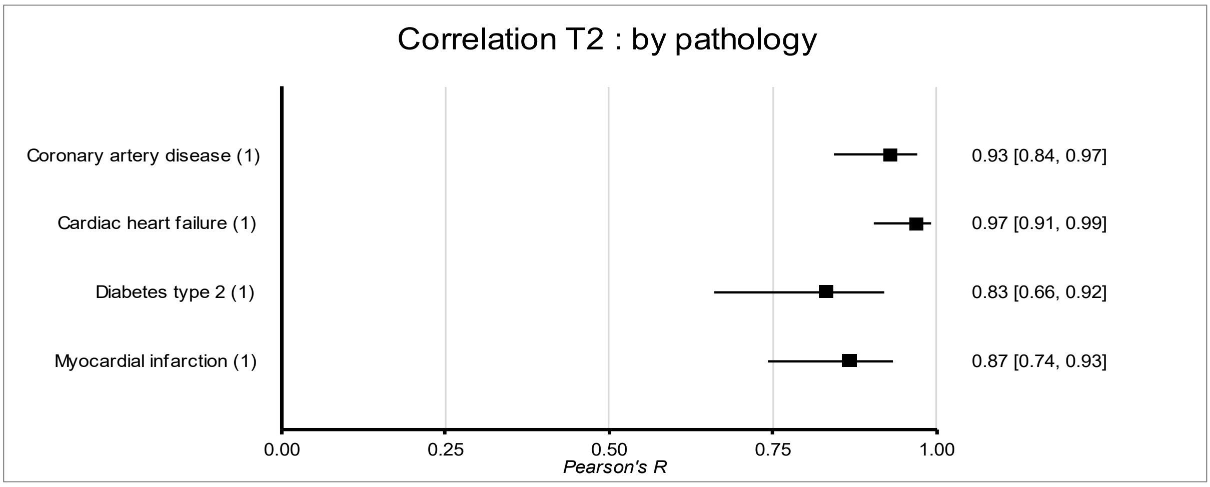

Between subgroups: p = 0.058

There is no difference in Pearson's R between subgroups.

	Heterogeneity	
	P-value	*I-squared (%)*
Coronary artery disease	1	0.0
Cardiac heart failure	1	0.0
Diabetes type 2	1	0.0
Myocardial infarction	1	0.0

HRVT2 and LT2-VT2 determination methods

Reference thresholds:

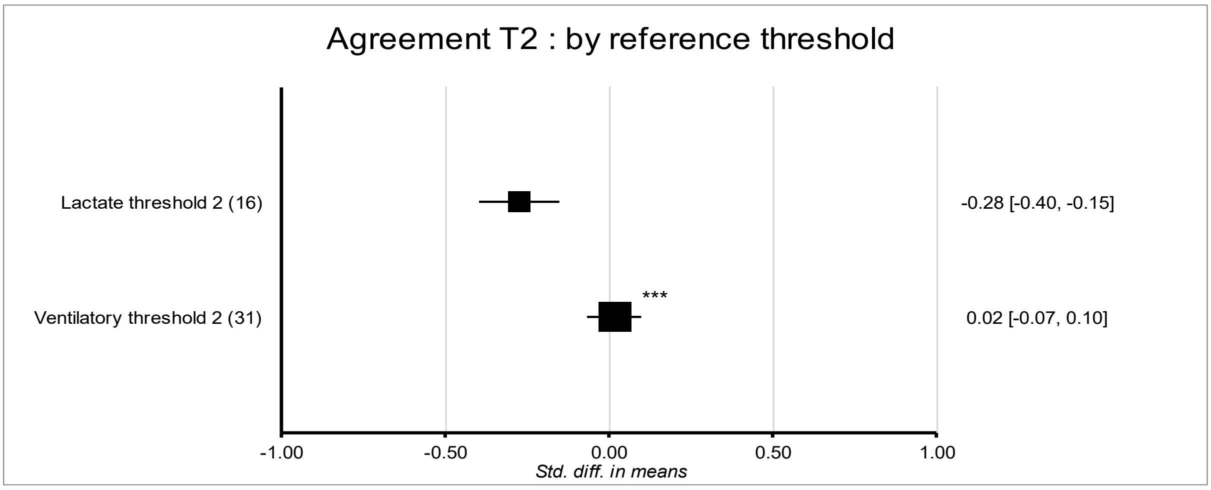

****p < 0.001 for difference with LT2*

Between subgroups: **p < 0.001**

The std. diff. in means are different between subgroups.

	Heterogeneity	
	P-value	*I-squared (%)*
Lactate threshold 2	0.000	91.4
Ventilatory threshold 2	0.000	79.5

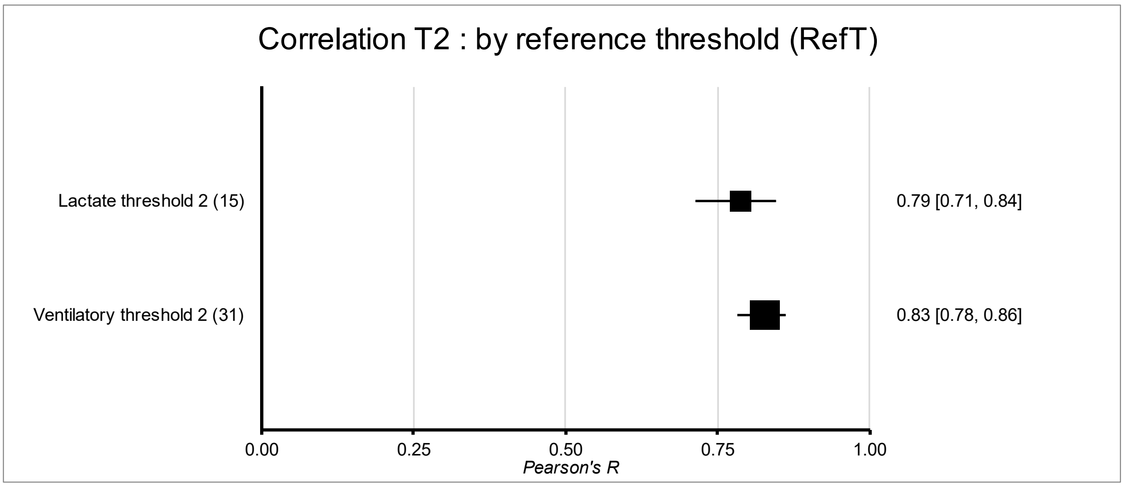

Between subgroups: p = 0.298

There is no difference in Pearson's R between subgroups.

	Heterogeneity	
	P-value	*I-squared (%)*
Lactate threshold 2	0.000	63.5
Ventilatory threshold 2	0.000	84.8

Reference threshold determination type:

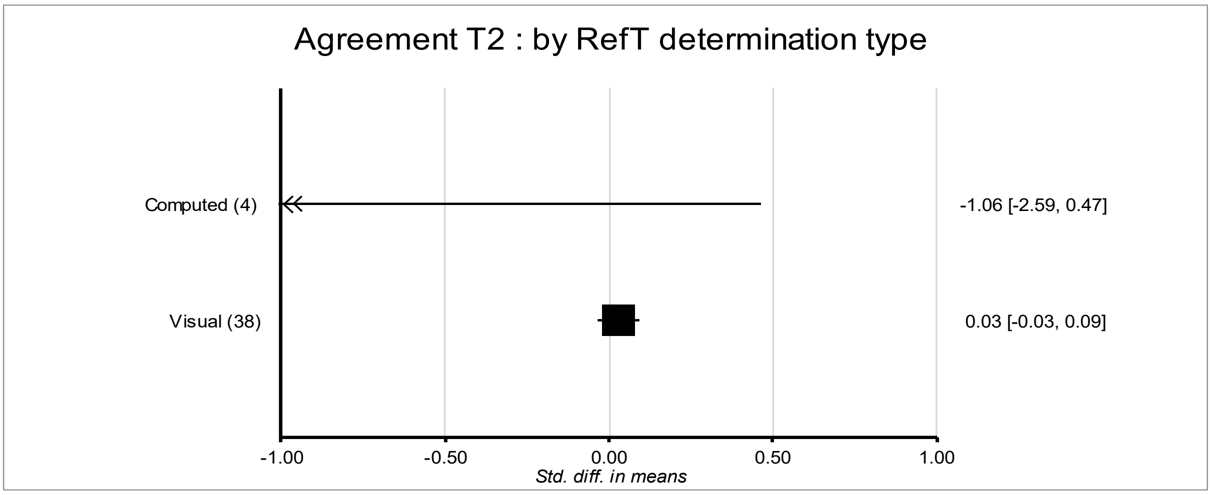

Between subgroups: p = 0.162

There is no difference in std. diff. in means between subgroups.

	Heterogeneity	
	P-value	*I-squared (%)*
Computed	0.000	95.3
Visual	0.000	79.2

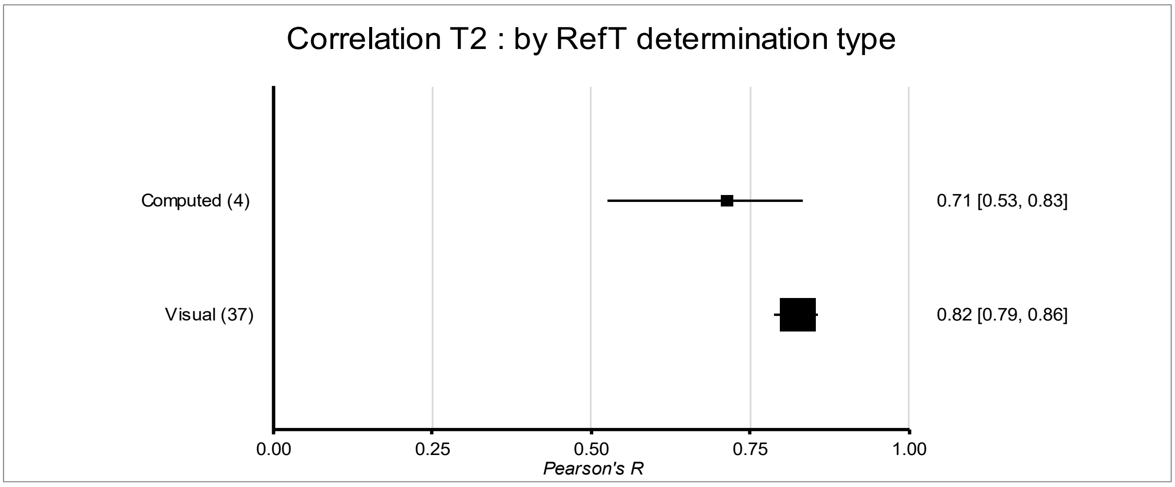

Between subgroups: p = 0.326

There is no difference in Pearson's R between subgroups.

	Heterogeneity	
	P-value	*I-squared (%)*
Computed	0.731	0.0
Visual	0.000	82.4

HRV domains:

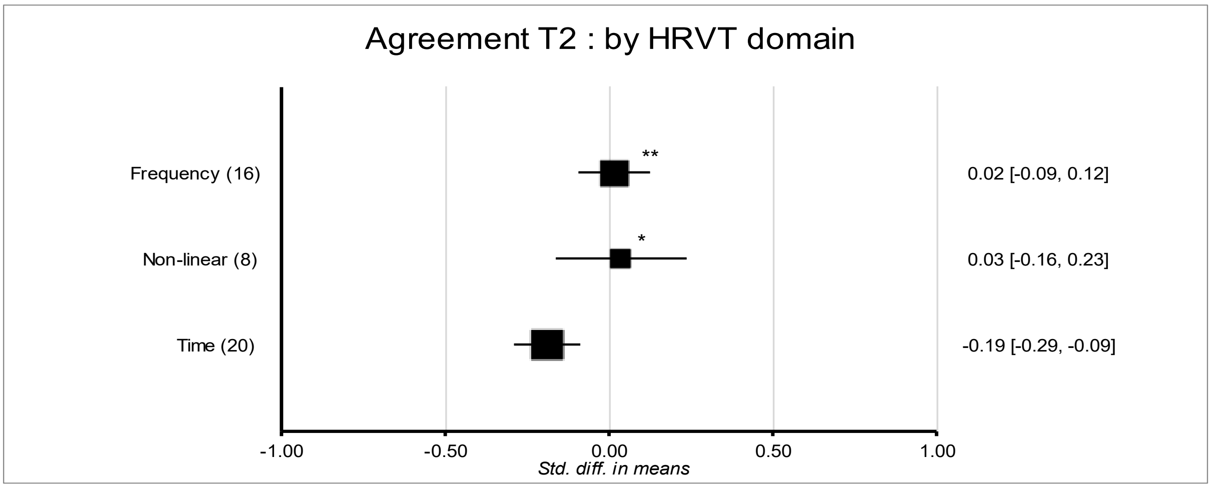

**p < 0.05 and **p < 0.01 for difference with Time*

Between groups: **p = 0.012**

Time std. diff. in means is lower than:

- Frequency: p = 0.006
- Non-linear: p = 0.047

There is no other difference in std. diff. in means between subgroups.

	Heterogeneity	
	P-value	***I-squared (%)***
Frequency	0.000	81.3
Non-linear	0.000	69.4
Time	0.000	88.9

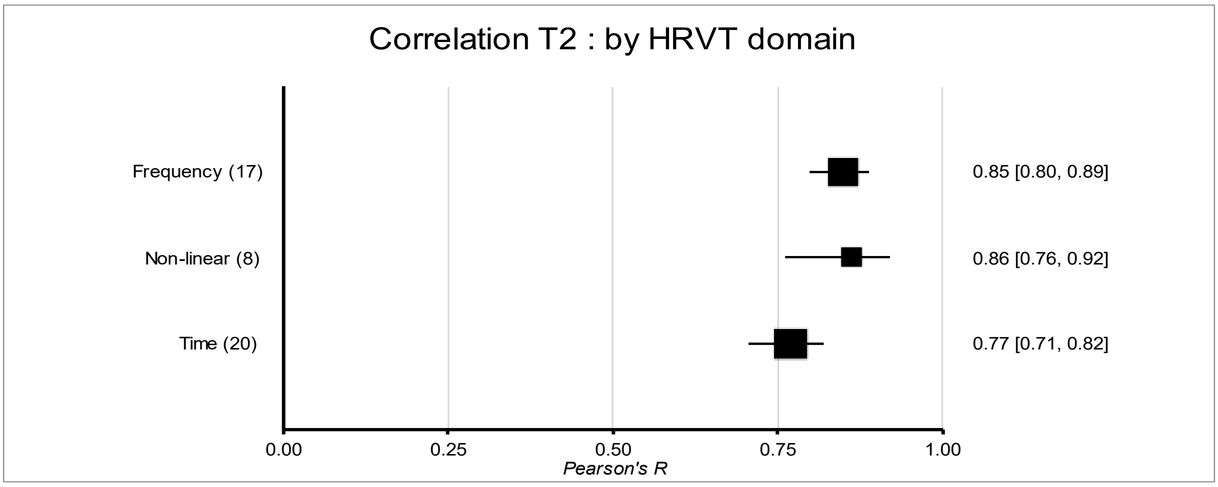

HRVT, heart rate variability threshold

Between subgroups: p = 0.055

There is no other difference in Pearson's R between subgroups.

	Heterogeneity	
	P-value	***I-squared (%)***
Frequency	0.000	84.3
Non-linear	0.000	67.6
Time	0.000	76.3

HRV variables:

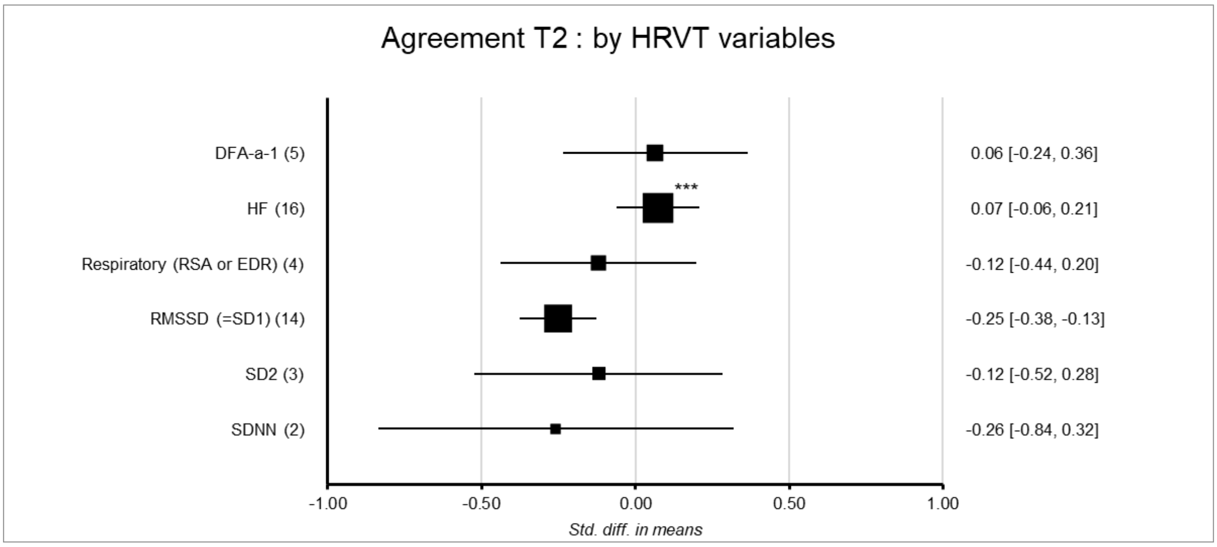

****p* < 0.001 for difference with RMSSD (=SD1)*

Between groups: **p = 0.022**

RMSSD (=SD1) std. diff. in means is lower than:

- High frequency: $p < 0.001$

There is no other difference in std. diff. in means between subgroups.

	Heterogeneity	
	P-value	***I-squared (%)***
DFA-a-1	0.000	79.3
HF	0.000	81.2
Respiratory (RSA or EDR)	0.142	41.9
RMSSD (=SD1)	0.000	90.8
SD2	0.000	88.0
SDNN	0.079	67.5

Correlation T2: by HRV variables on the next page

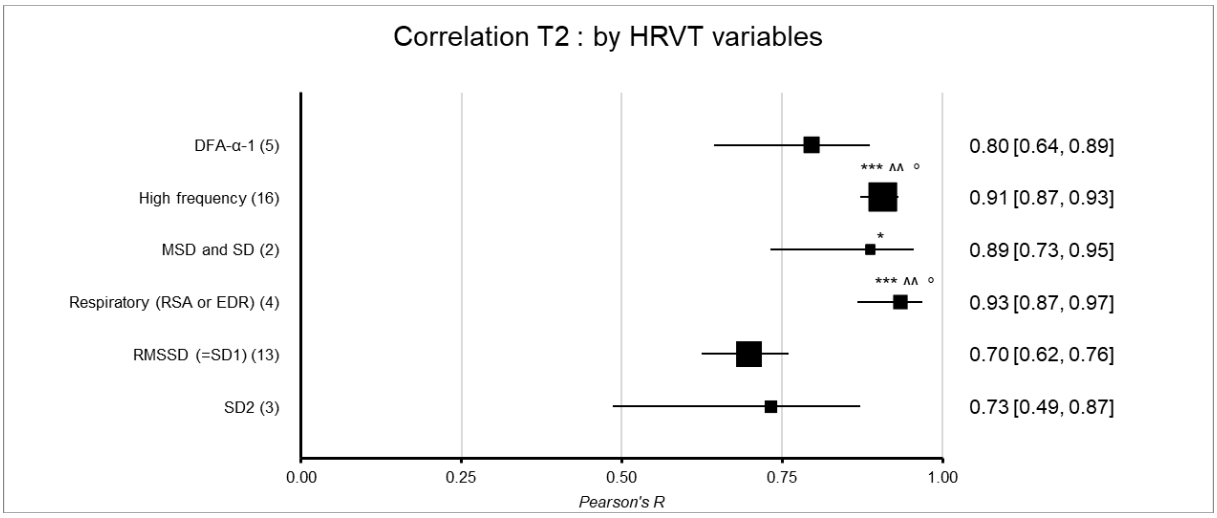

**p < 0.05 and ***p < 0.001 for difference with RMSSD (=SD1); ^^p < 0.01 for difference with SD2; °p < 0.05 for difference with DFA-α-1.*

Between groups: **p < 0.001**

Differences among subgroups with more than one study are the following:

RMSSD (=SD1) Pearson's R is lower than:

- High frequency: p < 0.001
- Respiratory (RSA or EDR): p < 0.001
- MSD and SD: p = 0.031

SD2 Pearson's R is lower than:

- High frequency: p = 0.009
- Respiratory (RSA or EDR): p = 0.006

DFA-α-1 Pearson's R is lower than:

- High frequency: p = 0.023
- Respiratory (RSA or EDR): p = 0.016

	**Heterogeneity**	
	P-value	***I-squared (%)***
DFA-α-1	0.002	72.8
High frequency	0.000	72.9
MSD and SD	0.324	11.3
Respiratory (RSA or EDR)	0.001	78.3
RMSSD (=SD1)	0.000	60.4
SD2	0.056	60.4

Number of HRV variables used for HRVT determination:

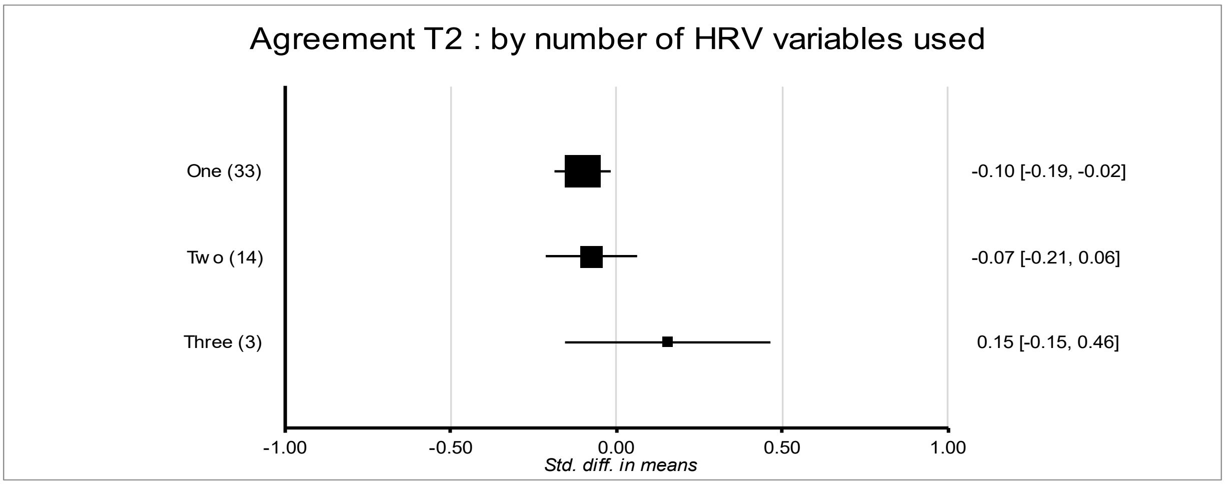

Between subgroups: p = 0.294

There is no difference in std. diff. in means between subgroups.

Heterogeneity

	P-value	*I-squared (%)*
One	0.000	88.2
Two	0.000	68.0
Three	0.000	88.1

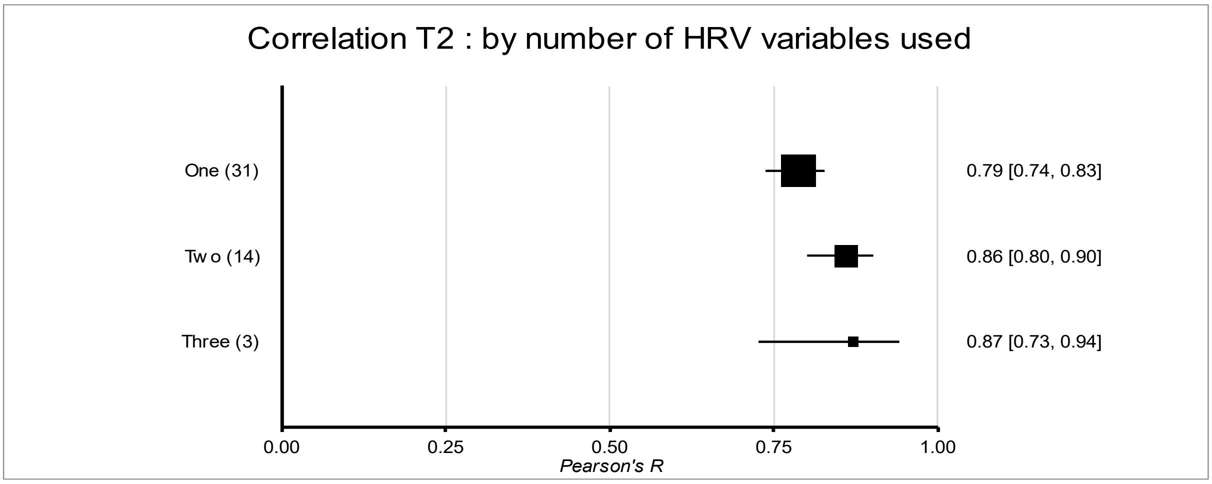

Between subgroups: p = 0.080

There is no difference in Pearson's R between subgroups.

Heterogeneity

	P-value	*I-squared (%)*
One	0.000	78.7
Two	0.000	72.5
Three	0.000	89.3

HRVT2 determination type:

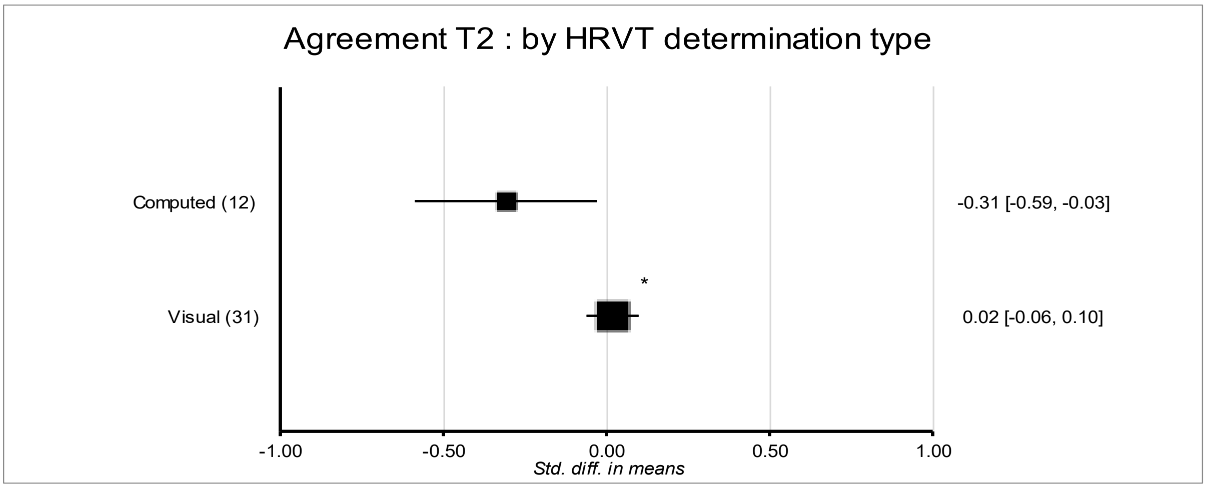

**p < 0.001 for difference with computed. HRVT, heart rate variability threshold.*

Between subgroups: **p = 0.0276**

The std. diff. in means are different between subgroups.

	Heterogeneity	
	P-value	*I-squared (%)*
Computed	0.000	91.8
Visual	0.000	80.4

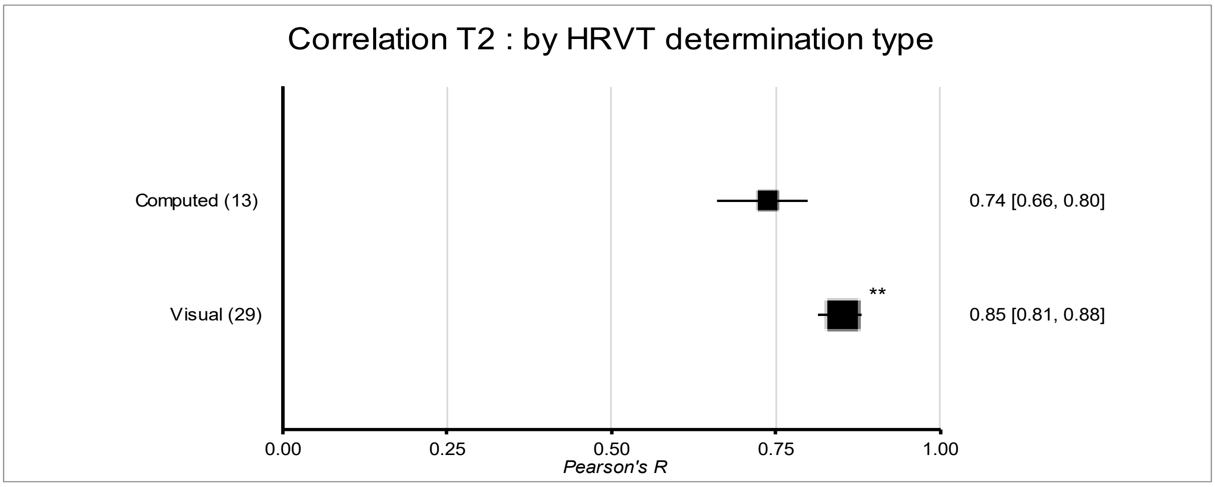

***p < 0.01 for difference with Computed. HRVT, heart rate variability threshold.*

Between subgroups: **p = 0.001**

The Pearson's R is different between subgroups.

	Heterogeneity	
	P-value	*I-squared (%)*
Computed	0.000	80.0
Visual	0.000	74.7

HRVT2 determination complexity:

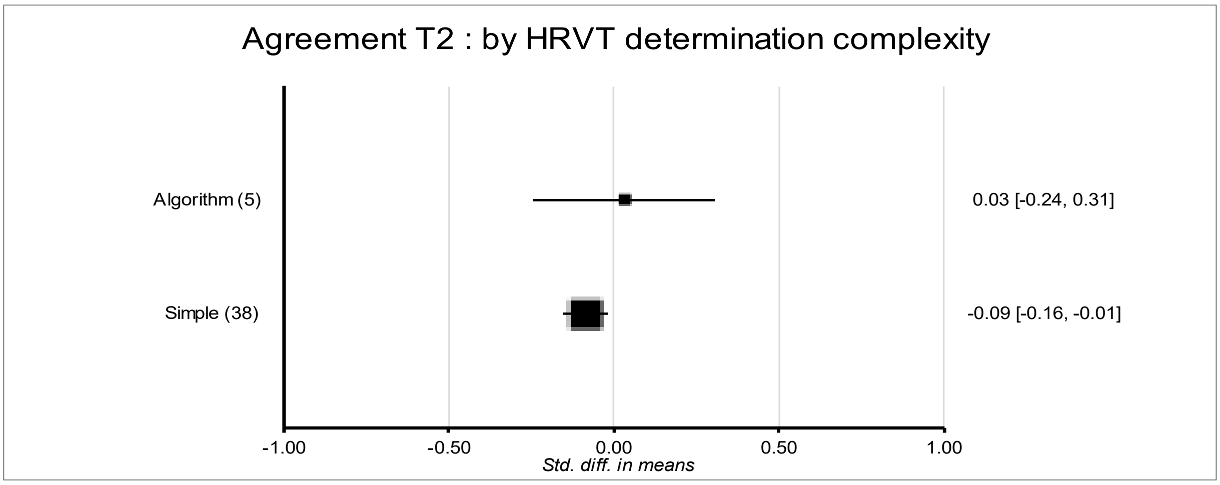

HRVT, heart rate variability threshold. Simple: HRVT determination made visually or, if calculated, by applying a fixed threshold to an HRV variable. Algorithm: HRVT determination requiring a more complex algorithm.

Between subgroups: p = 0.415

There is no difference in std. diff. in means between subgroups.

	Heterogeneity	
	P-value	***I-squared (%)***
Algorithm	0.290	19.0
Simple	0.000	86.7

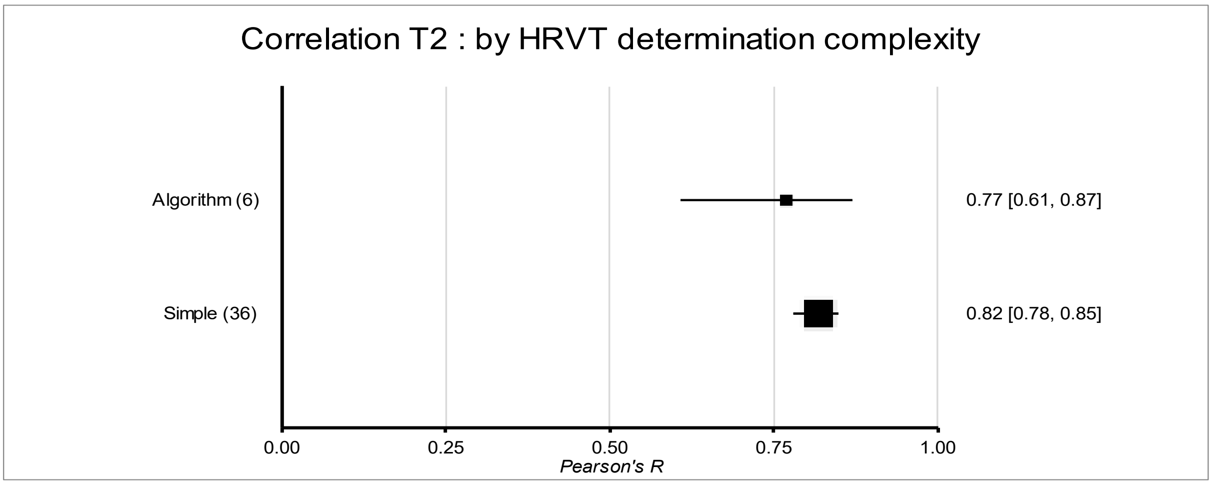

HRVT, heart rate variability threshold. Simple: HRVT determination made visually or, if calculated, by applying a fixed threshold to an HRV variable. Algorithm: HRVT determination requiring a more complex algorithm

Between subgroups: p = 0.437

There is no other difference in Pearson's R between subgroups.

	Heterogeneity	
	P-value	***I-squared (%)***
Algorithm	0.000	92.7
Non-algorithm	0.000	74.7

HRV recording device:

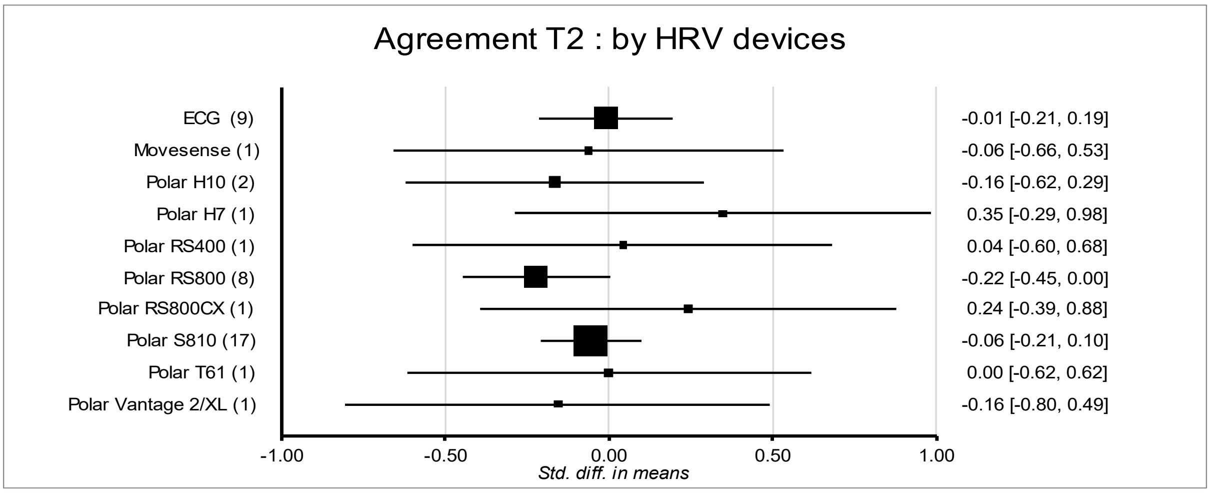

Between subgroups: p = 0.825

There is no difference in std. diff. in means between subgroups.

	Heterogeneity	
	P-value	*I-squared (%)*
ECG	0.070	44.7
Movesense	1	0.0
Polar H10	0.051	73.7
Polar H7	1	0.0
Polar RS400	1	0.0
Polar RS800	0.000	97.9
Polar RS800CX	1	0.0
Polar S810	0.000	92.0
Polar T61	1	0.0
Polar Vantage 2/XL	1	0.0

Correlation T2: by HRV recording devices on the next page

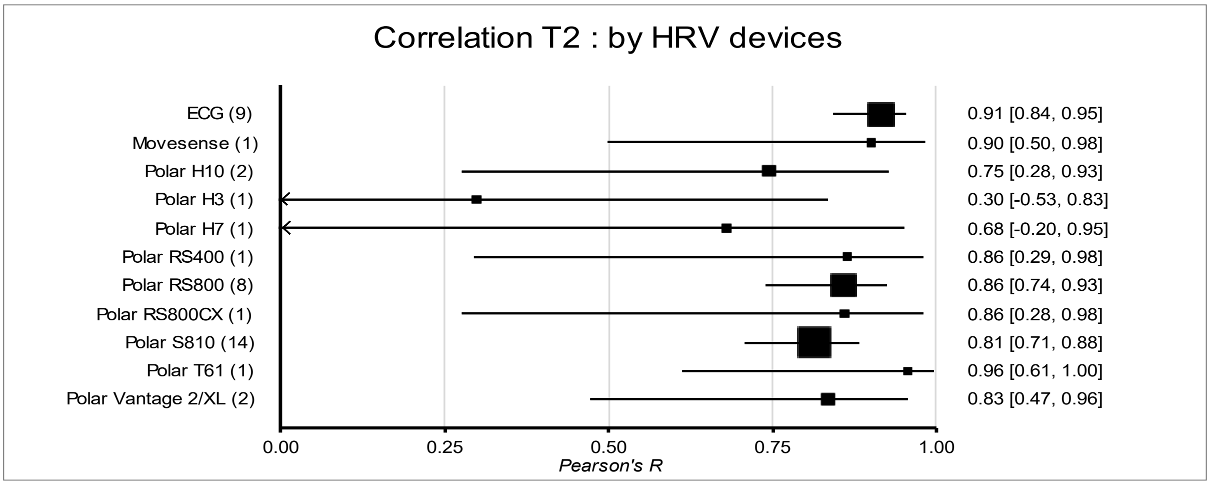

Between subgroups: p = 0.345

There is no difference in Pearson's R between subgroups.

	Heterogeneity	
	P-value	***I-squared (%)***
ECG (all type)	0.000	85.4
Movesense	1	0.0
Polar H10	0.002	89.2
Polar H3	1	0.0
Polar H7	1	0.0
Polar RS400	1	0.0
Polar RS800	0.000	91.7
Polar RS800CX	1	0.0
Polar S810	0.000	91.8
Polar T61	1	0.0
Polar Vantage 2/XL	0.542	0.0

HRV recording device type:

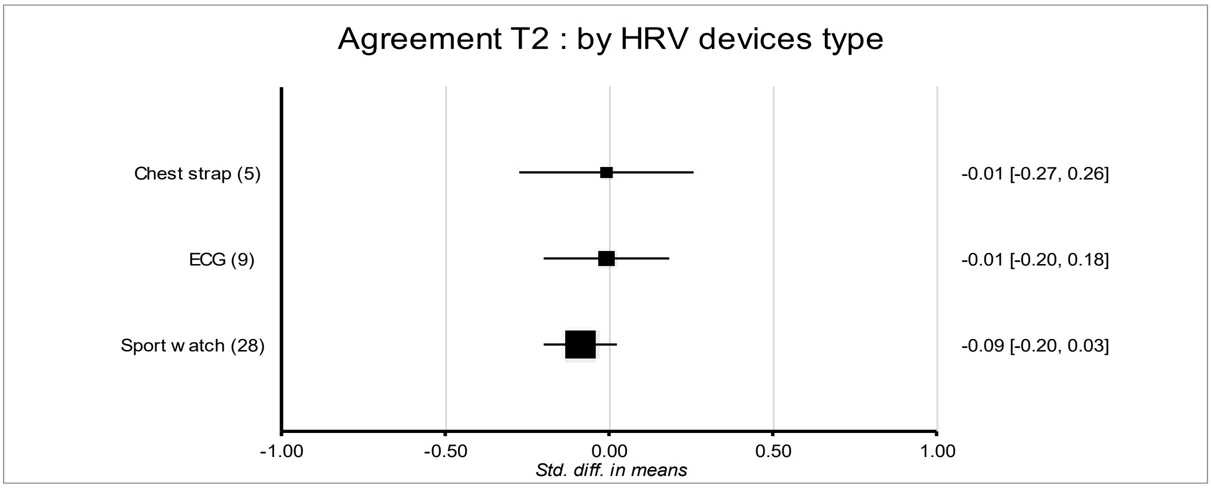

Between subgroups: p = 0.726

There is no difference in std. diff. in means between subgroups.

	Heterogeneity	
	P-value	*I-squared (%)*
Chest strap	0.008	71.2
ECG	0.070	44.7
Sport watch	0.000	94.9

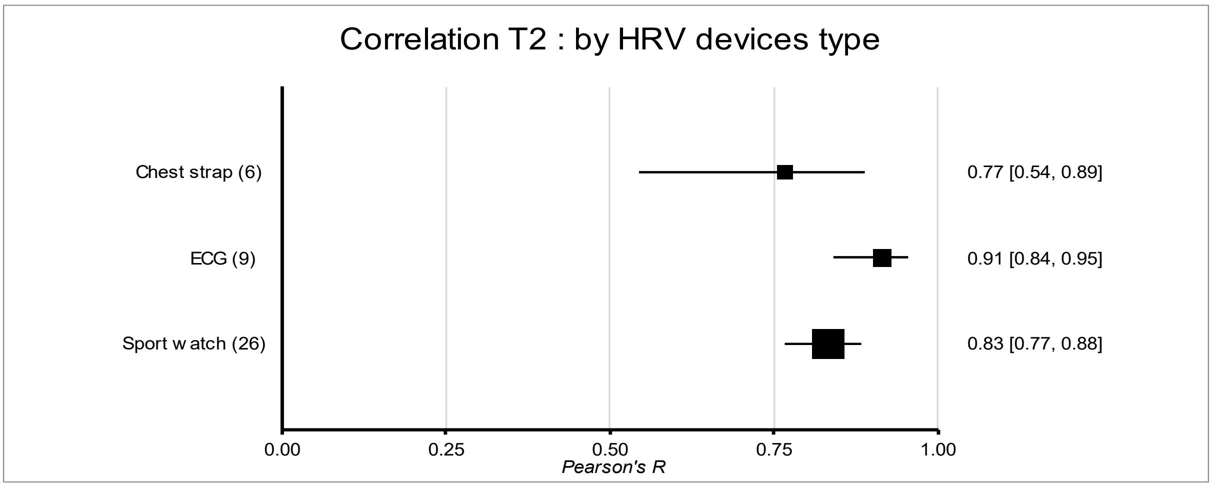

Between subgroups: p = 0.086

There is no difference in Pearson's R between subgroups.

	Heterogeneity	
	P-value	*I-squared (%)*
Chest strap	0.000	93.8
ECG	0.000	85.4
Sport watch	0.000	89.8

HRV Software:

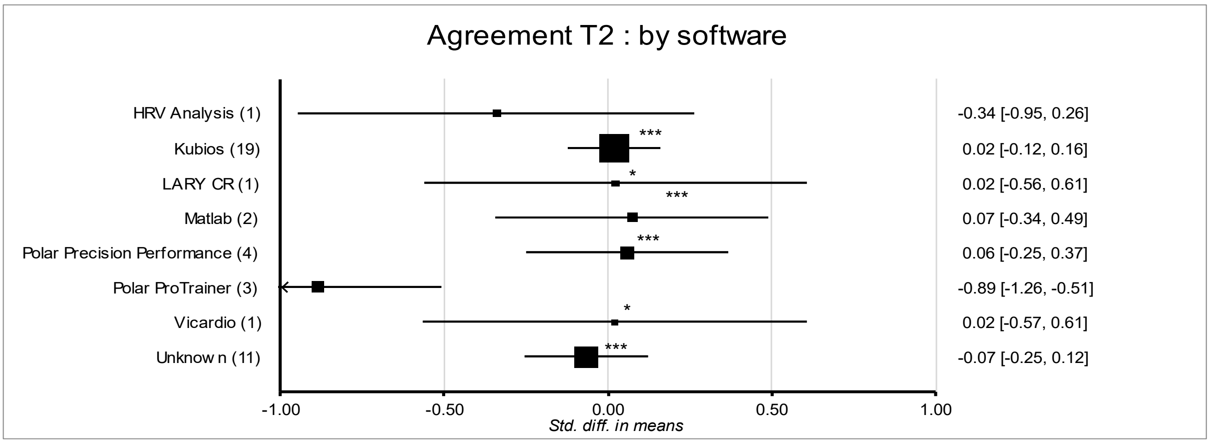

****p < 0.001 for difference with Polar ProTrainer*

Between groups: **p = 0.003**

Polar ProTrainer std. diff. in means is lower than:

- Kubios: p < 0.001
- LARY CR: p = 0.010
- Matlab: p < 0.001
- Polar precision performance: p < 0.001
- Vicardio: p = 0.011
- Unknown: p < 0.001

There is no other difference in std. diff. in means between subgroups.

	Heterogeneity	
	P-value	*I-squared (%)*
HRV Analysis	1	0.00
Kubios	1	0.00
LARY CR	0.000	90.90
Matlab	0.012	76.8
Polar Precision Performance	0.409	0.00
Polar ProTrainer	0.425	0.00
Vicardio	1	0.00
Unknown	0.000	99.27

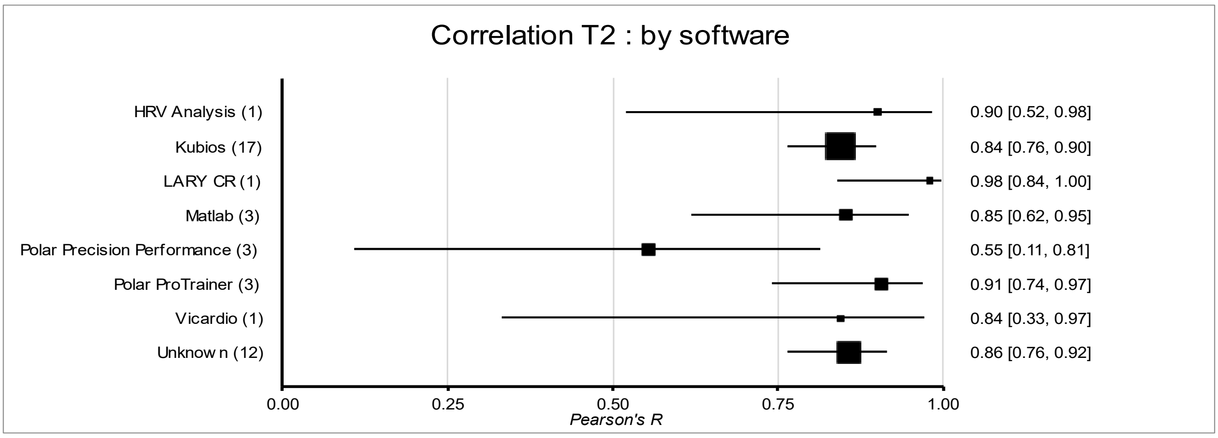

Between subgroups: p = 0.164

There is no difference in Pearson's R between subgroups.

	Heterogeneity	
	P-value	*I-squared (%)*
HRV Analysis	1	0.00
Kubios	0.000	88.66
LARY CR	1	0.00
Matlab	0.000	97.52
Polar Precision Performance	0.005	81.15
Polar ProTrainer	0.000	92.92
Vicardio	1	0.00
Unknown	0.000	69.67

Study protocol

Outcomes:

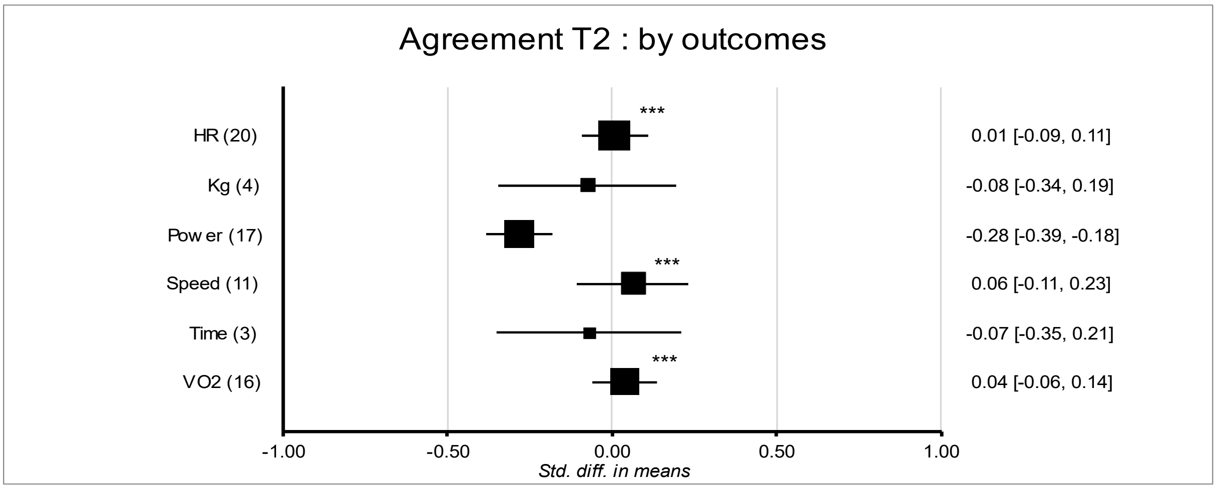

****p < 0.001 for difference with Power*

Between groups: **p < 0.001**

Power std. diff. in means is lower than:

- HR: p < 0.001
- Speed: p < 0.001
- VO2: p < 0.001

There is no other difference in std. diff. in means between subgroups.

	**Heterogeneity**	
	P-value	***I-squared (%)***
HR	0.000	82.09
Kg	0.962	0.00
Power	0.000	94.34
Speed	0.000	76.63
Time	0.051	46.55
VO2	0.000	71.86

Correlation T2: by Outcomes on next page

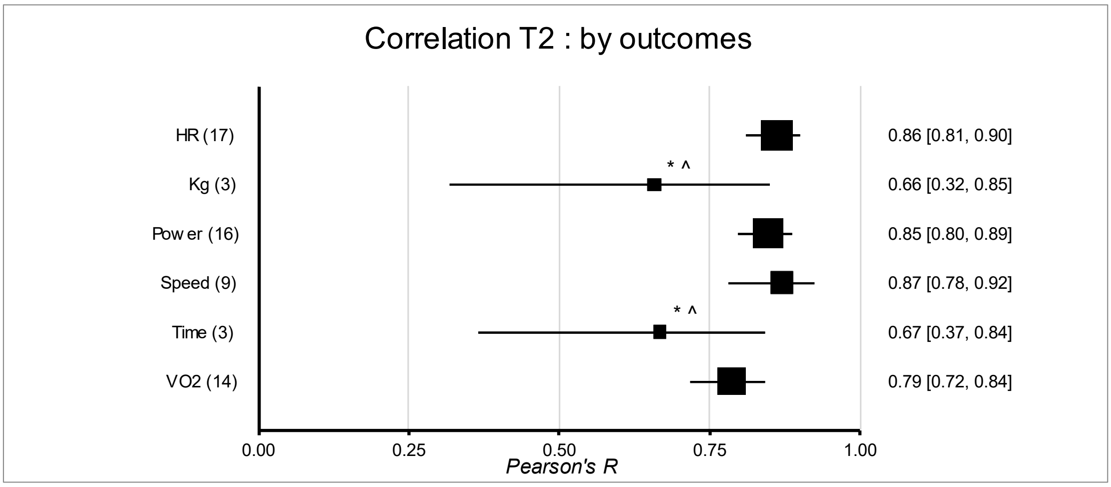

**p < 0.05 for difference with HR; ^p < 0.05 for difference with Speed*

Between groups: **p = 0.044**

HR Pearson's R is higher than:

- Kg: p = 0.043
- Time: p = 0.034

Speed Pearson's R is higher than:

- Kg: p = 0.048
- Time: p = 0.041

There is no other difference in Pearson's R between subgroups.

	**Heterogeneity**	
	P-value	***I-squared (%)***
HR	0.000	73.9
Kg	0.804	0.0
Power	0.000	74.2
Speed	0.000	84.1
Time	0.000	96.4
VO2	0.000	85.0

Outcomes format:

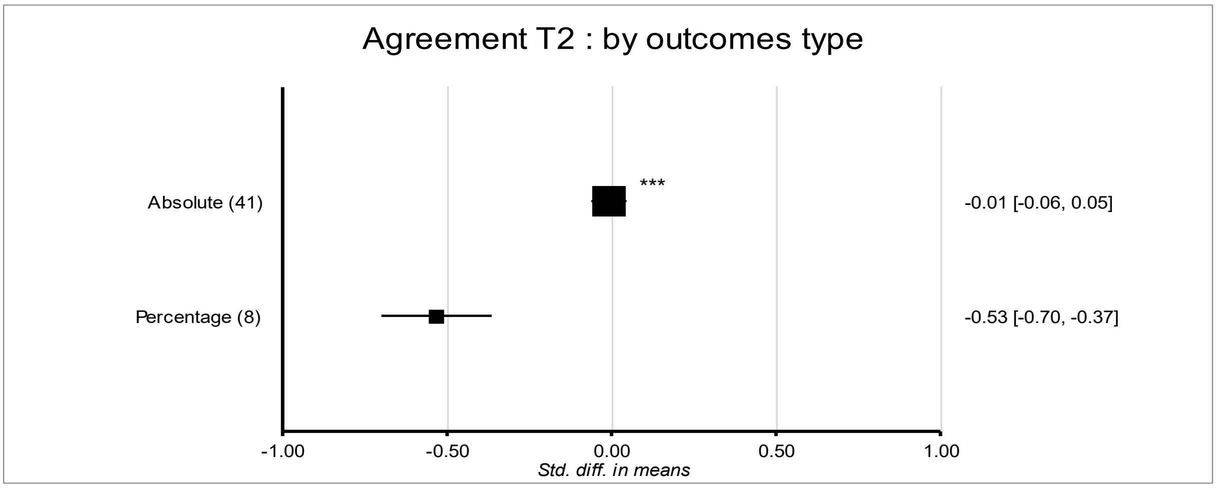

****p < 0.001 for difference with Percentage*

Between subgroups: **p < 0.001**

The std. diff. in means are different between subgroups.

	Heterogeneity	
	P-value	***I-squared (%)***
Absolute	0.000	94.00114672
Percentage	0.000	85.78439389

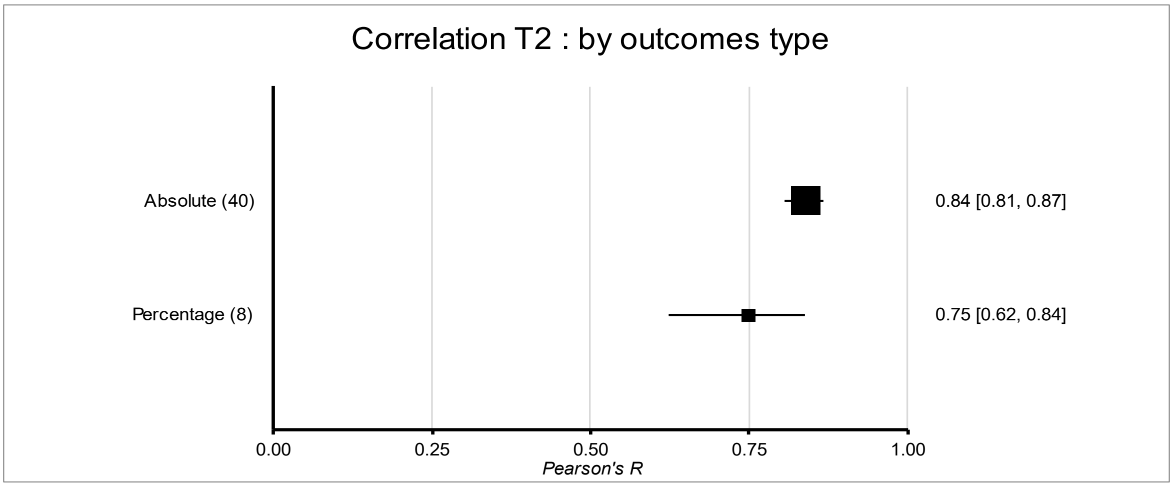

Between subgroups: p = 0.062

There is no difference in Pearson's R between subgroups.

	Heterogeneity	
	P-value	***I-squared (%)***
Absolute	0.000	84.1
Percentage	0.000	82.0

Ergometers:

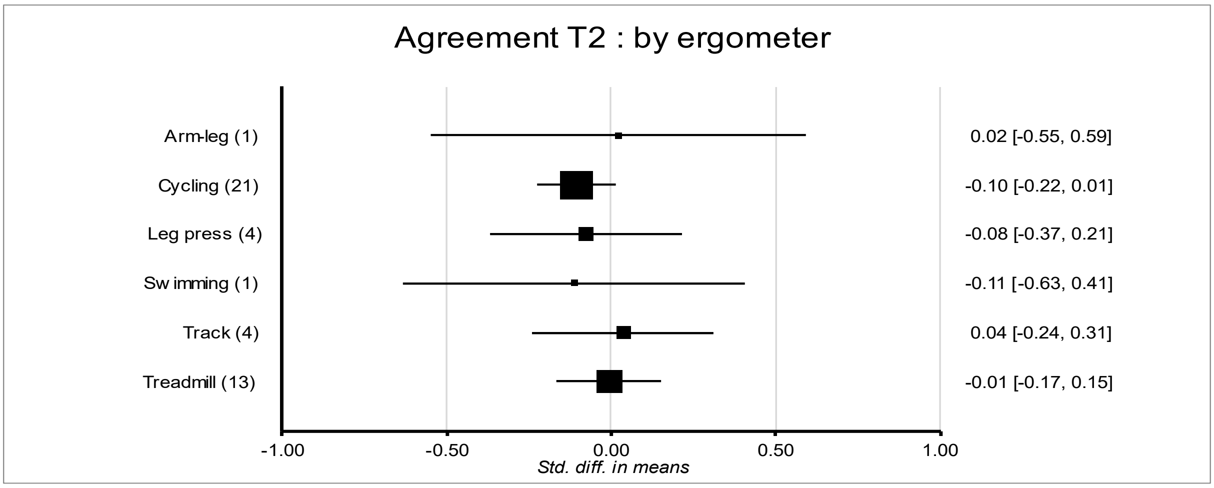

Between subgroups: p = 0.904

There is no difference in std. diff. in means between subgroups.

	Heterogeneity	
	P-value	*I-squared (%)*
Arm-leg	1	0.0
Cycling	0.000	94.6
Leg press	0.591	0.0
Swimming	1	0.0
Track	0.890	0.0
Treadmill	0.000	78.5

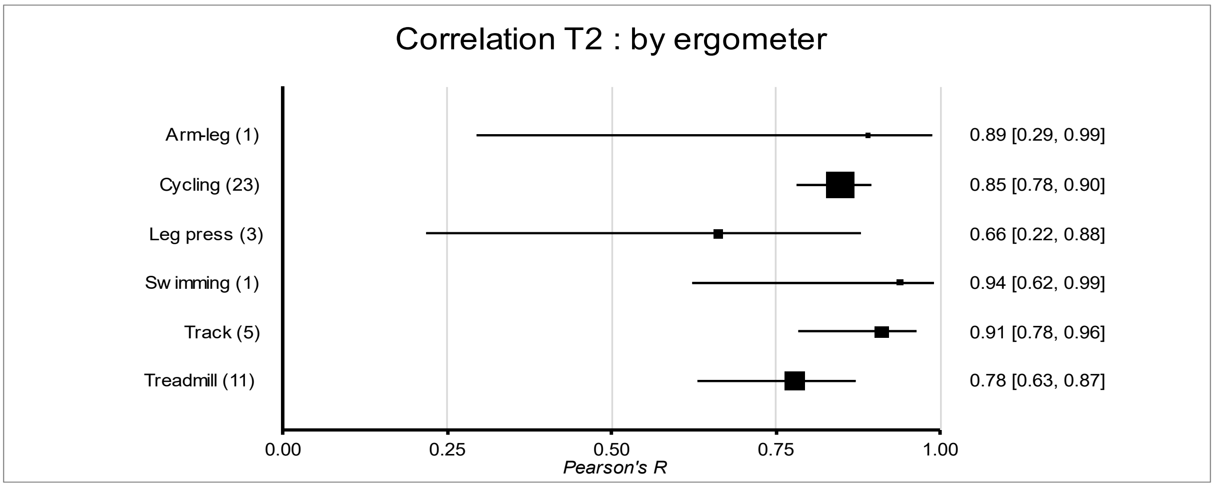

Between subgroups: p = 0.283

There is no difference in Pearson's R between subgroups.

	Heterogeneity	
	P-value	*I-squared (%)*
Arm-leg	1	0.0
Cycling	0.000	87.8
Leg press	0.328	10.2
Swimming	1	0.0
Track	0.000	96.5
Treadmill	0.000	88.4

Initial workload:

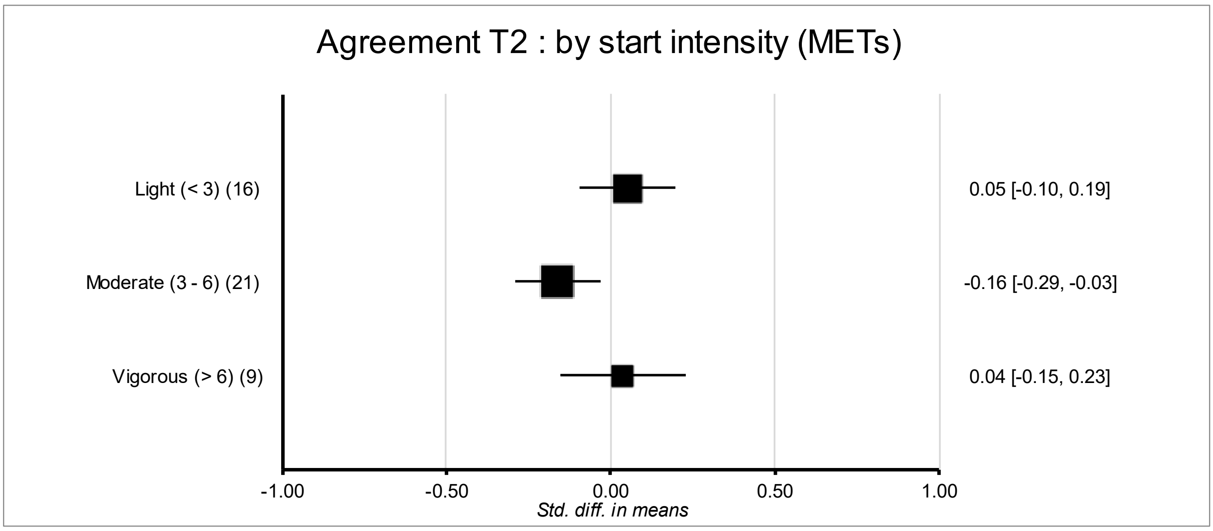

Between subgroups: p = 0.067

There is no difference in std. diff. in means between subgroups.

	Heterogeneity	
	P-value	*I-squared (%)*
Light (< 3)	0.000	82.6
Moderate (3 - 6)	0.000	93.9
Vigorous (> 6)	0.000	93.3

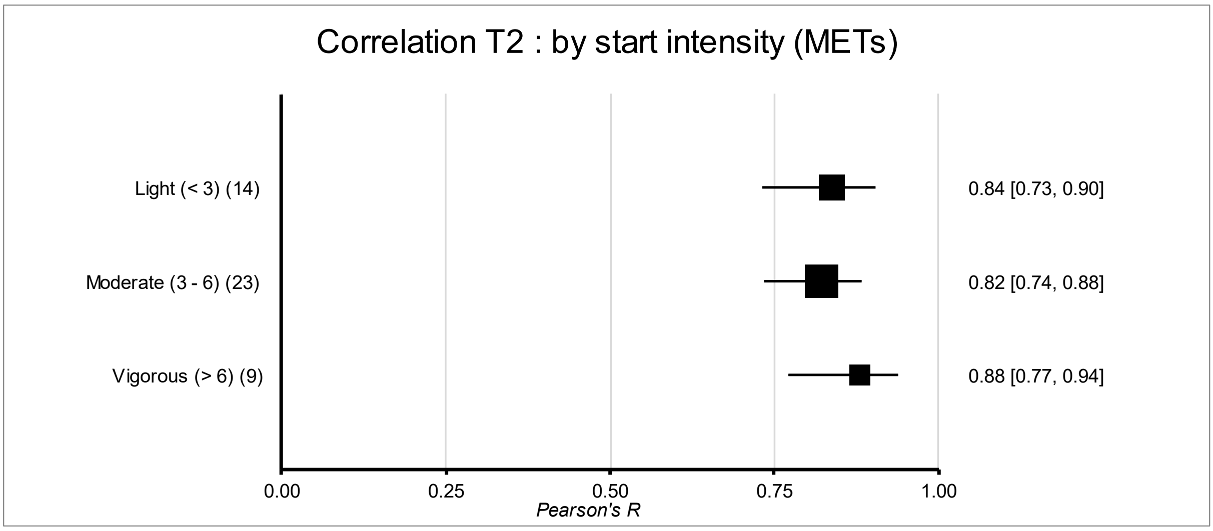

Between subgroups: p = 0.601

There is no difference in Pearson's R between subgroups.

	Heterogeneity	
	P-value	*I-squared (%)*
Light (< 3)	0.000	91.7
Moderate (3 - 6)	0.000	78.2
Vigorous (> 6)	0.000	96.4

Increment workload (METs):

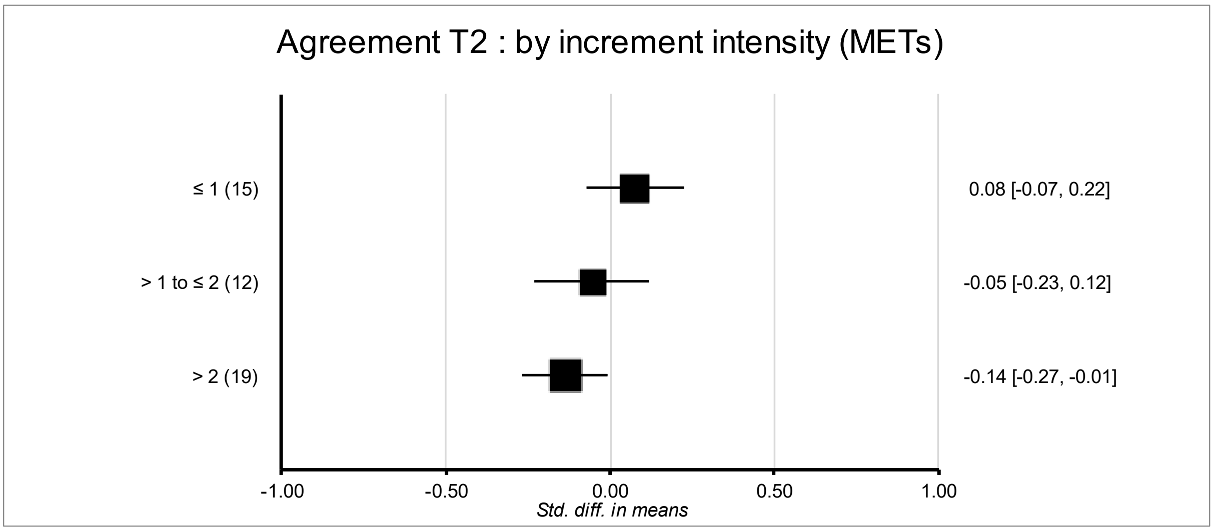

Between subgroups: p = 0.103

There is no difference in std. diff. in means between subgroups.

	Heterogeneity	
	P-value	*I-squared (%)*
≤ 1	0.010	51.8
> 1 to ≤ 2	0.000	91.3
> 2	0.000	95.1

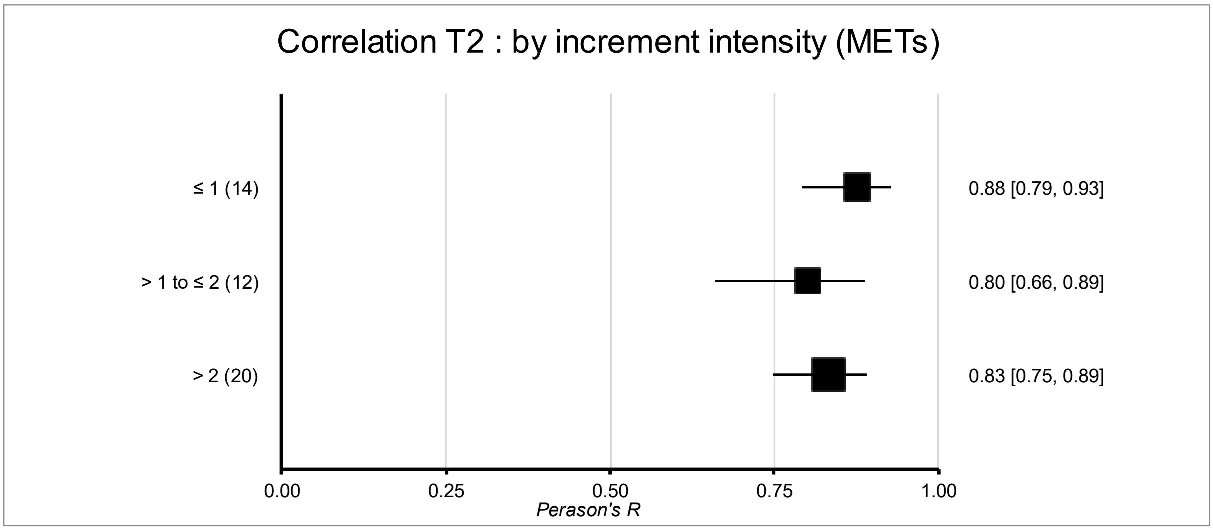

Between subgroups: p = 0.462

There is no difference in Pearson's R between subgroups.

	Heterogeneity	
	P-value	*I-squared (%)*
≤ 1	0.000	93.4
> 1 to ≤ 2	0.000	88.1
> 2	0.000	89.7

Increment workload (%):

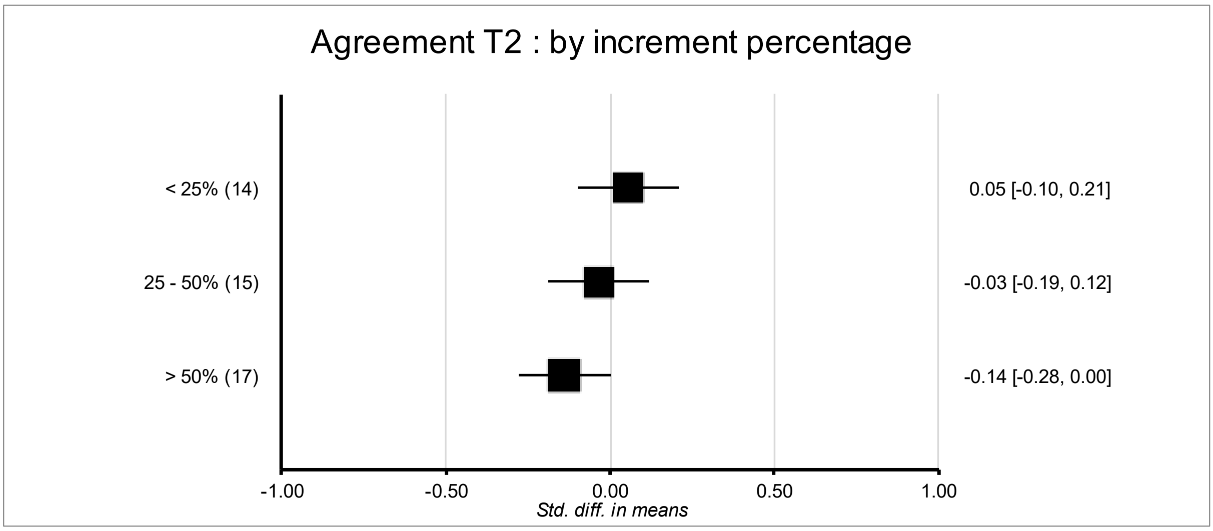

Between subgroups: p = 0.183

There is no difference in std. diff. in means between subgroups.

	Heterogeneity	
	P-value	*I-squared (%)*
< 25%	0.000	91.0
25 - 50%	0.001	61.6
> 50%	0.000	95.5

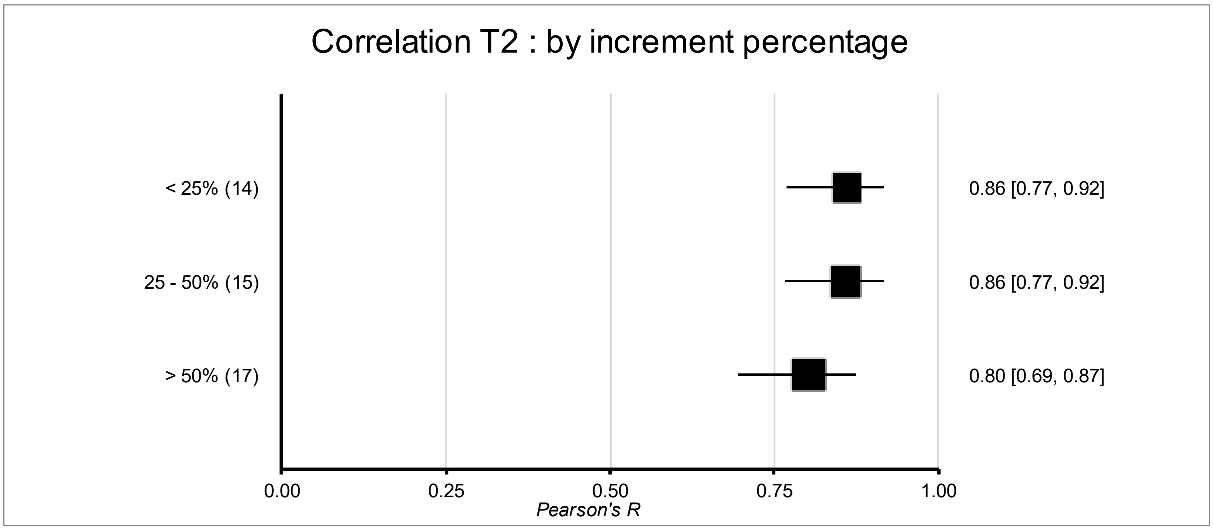

Between subgroups: p = 0.504

There is no difference in Pearson's R between subgroups.

	Heterogeneity	
	P-value	*I-squared (%)*
< 25%	0.000	94.1
25 - 50%	0.000	89.8
> 50%	0.000	89.8

Increment duration:

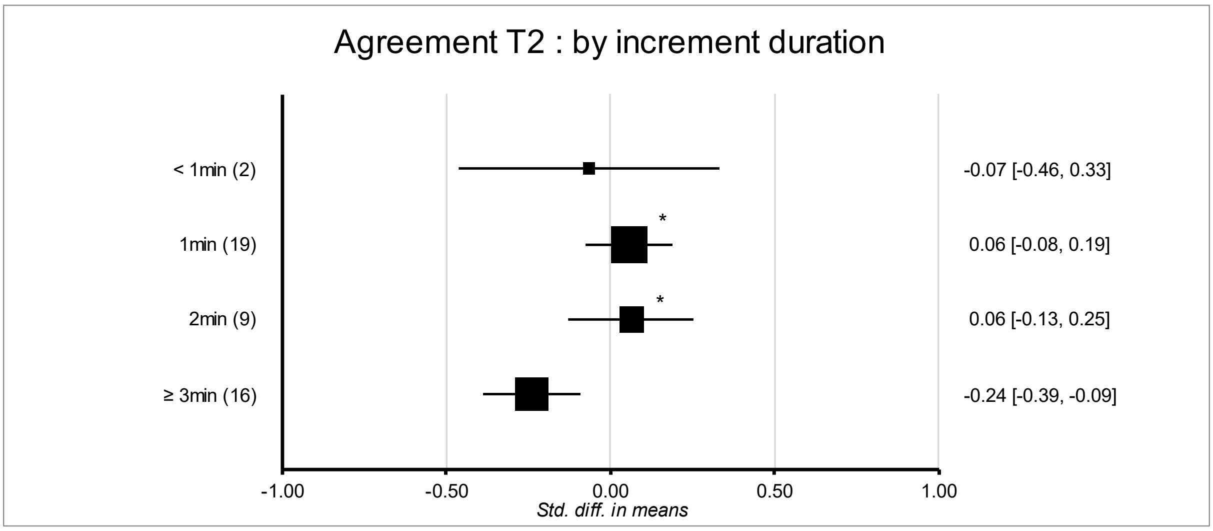

**p < 0.05 for difference with ≥ 3min*

Between subgroups: **p = 0.018**

≥ 3min std. diff. in means is lower than:

- 1min: p = 0.036
- 2min: P = 0.014

There is no other difference in std. diff. in means between subgroups.

	Heterogeneity	
	P-value	*I-squared (%)*
< 1min	0.961	0.0
1min	0.000	71.6
2min	0.000	91.4
≥ 3min	0.000	95.6

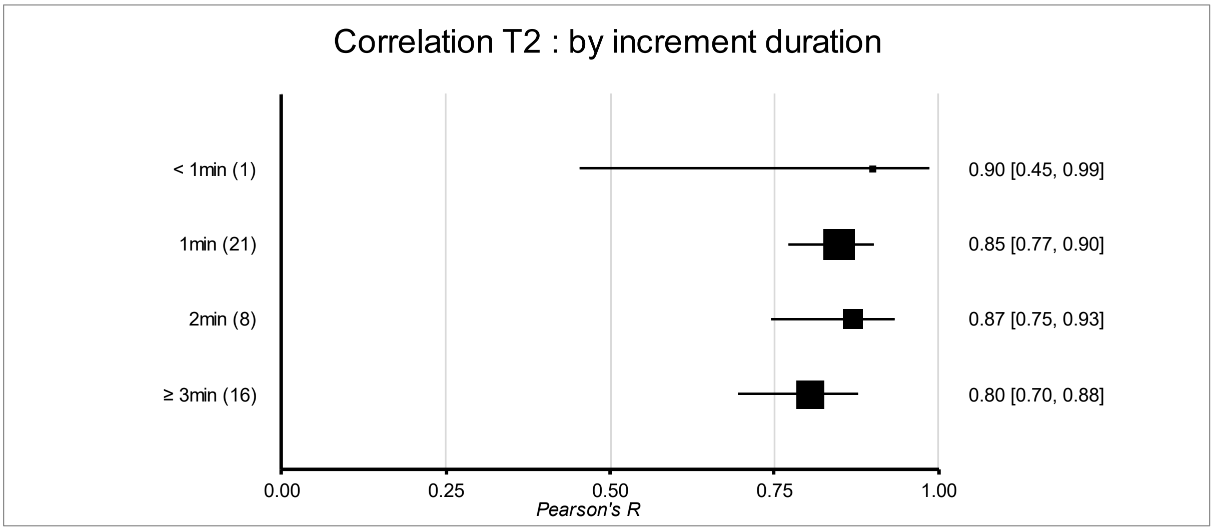

Between subgroups: p = 0.716

There is no difference in Pearson's R between subgroups.

	Heterogeneity	
	P-value	*I-squared (%)*
< 1min	1	0.0
1min	0.000	91.8
2min	0.000	86.8
≥ 3min	0.000	90.4

Continent:

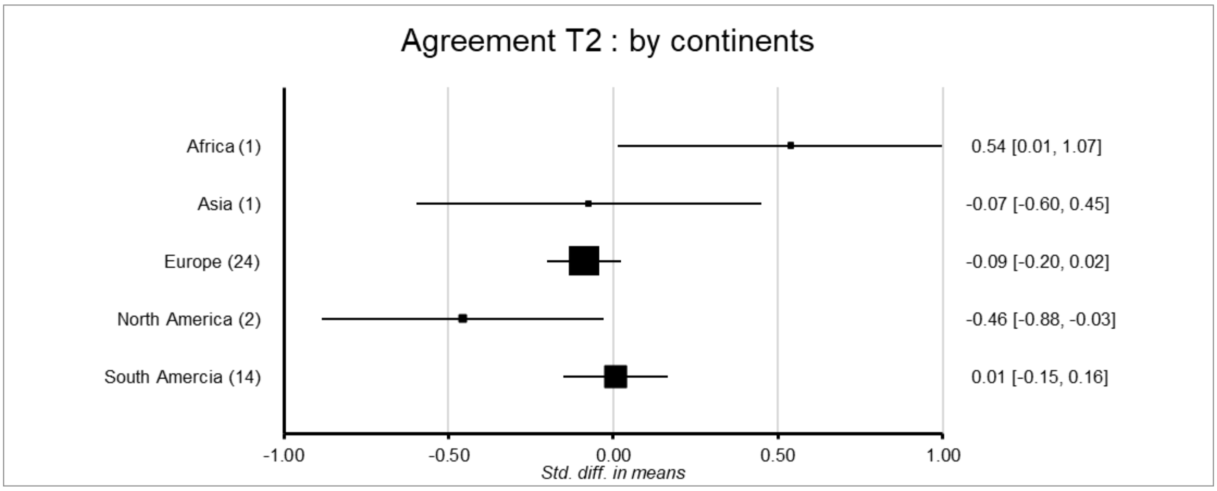

Between subgroups: p = 0.056

There is no difference in std. diff. in means between subgroups.

	Heterogeneity	
	P-value	*I-squared (%)*
Africa	1	0.0
Asia	1	0.0
Europe	0.000	93.1
North America	0.000	93.0
South America	0.000	81.7

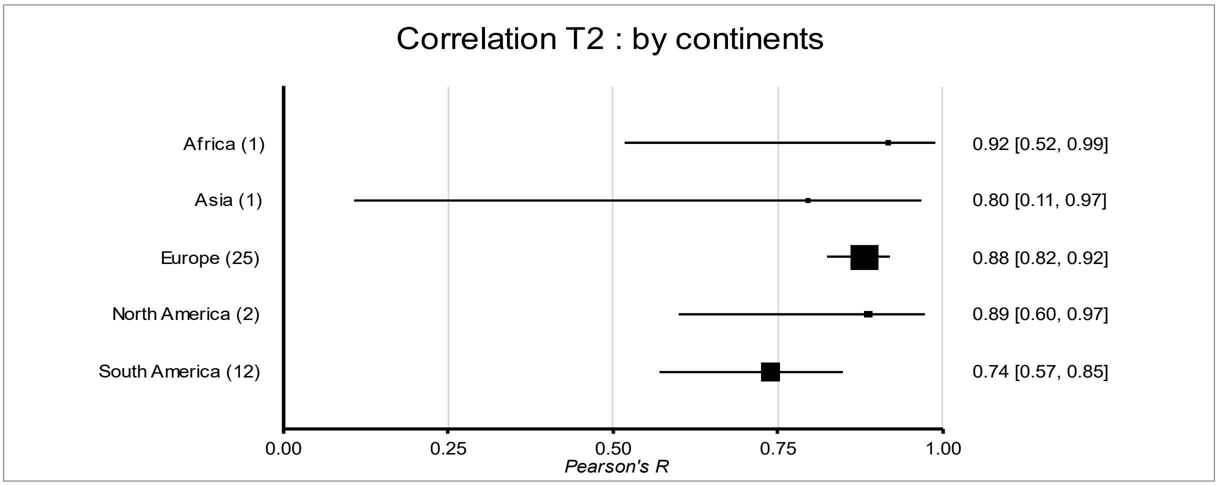

Between subgroups: p = 0.200

There is no difference in Pearson's R between subgroups.

	Heterogeneity	
	P-value	*I-squared (%)*
Africa	1	0.0
Asia	1	0.0
Europe	0.000	92.5
North America	0.230	30.5
South America	0.000	86.2
