# Supplementary material for: Agreement Between Heart Rate Variability - Derived vs. Ventilatory and Lactate Thresholds: A Systematic Review with Meta-Analyses
Source: Sports Med Open. 2024 Oct 8;10:109. doi: 10.1186/s40798-024-00768-8 (PMC11461412; doi:10.1186/s40798-024-00768-8)
Supplement: Supplementary file 1 — Supplementary Material 1: Peer-reviewed search strategies [file 40798_2024_768_MOESM1_ESM.pdf]

Agreement between heart rate variability - derived vs. ventilatory and lactate thresholds: A systematic review with meta-analyses

Electronic Supplementary Material 1

Peer-reviewed search strategies

Sports Medicine - Open

Valérian Tanner[1]*, Grégoire P. Millet[1], Nicolas Bourdillon[1]

[1]Institute of Sport Sciences, University of Lausanne, Lausanne, Switzerland.

***Corresponding author:**
- E-mail: valerian.tanner@unil.ch
- Adress: Quartier UNIL-Centre, Bâtiment Synathlon, 1015 Lausanne, Switzerland.

1 Cochrane Library 3
2 EBSCO 4
3 Embase.com 5
4 Google Scholar 6
5 Ovid 7
6 ProQuest 8
7 PubMed 9
8 Scopus 10
9 SportDiscus 11
10 Virtual Health Library 12
11 Web of science 13

1 Cochrane Library

Search strategy:

(("heart rate variability" OR "heart rate variabilities" OR "heartrate variability" OR "heartrate variabilities" OR HRV OR "detrended fluctuation analysis" OR "detrended fluctuation analyses" OR DFA OR "time varying analysis" OR "time varying analyses" OR "fractal correlation property" OR "fractal correlation properties" OR "recurrence quantification analysis" OR "recurrence quantification analyses") AND ("ventilatory threshold" OR "ventilatory thresholds" OR "lactate threshold" OR "lactate thresholds" OR "aerobic threshold" OR "aerobic thresholds" OR "anaerobic threshold" OR "anaerobic thresholds" OR "intensity threshold" OR "intensity thresholds")) OR ("heart rate variability threshold" OR "heart rate variability thresholds" OR "heartrate variability threshold" OR "heartrate variability thresholds" OR HRVT OR HRVTS OR HRVT1 OR HRVT2)

Search date: 07.08.23

Results: n=64

Search in: Title, Abstract, Keywords

Databases: N/A

Mail alert: unavailable

2 EBSCO

Search strategy:

TI ((("heart rate variabilit*" OR "heartrate variabilit*" OR HRV OR "detrended fluctuation analys*" OR DFA OR "time varying analys*" OR "fractal correlation propert*" OR "recurrence quantification analys*") AND ("ventilatory threshold*" OR "lactate threshold*" OR "aerobic threshold*" OR "anaerobic threshold*" OR "intensity threshold*")) OR ("heart rate variability threshold*" OR "heartrate variability threshold*" OR HRVT OR HRVTS OR HRVT1 OR HRVT2)) OR AB ((("heart rate variabilit*" OR "heartrate variabilit*" OR HRV OR "detrended fluctuation analys*" OR DFA OR "time varying analys*" OR "fractal correlation propert*" OR "recurrence quantification analys*") AND ("ventilatory threshold*" OR "lactate threshold*" OR "aerobic threshold*" OR "anaerobic threshold*" OR "intensity threshold*")) OR ("heart rate variability threshold*" OR "heartrate variability threshold*" OR HRVT OR HRVTS OR HRVT1 OR HRVT2))

Search date: 07.08.23

Results: n=203

Search in: see search strategy

Databases: MEDLINE; CINAHL with Full Text; ERIC; SocINDEX with Full text

Mail alert: set up

3 Embase.com

Search strategy:

(('heart rate variability'/exp OR 'heart rate variabilit*' OR 'heartrate variabilit*' OR hrv OR 'detrended fluctuation analysis'/exp OR 'detrended fluctuation analys*' OR DFA OR 'time varying analys*' OR 'fractal correlation propert*' OR 'recurrence quantification analysis'/exp OR 'recurrence quantification analys*') AND ('ventilatory threshold*' OR 'lactate threshold*' OR 'aerobic threshold*' OR 'anaerobic threshold*' OR 'intensity threshold*')) OR 'heart rate variability threshold*' OR 'heartrate variability threshold*' OR HRVT OR HRVTS OR HRVT1 OR HRVT2

Search date: 07.08.23

Results: n=241

Search in: N/A

Databases: N/A

Mail alert: set up

4 Google Scholar

Search strategy:

("heart rate variability|variabilities"|"detrended fluctuation analysis|analyses"|"time varying analysis|analyses"|"fractal correlation"|"recurrence quantification") AND ("ventilatory|lactate|aerobic|anaerobic treshold|tresholds"|treshold)

Search date: 07.08.23

Results: n≈19’600

Search in: N/A

Databases: N/A

Mail alert: set up

As recommended, the first 300 articles obtained from the google scholar search were considered [1].

1. Haddaway NR, Collins AM, Coughlin D, Kirk S. The Role of Google Scholar in Evidence Reviews and Its Applicability to Grey Literature Searching. PLOS ONE. 2015;10:e0138237.

5 Ovid

Search strategy:

((("heart rate variabilit*" or "heartrate variabilit*" or HRV or "detrended fluctuation analys*" or DFA or "time varying analys*" or "fractal correlation propert*" or "recurrence quantification analys*") and ("ventilatory threshold*" or "lactate threshold*" or "aerobic threshold*" or "anaerobic threshold*" or "intensity threshold*")) or ("heart rate variability threshold*" or "heartrate variability threshold*" or HRVT or HRVTS or HRVT1 or HRVT2)).ab,kw,ti.

Search date: 07.08.23

Results: n=263

Search in: see search strategy

Databases: Journals@Ovid Full Text and Abstracts; Lausanne Journals@Ovid Full Text Collection; AGRICOLA; APA PsycInfo; Ovid MEDLINE(R) and Epub Ahead of Print, In-Process, In-Data-Review & Other Non-Indexed Citations, Daily and Versions

Mail alert: unavailable

6 ProQuest

Search strategy:

noft((("heart rate variabilit*" OR "heartrate variabilit*" OR HRV OR "detrended fluctuation analys*" OR DFA OR "time varying analys*" OR "fractal correlation propert*" OR "recurrence quantification analys*") AND ("ventilatory threshold*" OR "lactate threshold*" OR "aerobic threshold*" OR "anaerobic threshold*" OR "intensity threshold*")) OR ("heart rate variability threshold*" OR "heartrate variability threshold*" OR HRVT OR HRVTS OR HRVT1 OR HRVT2))

Search date: 07.08.23

Results: n=305

Search in: see search strategy

Databases: MEDLINE®; Publicly Available Content Database; ProQuest Dissertations & Theses Global; ProQuest Dissertations & Theses Global: Health & Medicine; ProQuest Dissertations & Theses Global: Social Sciences; ProQuest Dissertations & Theses Global: Science & Technology; ABI/INFORM Collection; ABI/INFORM Trade & Industry; ProQuest Dissertations & Theses Global: The Arts; ProQuest Dissertations & Theses Global: Business; ProQuest Dissertations & Theses Global: History; ProQuest Dissertations & Theses Global: Literature & Language; ProQuest One Literature; ProQuest Historical Newspapers: The New York Times with Index; ABI/INFORM Dateline; Coronavirus Research Database

Mail alert: set up

7 PubMed

Search strategy:

(("heart rate variabilit*"[Title/Abstract] OR "heartrate variabilit*"[Title/Abstract] OR HRV[Title/Abstract] OR "detrended fluctuation analys*"[Title/Abstract] OR DFA[Title/Abstract] OR "time varying analys*"[Title/Abstract] OR "fractal correlation propert*"[Title/Abstract] OR "recurrence quantification analys*"[Title/Abstract]) AND ("ventilatory threshold*"[Title/Abstract] OR "lactate threshold*"[Title/Abstract] OR "aerobic threshold*"[Title/Abstract] OR "anaerobic threshold*"[Title/Abstract] OR "intensity threshold*"[Title/Abstract])) OR ("heart rate variability threshold*"[Title/Abstract] OR "heartrate variability threshold*"[Title/Abstract] OR HRVT[Title/Abstract] OR HRVTS[Title/Abstract] OR HRVT1[Title/Abstract] OR HRVT2[Title/Abstract])

Search date: 07.08.23

Results: n=178

Search in: see search strategy

Databases: N/A

Mail alert: set up

8 Scopus

Search strategy:

TITLE-ABS-KEY((("heart rate variabilit*" OR "heartrate variabilit*" OR HRV OR "detrended fluctuation analys*" OR DFA OR "time varying analys*" OR "fractal correlation propert*" OR "recurrence quantification analys*") AND ("ventilatory threshold*" OR "lactate threshold*" OR "aerobic threshold*" OR "anaerobic threshold*" OR "intensity threshold*")) OR ("heart rate variability threshold*" OR "heartrate variability threshold*" OR HRVT OR HRVTS OR HRVT1 OR HRVT2))

Search date: 07.08.23

Results: n=288

Search in: see search strategy

Databases: N/A

Mail alert: set up

9 SportDiscus

Search strategy:

(("heart rate variabilit*" OR "heartrate variabilit*" OR HRV OR "detrended fluctuation analys*" OR DFA OR "time varying analys*" OR "fractal correlation propert*" OR "recurrence quantification analys*") AND ("ventilatory threshold*" OR "lactate threshold*" OR "aerobic threshold*" OR "anaerobic threshold*" OR "intensity threshold*")) OR ("heart rate variability threshold*" OR "heartrate variability threshold*" OR HRVT OR HRVTS OR HRVT1 OR HRVT2)

Search date: 22.02.23

Results: n=108

Search in: N/A

Databases: N/A

Mail alert: set up

10 Virtual Health Library

Search strategy:

(("heart rate variability" OR "heart rate variabilities" OR "heartrate variability" OR "heartrate variabilities" OR HRV OR "detrended fluctuation analysis" OR "detrended fluctuation analyses" OR DFA OR "time varying analysis" OR "time varying analyses" OR "fractal correlation property" OR "fractal correlation properties" OR "recurrence quantification analysis" OR "recurrence quantification analyses") AND ("ventilatory threshold" OR "ventilatory thresholds" OR "lactate threshold" OR "lactate thresholds" OR "aerobic threshold" OR "aerobic thresholds" OR "anaerobic threshold" OR "anaerobic thresholds" OR "intensity threshold" OR "intensity thresholds")) OR ("heart rate variability threshold" OR "heart rate variability thresholds" OR "heartrate variability threshold" OR "heartrate variability thresholds" OR HRVT OR HRVTS OR HRVT1 OR HRVT2)

Search date: 07.08.23

Results: n=226

Search in: Title, Abstract, Subjects

Databases: N/A

Mail alert: unavailable

11 Web of science

Search strategy:

TS=((("heart rate variabilit*" OR "heartrate variabilit*" OR HRV OR "detrended fluctuation analys*" OR DFA OR "time varying analys*" OR "fractal correlation propert*" OR "recurrence quantification analys*") AND ("ventilatory threshold*" OR "lactate threshold*" OR "aerobic threshold*" OR "anaerobic threshold*" OR "intensity threshold*")) OR ("heart rate variability threshold*" OR "heartrate variability threshold*" OR HRVT OR HRVTS OR HRVT1 OR HRVT2))

Search date: 07.08.23

Results: n=386

Search in: see search strategy

Databases: Web of Science Core Collection; MEDLINE®; Current Contents Connect; BIOSIS Citation Index; SciELO Citation Index; Data Citation Index; Derwent Innovations Index; KCI-Korean Journal Database; Preprint Citation Index

Mail alert: set up
